# Design, Synthesis, Antitumour Evaluation, and In Silico Studies of Pyrazolo-[1,5-*c*]quinazolinone Derivatives Targeting Potential Cyclin-Dependent Kinases

**DOI:** 10.3390/molecules28186606

**Published:** 2023-09-13

**Authors:** Danyang Zheng, Chenqi Yang, Xiaogang Li, Dong Liu, Yan Wang, Xuesong Wang, Xueying Zhang, Yinfeng Tan, Yuchen Zhang, Youbin Li, Junyu Xu

**Affiliations:** Key Laboratory of Tropical Translational Medicine of Ministry of Education, Hainan Key Laboratory for Research and Development of Tropical Herbs, Haikou Key Laboratory of Li Nationality Medicine, School of Pharmacy, Hainan Medical University, Haikou 571199, China; zhengdy2335@163.com (D.Z.); cqyang2000@outlook.com (C.Y.); llg178701425@163.com (X.L.);

**Keywords:** quinazolinone, molecular hybridisation, dipolar cycloaddition, antitumour agents, cyclin-dependent kinases, in silico studies

## Abstract

An efficient, straightforward, and metal-free methodology to rapidly access functionalised pyrazolo-[1,5-c]quinazolinones via a [3 + 2] dipolar cycloaddition and regioselective ring expansion process was developed. The synthesised compounds were characterised by methods such as NMR, HRMS, and HPLC. The in vitro antiproliferative activity against A549 cells (non-small cell lung cancer) was significant for compounds 4i, 4m, and 4n with IC_50_ values of 17.0, 14.2, and 18.1 μM, respectively. In particular, compounds 4t and 4n showed inhibitory activity against CDK9/2. Predicted biological target and molecular modelling studies suggest that the compound 4t may target CDKs for antitumour effects. The synthesised derivatives were considered to have moderate drug-likeness and sufficient safety in silico. In summary, a series of pyrazolo-[1,5-c]quinazolinone derivatives with antitumour activity is reported for the first time. We provide not only a simple and efficient synthetic method but also helpful lead compounds for the further development of novel cyclin-dependent kinase (CDK) inhibitors.

## 1. Introduction

**Figure 1:**
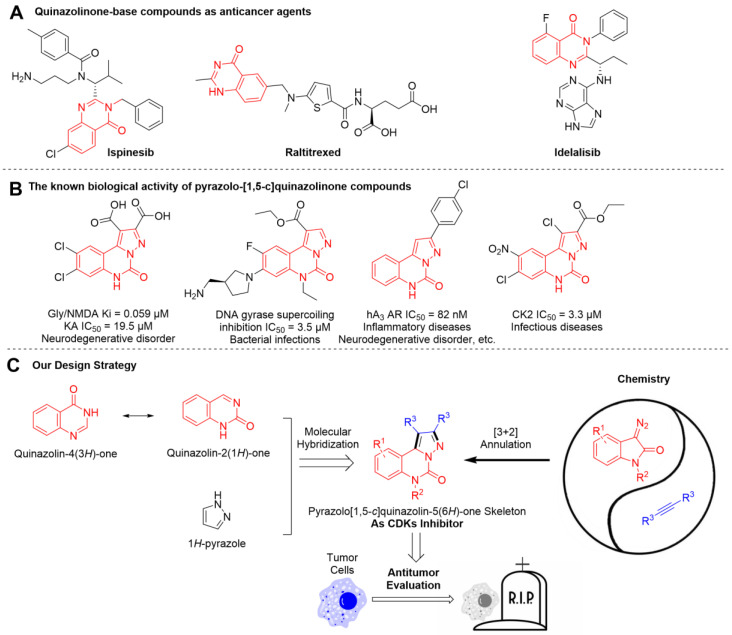
(**A**) Representative structures of quinazolinone derivatives as anticancer agents previously reported. (**B**) Some biologically active molecules containing a pyrazolo-[1,5-*c*]quinazolinone skeleton. (**C**) The design idea and synthesis strategy of the pyrazolo-[1,5-*c*]quinazolinone skeleton.

Quinazolinone [1,2] moieties are widely recognised as crucial structural components in the development of antineoplastic drugs. These compounds have been incorporated into various drug classes and have demonstrated efficacy through a diverse range of mechanisms. Research has shown that some compounds with a quinazoline core can inhibit cell proliferation, induce apoptosis, and impede angiogenesis, and they have the ability to modulate signal transduction pathways and target specific proteins [3,4,5,6,7,8,9]. As shown in Figure 1A, ispinesib [10,11,12] is a potent inhibitor of kinesin spindle protein (KSP), which plays a role as a key regulator of cell mitosis. By inhibiting KSP, ispinesib disrupts cell division and hinders the growth and spread of cancer cells. It is evaluated in phase II clinical trials for the treatment of various cancers. Raltitrexed [13,14] is a folate analogue thymidylate synthase inhibitor used in the treatment of advanced colorectal cancer. It inhibits thymidylate synthase (TS) leading to DNA fragmentation and cell death. Idelalisib [15,16] is a phosphoinositide 3-kinase (PI3K) inhibitor used to treat certain types of cancer such as chronic lymphocytic leukaemia (CLL), relapsed follicular B-cell non-Hodgkin lymphoma (FL), and relapsed small lymphocytic lymphoma (SLL). It works by slowing or stopping the growth of cancer cells. As such, these compounds containing quinazolinone moieties have played a significant role in the development of effective antineoplastic drugs.

In light of the significant antitumour activities demonstrated by quinazolinone-based compounds, our research endeavours have been directed towards the development of a lead compound incorporating pyrazolo-[1,5-*c*]quinazolinones, formed through the N-fusion of quinazolinones with pyrazoles [17,18]. It is noteworthy that quinazolinone–pyrazole hybrids have exhibited a range of biological activities, which may extend to potential therapeutic applications in neurodegenerative disorders [19,20,21], bacterial infections [22,23], inflammatory diseases [21,24], and neoplasia [23], among others [25]. Molecular hybridisation has recently emerged as a potent approach for addressing multifaceted diseases such as cancer and neurodegenerative disorders [26,27,28,29]. This innovative drug discovery strategy involves the integration of two or more pharmacophores into a single hybrid molecule, with the intent of generating novel chemical entities that display superimposed pharmacological effects. Our hope is to capitalise on the putative synergistic effects and enhanced pharmacological properties arising from the combination of quinazolinones and pyrazoles, in the pursuit of innovative antineoplastic agents. It is crucial to recognise that, to the best of our knowledge, the pyrazolo-[1,5-*c*]quinazolinone scaffold has been infrequently reported to exhibit antitumour activity. Consequently, in an effort to establish a novel foundation for the development of prospective anticancer therapeutics, we have embarked upon a preliminary investigation of an array of pyrazolo-[1,5-*c*]quinazolinones.

Our primary aim is to develop an easy and efficacious synthetic strategy for the expeditious assembly of the pyrazolo-[1,5-*c*]quinazolinone framework. Simultaneously, we strive to identify pyrazolo-[1,5-*c*]quinazolinone derivatives that display potent antitumour activity and serve as valuable lead compounds targeting CDK9/2, thereby unveiling new opportunities for progress in the realm of anticancer drug discovery. Meanwhile, the further chemical modification and biological exploration of these compounds are underway in our laboratory.

## 2. Results and Discussion

### 2.1. Chemistry Efforts of the Synthesised Compounds

#### 2.1.1. Design and Optimisation of Synthesis

**Scheme 1:**
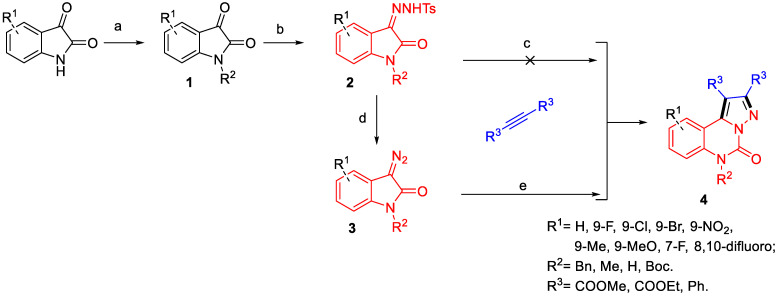
Design of synthesis route. (a). R^2^Br, K_2_CO_3_, DMF, 100 °C; (b). 4-methylbenzenesulfonhydrazide, 100 °C; (c) corresponding alkyne, K_2_CO_3_, 1,4-dioxane (for details, see Table 1, entries 1–4); (d) NaOH, H_2_O, 60 °C; (e) corresponding alkyne, toluene, 85 °C.

The development of efficient strategies for the synthesis of diverse and complex heterocyclic compounds has been a longstanding goal in synthetic organic chemistry due to their widespread applications across various scientific disciplines. While several approaches for the synthesis of the pyrazolo-[1,5-*c*]quinazolinone scaffold have been reported, many are limited by their reliance on metal catalysts, low atom economy, or cumbersome isolation procedures [30,31,32,33]. Inspired by the diazo compounds that have emerged as valuable synthetic platforms for the total synthesis of bioactive natural products [34,35], the *N*-Bn-protected isatin-derived tosylhydrazone was designed for the synthesis strategy of pyrazolo-[1,5-*c*]quinazolinones. The synthesis route in Scheme 1 shows that indole derivatives, which are inexpensive and readily available, were reacted with benzyl bromide and 4-methylbenzenesulfonhydrazide to yield the N-Bn-protected isatin-derived tosylhydrazone intermediates **2**. Unfortunately, no desired products were obtained when intermediates **2** reacted with dimethyl but-2-ynedioate using different Lewis base catalysts via an anticipated one-pot method of tandem reaction (Table 1, entries 1–4), although the reactions were processed under an argon atmosphere and the dry solvent conditions.

Despite initial setbacks, further investigation was conducted to better understand this reaction. The purified diazo isatin **3a** was isolated from the intermediate **2a** under NaOH conditions. It was then reacted with dimethyl but-2-ynedioate under heated conditions to successfully produce the desired product **4a**. To enhance the production of the target compound, we commenced our investigation with the model reaction of diazo isatin **3a** with dimethyl but-2-ynedioate (**5a**). As illustrated in Table 1, the high reactivity of product **4a** was observed after a series of chemistry attempts under different conditions. Interestingly, the reactions were tolerated under aqueous solvent, open-air, or acidic solvent conditions (entries 5–8). The influence of various solvents and temperature conditions were examined to further improve the yield of the reaction (entries 9–17). Among the solvents tested, most non-polar solvents were found to be suitable for the reaction particularly toluene and tetrahydrofuran. Based on these attempts and observations, optimal reaction conditions were established as being carried out in toluene at 85 °C without treatment with an anhydrous solvent or protection by inert gas. The structure of product **4a** was established by spectroscopic analysis and mass spectrometry.

#### 2.1.2. Scope of the Reaction

**Figure 2:**
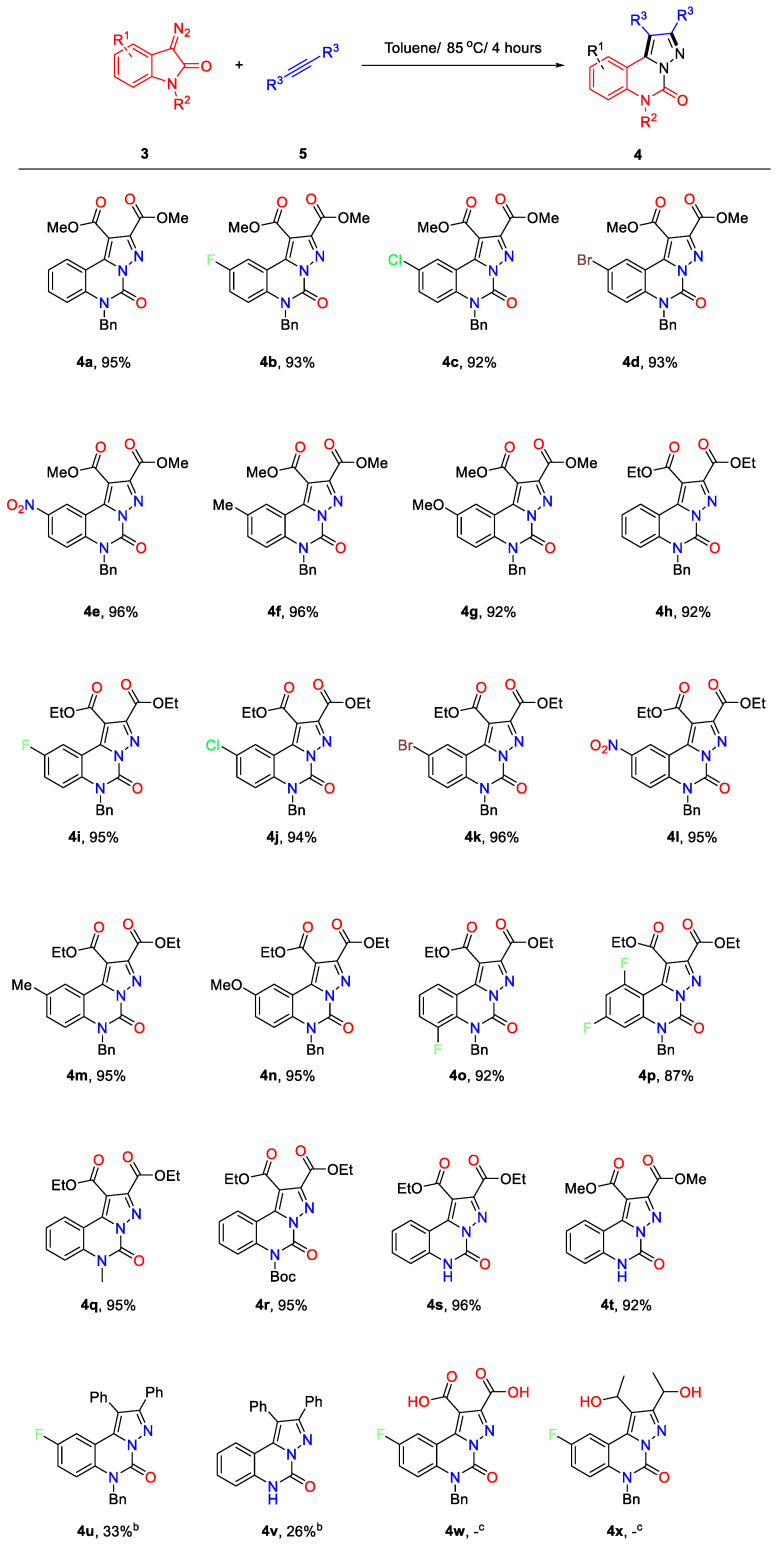
Chemical structures and synthesis yields of pyrazolo-[1,5-*c*]quinazolinone derivatives. Reaction conditions: the reactions were performed in a round-bottomed flask on a 0.25 mmol scale of **3** and 0.3 mmol scale of **5** in toluene (3 mL) at 85 °C for 4–6 h. ^b^ Yield of 140 °C/72 h. ^c^ The desired product was not observed.

To explore the scope of the reaction and further obtain diverse pyrazolo-[1,5-*c*]quinazolinone derivatives for the study of pharmacological activity, several different kinds of the diazo isatins were prepared and applied to synthesise the corresponding derivatives of esterified pyrazolo-[1,5-*c*]quinazolinones with the optimised conditions (Figure 2). Gratifyingly, the electronic property of the substituents on the benzene rings did not have significant impact on the reaction yields. The cyclisation reaction worked efficiently to afford the products **4a**–**4g** in excellent yields (over than 90% yields) whether the substituents were electron-withdrawing or electron-donating groups. Not surprisingly, the ethyl-esterified products **4h**–**4p** were synthesised in good to excellent yields when the raw material was replaced with diethyl but-2-ynedioate, which suggested that the reactions tolerated different electronic properties and the position of their benzene rings.

The presence of the highly reactive NH bond is responsible for the formation of the complex mixture, however, the NH position of the isatin substrates without any *N*-protecting groups did not inhibited the formation of the product **4s**/**4t** with the optimised conditions. The reactions of isatins with *N*-electron-withdrawing group (**4r**) were inferior to those of isatins with *N*-electron-donating groups (**4q**/**4h**); it appears that the electronic nature of the *N*-protecting groups had a slight impact on the reaction yields.

Although the products **4u** and **4v** could be observed weakly by this synthesis method, the reaction inefficiently worked after increasing the reaction temperature to 140 °C and extending the reaction time to 48 h. In view of the medium drug-like properties predicted by the derivative **4v** (see Table 5, violation of Lipinski rules), we have not prepared more of these derivatives. Regrettably, the expected products **4w** or **4x** were not observed while but-2-ynedioic acid or hex-3-yne-2,5-diol was used as the reactant instead of dimethyl but-2-ynedioate (**5a**). This novel method took advantage of high atom economy, metal-free process, and simple operation. Most of the products (**4a**–**4t**) were isolated by simple recrystallisation and filtration, which did not require any column chromatography technique for purification.

#### 2.1.3. Scale-Up and Green Chemistry Applications

**Scheme 2:**
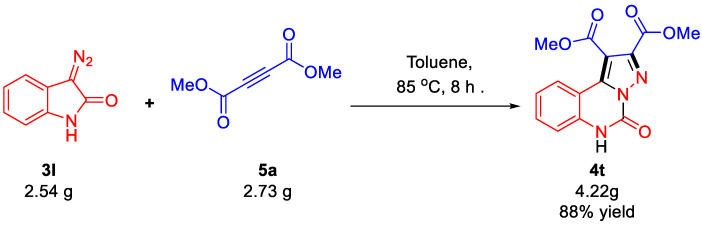
Gram-scale synthesis of **4t**. Reaction conditions: the reactions were performed in a round-bottomed flask on a 16.0 mmol scale of **3l** and 19.2 mmol scale of **5a** in toluene (30 mL) at 85 °C for 8 h.

Impressively, the formation of the products **4s** and **4t** was not prevented by the presence of the highly reactive NH bond of the starting materials **3l**. To demonstrate the reliability and practicality of the present synthetic methodology, a gram-scalable preparation was investigated using starting materials **3l** (2.54 g, 16.0 mmol) and **5a** (2.73 g, 19.2 mmol) to give the corresponding product **4t** in 88% yield (4.22 g, Scheme 2). This high yield confirms the practicality of this approach for large-scale synthesis.

In view of the fact that the reactions were tolerated under the conditions of aqueous and open-air atmosphere (Table 1, entry 6), we attempted the reaction in H_2_O at 85 °C to make the reaction much greener and to avoid the toluene solvent. It is regrettable that the reaction was not efficient at yielding the product **4t** within a short time, due to insolubility of the starting materials (Table 2, entry 1). Attempts were made to improve the solubility of the starting materials by performing the reaction in the various of mixed solvents. To our delight, the reaction proceeded, and an excellent improvement in the yield was observed in a H_2_O/THF (*v*/*v* = 2:1) mixture as solvent (Table 2, entry 2–4). A similar conclusion can be observed when the reactions of product **4k** and **4o** are used with a H_2_O/THF (*v*/*v* = 2:1) mixture as solvent (Table S1). This reaction provided the advantage of gram-scale synthesis and was environment friendly, indicating the broad application of the pharmaceutical industry.

#### 2.1.4. Plausible Mechanism of the Reaction

**Scheme 3:**
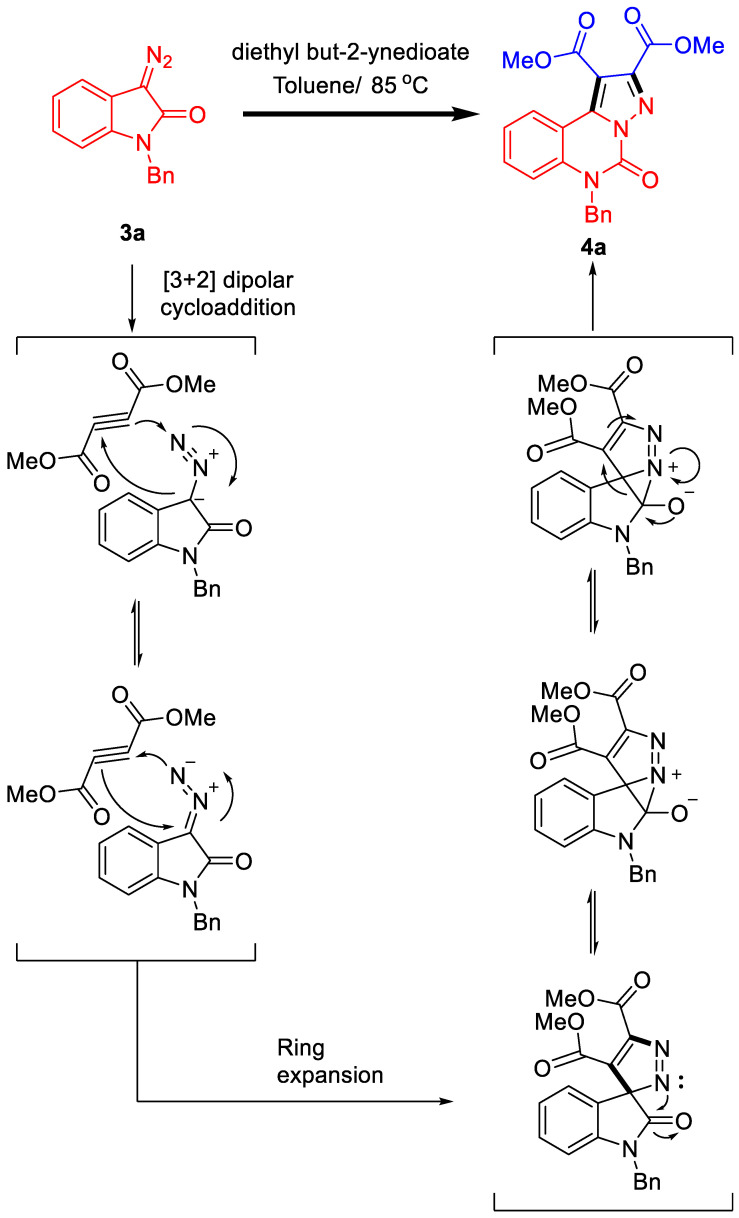
Plausible reaction mechanism.

On the basis of the experimental results and previous reports on bis(diazo)indolin-2-one chemistry, we proposed a plausible mechanism to rationalise the reaction pathways for the formation of **4a** (Scheme 3). A [3 + 2] dipolar cycloaddition of diethyl but-2-ynedioate and 3-diazoindolin-2-one proceeded to generate the intermediate spiro[indoline-3,3’-pyrazol]-2-one, which underwent acyl migration and rearrangement of ring expansion to form thermodynamically stable pyrazolo-[1,5-*c*]quinazolin-5(6*H*)-one under thermal conditions.

### 2.2. Biological Studies

#### 2.2.1. In Vitro Cytotoxicity Evaluation and Structure–Activity Relationship (SAR)

Previously, the widespread presence of quinazolinone moieties in antineoplastic drugs reflected their versatility and effectiveness in the treatment of cancer [3,4,5,7]. Therefore, the compounds of pyrazolo-[1,5-*c*]quinazolinones were selected and evaluated for in vitro antiproliferative activity against A549, MDA-MB-231, U-87, and HepG2 using the standard CCK8 assay, and 5-fluorouracil and abemaciclib (Abe) were used as positive controls.

**Figure 3:**
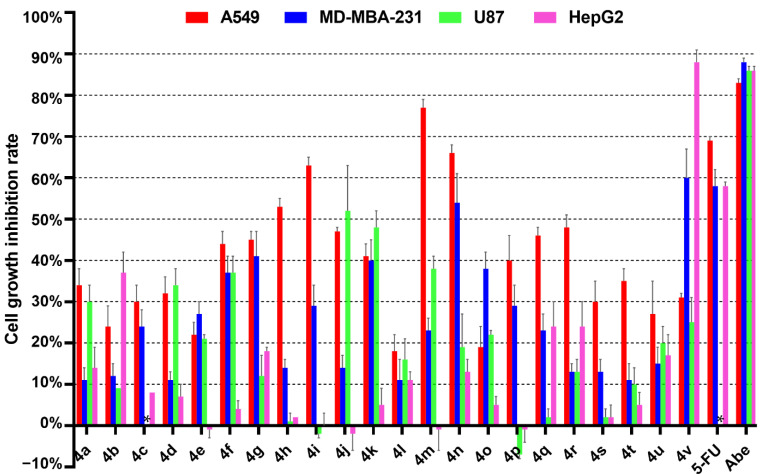
Cell growth inhibition rate of the pyrazolo-[1,5-*c*]quinazolinone derivatives on A549 cells, MDA-MB-231 cells, U87 cells, and HepG2 cells (30 μM).Cells were treated with compounds at the concentration of 30 μM for 48 h, the data represent mean ± SD (n = 3). 5-Fluorouracil (5-Fu) and abemaciclib (Abe) were used as positive controls at a concentration of 30 μM. * Not determined.

**Figure 4:**
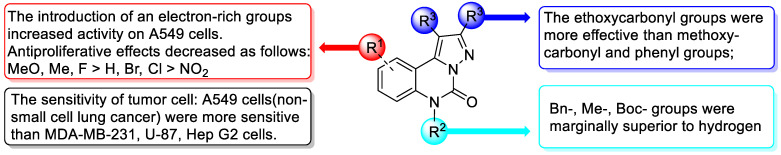
Structure–activity relationship (SAR) of a series of pyrazolo-[1,5-*c*]quinazolinone derivatives.

As demonstrated in Figure 3 and Table 3 and Table S2, A549 cells exhibit a higher sensitivity to the majority of compounds at a concentration of 30 μM compared to MDA-MB-231 cells, U-87 cells, or HepG2 cells. Further evaluation of the half-maximal inhibitory concentration (IC_50_) of the compounds was conducted on A549 and MDA-MB-231 cells. For A549 cells, compounds **4a**–**4g** (R^3^ = COOEt), which contain ethoxycarbonyl groups at the C1 and C2 positions of quinazolino, generally exhibit superior antiproliferative activity compared to compounds **4h**–**4s** (R^3^ = COOMe). Further analysis reveals that when R^1^ position of quinazolino is 9-methyl (**4m**) or 9-methoxy (**4n**), their antiproliferative activity is superior to H or 9-F/ 9-Cl/ 9-Br (**4h**–**4k**). The compound **4l** (R^1^ = 9-NO_2_) has little antitumour activity, indicating that the introduction of an electron-rich group on the benzene ring of quinazolino is significantly beneficial for improving cellular activity. When the R^2^ position of quinazolino is tert-butoxycarbonyl (**4r**), benzyl (**4h**), or methyl (**4q**), cellular activity is marginally superior to hydrogen (**4s**). However, when comparing benzyl substitution (**4a**) at R^2^ position with hydrogen substitution (**4t**), no significant improvement in cellular activity is observed. Additionally, it is believed that the tert-butoxycarbonyl group may have drawbacks such as a rapid metabolic rate during drug metabolism. Therefore, the R^2^ position may not be a suitable structural site for optimising activity. Importantly, compounds **4i**, **4m,** and **4n** (IC_50_ = 17.0, 14.2, and 18.1 μM, respectively) exhibit relatively potent antitumour activities compared with 5-fluorouracil (IC_50_ = 21.2 μM) against A549 cells. The inhibitory activities of compounds **4n** and **4v** on MDA-MB-231 cells were found to be comparable to the positive control drug, 5-fluorouracil. For U87 and HepG2 cells, only compound **4j**/**4k** (for U87 cells) and **4v** (for HepG2 cells) exhibit an inhibition rate greater than 40%, while the rest of the compounds were found to be insensitive. In summary, the structure-activity relationships of the synthesized derivatives are depicted in Figure 4. These results suggest that the pyrazolo-[1,5-*c*]quinazolinone framework has the potential to serve as a new template for the design of novel anticancer molecules.

#### 2.2.2. In Silico Studies of Biological Target and Molecular Modelling

**Figure 5:**
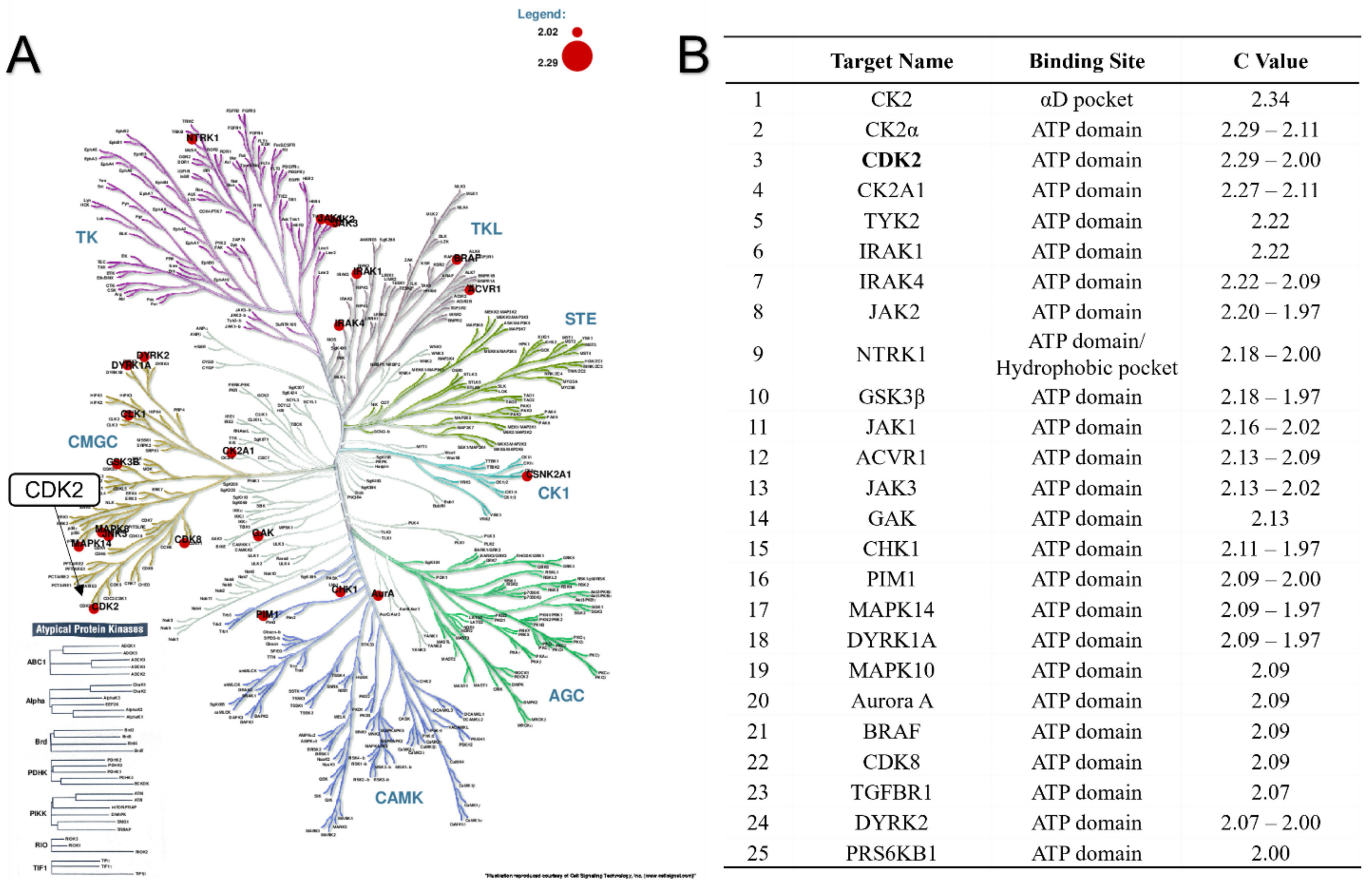
(**A**) The results of kinases target profiling were performed as the picture of phylogenetic tree; the red circle code means the predicted kinase targets. (**B**) The detailed data were ranked by their C value, with a higher C value indicating greater reliability of the calculated results.

Recent advances in deep-learning algorithms have made the accessible profiling of small molecules potential drug targets through in silico modelling approaches possible. The platform of ProfKin based on a kinase–ligand-focused database (KinLigDB) has been exploited as a multifunctional website for structure-based kinase target profiling [37]. The website (http://www.lilab-ecust.cn/profkin, accessed on 22 December 2022.) can be used to predict the possible binding modes, which can be helpful for target prediction and mechanistic study. To better understand the potential drug targets for which the compound exhibits antitumour effects, the assay of ProfKin was used to estimate the most probable kinases targets and binding conformations of compound **4t**. The results were displayed as the phylogenetic tree picture (Figure 5, the detailed data are provided in the Supplementary Materials). In short, compound **4t** can be used to identify probable drug targets through the inspection of top-ranked kinases, including CDK2, CK2, TYK, IRAK, JAK, NTRK, GSK3, CHK, PIM, MAPK, Aurora, RAF, and DYRK, among others.

**Figure 6:**
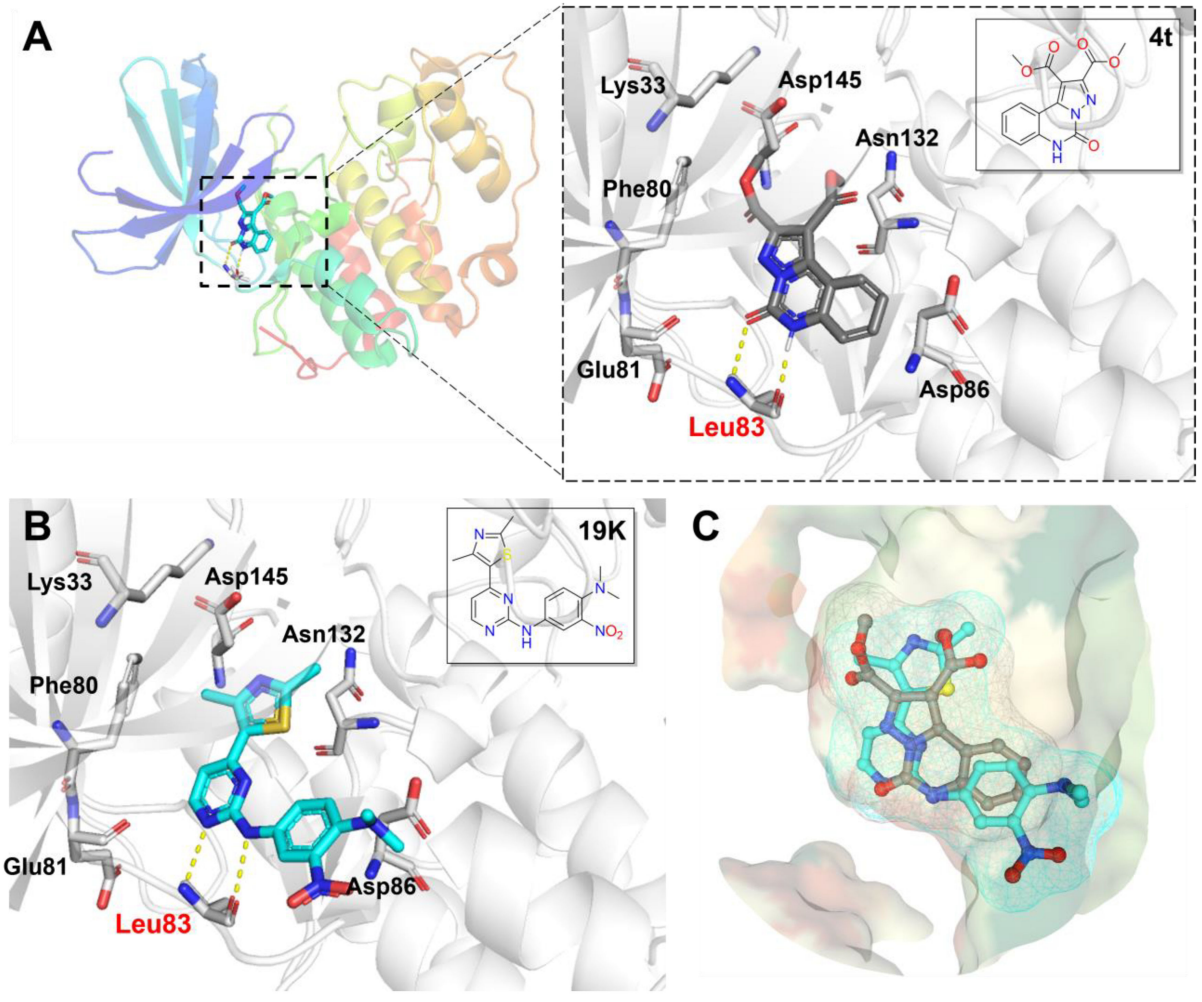
Binding mode analysis of compound **4t** within CDK2 kinase (ribbon representation, PDB code: 3SW7). The docking images were generated by the PyMOL molecular graphics system. The key amino acids of CDK2 are labelled, and hydrogen bonds in all panels are depicted by yellow dotted lines. (**A**) The binding pose of compound **4t** (grey stick model) in the CDK2. (**B**) The binding pose of the ligand 19K (cyan stick model) in the CDK2. (**C**) Comparison of the binding poses of **4t** and the ligand 19K within CDK2. The electron density of studied compounds is surfaced by the mesh.

As one of cyclin-dependent kinase (CDK) family members, CDK2 is an important regulatory factor of various carcinogenic signalling pathways. The inhibition of CDK2 provides a potential therapeutic benefit against certain tumour cells [38,39]. We focus on analysing the predicted binding poses of compound **4t** to CDK2 for developing the drug use of the pyrazolo-[1,5-*c*]quinazolinones backbone. As shown in Figure 6A, compound **4t** is located in a narrow cleft of the ATP-binding site between the N-terminal and C-terminal lobes. The pyrazolo-[1,5-*c*]quinazolinone core forms two hydrogen bonds with the leucine at the eighty-third position (Leu83) of CDK2. The binding poses of the compound **4t** in the ATP pocket are similar to the thiazolylpyrimidine ligand 19K by the visual inspection of binding modes (Figure 6B,C), which predicts the rationality and feasibility of the analysis of the binding mode. The C8 and C9 positions of quinazolino are located in the entranceway of the ATP-binding pocket and extend into the solvent-accessible area. Other research in the literature has reported that chemical structural modifications in solvent-accessible areas improve cellular activity [21,40], which is similar to our previous finding that the introduction of different groups on the benzene ring at this region leads to significant changes in antitumour cell activity. Thus, the pyrazolo-[1,5-*c*]quinazolinone compounds have the potential to exhibit antitumour effects by targeting CDK2, and could be used as a starting skeleton for the development of CDK inhibitors via a structure-based drug design, for which further research is already underway.

#### 2.2.3. CDK2/7/9 Enzyme Activity Assay

Considering the high degree of homology and conservation among the amino acid sequences of various CDK family kinase subtypes reported in the literature [38,39], we conducted a preliminary exploration of the CDK2/7/9 enzyme activity of selected compounds **4h**, **4n,** and **4t** with different structural characteristics. The results (Table 4) demonstrate that the compounds do not exhibit strong inhibitory activity against CDK2 at a concentration of 1.0 μM, which may be due to their lack of efficacy at low concentrations. Surprisingly, the compounds exhibit superior inhibitory activity against CDK9 compared to CDK2 or CDK7. Half-maximal inhibitory concentration assays of CDK9 reveal that the compounds fall within the micromolar concentration range (**4t**: 4.7 μM; **4n**: 9.8 μM). Notably, the previous literature has reported that the CDK9 inhibitor can induce apoptosis and death in A549 cells [41,42]. Therefore, the ability of pyrazolo-[1,5-*c*]quinazolinone derivatives to inhibit CDK9 kinase may contribute to the inhibition of A549 cell growth. This finding highlights the potential of targeting CDK9 as a therapeutic strategy for cancer treatment.

### 2.3. Drug-Likeness Studies In Silico

#### 2.3.1. Pharmacokinetic and Drug-Likeness Prediction

The development of new drugs requires not only good pharmacological activity, but also suitable physical and chemical properties and pharmacokinetic properties such as absorption, distribution, metabolism, and excretion (ADME), as well as acceptable toxicity effects. The calculation and prediction of the drug-like properties of these compounds in silico is valuable to improve the success of drug discovery.

**Figure 7:**
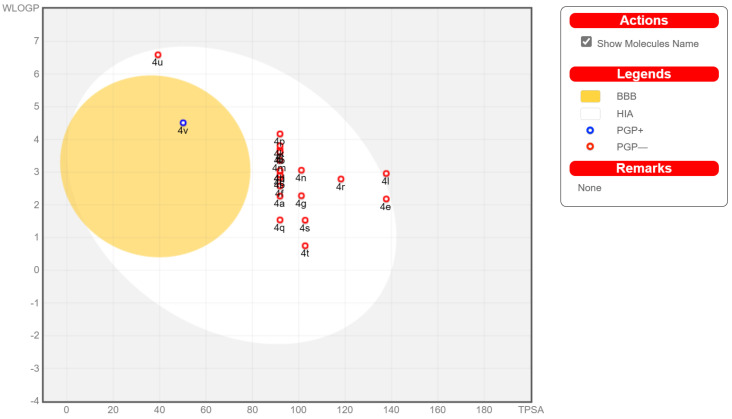
BOILED-EGG chart of the target molecules **4a**–**4v**. Results are presented as a 2D plot using the calculated TPSA and WLogP properties of the target molecules. The different points in the BOILED-EGG chart represent the target compounds **4a**–**4v**. The points located in the white ellipse represent compounds with high probability to be passively absorbed by the gastrointestinal tract, and located in the yellow yolk ellipse are for compounds with high probability to permeate through the BBB to access the CNS. The red point predicts no effluxing via P-glycoprotein (PGP−). If a predicted substrate of the P-glycoprotein (PGP+), the point colour is in blue.

In order to ensure that the compounds have good drug properties in the bioactivity study of this project, drug-likeness aspects prediction and pharmacokinetics of the studied pyrazolo-[1,5-*c*]quinazolinone derivatives were carried out in the available Swiss ADME [43]. The result (Figure 7) of BOILED-EGG chart indicates that most of the target compounds have a high probability of being passively absorbed by the gastrointestinal tract and do not permeate the blood–brain barrier (BBB). Additionally, these compounds may not be substrates for P-glycoprotein (P-gp) and, thus, eliminate the possibility of tumour cell line resistance through efflux mechanisms. However, compound **4v** was predicted to have a high probability of permeating through the BBB to access the central nervous system (CNS), while also having the ability to be a substrate for P-glycoprotein. Moreover, most of the compounds do not violate Lipinski rules and do not belong to PAINS compounds. The detailed drug-likeness parameters of the studied compounds are listed in Table 5. Most compounds are estimated to have negligible activity on cytochrome P450 isomers (e.g., CYP3A2 and CYP2D6) and are, therefore, considered to have no drug–drug interactions upon administration. Additionally, moderate bioavailability (score = 0.55) was predicted for the target molecules based on their compliance with five rule-based drug filters (Lipinski, Ghose, Veber, Egan, and Muegge rules).

#### 2.3.2. Toxicity Risk Assessment

The toxicity of chemicals should be known to avoid unwanted events. To anticipate the potential toxicity of compounds, the chemical structures of molecules were evaluated using the available OSIRIS property explorer to determine probable toxicity risks such as mutagenicity, carcinogenicity, tumorigenicity, and teratogenicity [44]. The OSIRIS property explorer calculates these risks based on functional group similarities between the investigated compound and those in its database with known in vitro and in vivo studies. The results (Table 6) indicate that the majority of the studied compounds are deemed safe and exhibit no or low toxicity concerning mutagenicity, tumorigenicity, and irritant effects. However, compound **4r** may pose a high risk of tumorigenicity and irritant effects, and compounds **4u** and **4v** may have a high risk to the reproductive system, while others exhibit moderate toxic effects on it.

The acute toxicity prediction of pyrazolo-[1,5-*c*]quinazolinone derivatives in this study were performed using the ProTox-II webserver [45,46,47]. Most of the compounds have similar LD_50_ values in the range of 564–1140 mg/kg and are predicted to belong to a medium toxicity class (Table 6). This toxicity level was categorised as toxicity class number 4 from 6 classes and turned to moderate toxicity, especially for oral consumption. The detailed toxicity profiles calculated with the help of ProTox-II are shown in Supplementary Information (Figure S2). In summary, further research into the optimisation and pharmacological investigation of these compounds holds significant potential and is highly encouraged.

## 3. Conclusions

In conclusion, our study presents the first report on the utilisation of pyrazolo-[1,5-*c*]quinazolinone derivatives for antitumour pharmacological applications. We have developed an efficient and straightforward methodology to rapidly access functionalised pyrazolo-[1,5-c]quinazolinones via a [3 + 2] dipolar cycloaddition and regioselective ring expansion process. This reaction strategy offers several advantages including high yield, high atom economy, metal-free process, simple operation, gram-scale synthesis, and environmental friendliness. In a series of in vitro studies, we found that some of the compounds demonstrated antitumour activities and inhibitory activity against CDK9/2. In silico studies indicate that the majority of the synthesised derivatives exhibit good drug-like properties and safety profiles. Our study not only provides a convenient and efficient synthetic method but also provides helpful structure-based guided drug design for further development of novel cyclin-dependent kinase (CDK) inhibitors. Further research into the application of these types of compounds in medicine would be valuable and meaningful.

## 4. Experimental Section

### 4.1. General Methods and Materials

All reactions were monitored by analytical thin-layer chromatography (TLC), which was visualised by ultraviolet light (254 nm or 356 nM). All solvents were obtained from commercial sources and were purified according to standard procedures. Purification of the products was accomplished by recrystallisation or flash chromatography using silica gel (100–200 mesh). Melting points were determined by WRR-Y drug melting point apparatus (Shanghai Yidian Physical and Optical Instruments Co., Ltd., Shanghai, China.). All NMR spectra were recorded with JEOL JNM-ECZ400S/L1 spectrometer at 400 MHz in DMSO or CDCl_3_: chemical shifts (*δ*) are given in ppm, coupling constants (*J*) in Hz, and the following abbreviations are used to indicate the multiplicity in NMR spectra: s (singlet); d (doublet); t (triplet); q (quartet); m (multiplet). The solvent signals were used as references (residual DMSO in DMSO-*d_6_*: *δ*_H_ = 2.50 ppm, *δ*_c_ = 39.5 ppm; CHCl_3_ in CDCl_3_: *δ*_H_ = 7.26 ppm, *δ*_c_ = 77.0 ppm). Mass spectra were obtained on the Agilent 1100 LC/MSD mass spectrometer (Agilent, Santa Clara, CA, USA) and Q-tofmicro MS (micromass company). High resolution mass spectrometry (HRMS) was recorded on TOF primer for ESI^+^. The purity of biologically evaluated compounds was >95% as determined by HPLC analysis (Waters e2695, C18 column (50 × 2.00 mm), with CH_3_OH and H_2_O as the mobile phase, monitored by UV absorption at 231 nm).

#### 4.1.1. General Procedure for the Synthesis of Isatin-Derived Tosylhydrazone **2** and the Diazo Isatin **3**

The isatin-derived tosylhydrazone **2** and the diazo isatin **3** are prepared according to a known procedure starting from substituted isatins [48].

The diazo isatin **3** are synthesised by compounds **2** with a sodium hydroxide aqueous solution condition. The corresponding isatin-derived tosylhydrazone **2** (10 mmol) and NaOH (1.0 g, 25 mmol) were suspended in water (30 mL), and the suspension was stirred at 50 °C for 2 h until the completion of the reaction as monitored by TLC. The reaction mixture was cooled down, then the residue was collected by the filtration funnel and was washed with water (10 mL × 3). After air-drying, the diazo product **3** appears as a red or orange solid.

1-benzyl-3-diazoindolin-2-one (**3a**). Orange solid. ^1^H NMR (400 MHz, chloroform-*d*) δ = 7.48 (dt, J = 9.2, 4.7, 1H), 7.42–7.24 (m, 5H), 7.16–7.08 (m, 1H), 7.08–6.98 (m, 2H), 5.00 (s, 2H). ^13^C NMR (100 MHz, chloroform-*d*) δ 167.2, 136.3, 133.9, 129.1, 128.0, 127.6, 125.7, 122.5, 118.6, 117.1, 109.9, 44.6.

#### 4.1.2. General Procedure for the Synthesis of **4**

To a 25 mL round-bottomed flask was added the diazo isatin **3** (0.25 mmol), alkyne **5** (0.30 mmol), and the toluene (3 mL). The resulting mixture was heated at 85 °C in air for a period of time (usually 4–6 h). After completion of the reaction as monitored by TLC, the mixture was cooled to room temperature. The solvent was evaporated under reduced pressure and the residue was purified by recrystallisation (ethyl acetate/n-hexane) or chromatography (ethyl acetate/petroleum ether) on silica gel to afford products **4**.

#### 4.1.3. Characterisation of Products **4a**–**4v**

Dimethyl 6-benzyl-5-oxo-5,6-dihydropyrazolo[1,5-*c*]quinazoline-1,2-dicarboxylate (**4a**). Pale pink solid, 95% yield, mp: 224–225 °C. ^1^H NMR (400 MHz, chloroform-*d*) *δ* 8.7 (d, *J* = 8.0 Hz, 1H), 7.5 (t, *J* = 8.0 Hz, 1H), 7.3 (d, 1H), 7.3–7.3 (m, 3H), 7.3–7.2 (m, 3H), 5.6 (s, 2H), 4.0 (s, 6H). ^13^C NMR (100 MHz, chloroform-*d*) *δ* 163.5, 162.2, 148.1, 145.3, 140.0, 135.7, 134.8, 132.3, 129.2(2C), 128.0, 126.9, 126.7(2C), 124.3, 115.9, 112.6, 110.53, 53.0(2C), 48.2. HRMS (ESI) calcd. for C_21_H_18_N_3_O_5_ (M+H)^+^: 392.1241, found 392.1243. HPLC retention time 3.49 min, purity 95.7%.

Dimethyl 6-benzyl-9-fluoro-5-oxo-5,6-dihydropyrazolo[1,5-*c*]quinazoline-1,2-dicarboxylate (**4b**). Pale pink solid, 93% yield, mp: 186–187 °C. ^1^H NMR (400 MHz, chloroform-*d*) *δ* 8.6 (d, *J* = 9.2 Hz, 1H), 7.3 (t, 1H), 7.3 (d, *J* = 7.4 Hz, 2H), 7.3–7.2 (m, 3H), 7.2 (d, *J* = 8.3 Hz, 1H), 5.6 (s, 2H), 4.0 (s, 3H), 4.0 (s, 3H). ^13^C NMR (100 MHz, chloroform-*d*) *δ* 163.0, 162.2, 148.7, 144.9, 138.9, 135.1, 134.7, 134.4, 129.6, 129.3(2C), 128.2, 126.6(2C), 117.5, 117.4, 114.2, 110.81, 53.1(2C), 48.4. HRMS (ESI) calcd. for C_21_H_17_FN_3_O_5_ (M+H)^+^: 410.1147, found 410.1157. HPLC retention time 3.73 min, purity 99.5%.

6-benzyl-9-chloro-1,2-bis(methoxycarbonyl)-5-oxo-5,6-dihydropyrazolo[1,5-*c*]quinazolin-3-ium (**4c**)**.** Light yellow solid, 92% yield, mp: 161–162 °C. ^1^H NMR (400 MHz, chloroform-*d*) *δ* 8.8 (d, *J* = 2.4 Hz, 1H), 7.4 (dd, *J* = 9.0, 2.4 Hz, 1H), 7.3–7.3 (m, 2H), 7.3–7.2 (m, 4H), 5.6 (s, 2H), 4.0 (s, 3H), 4.0 (s, 3H). ^13^C NMR (100 MHz, chloroform-*d*) *δ* 163.0, 162.1, 148.6, 144.9, 139.0, 134.4, 134.3, 132.3, 129.9, 129.2(2C), 128.2, 126.6(2C), 126.5, 117.4, 113.7, 110.7, 53.1(2C), 48.4. HRMS (ESI) calcd. for C_21_H_17_ClN_3_O_5_ (M+H)^+^: 426.0851, found 426.0849. HPLC retention time 4.21 min, purity 99.6%.

Dimethyl 6-benzyl-9-bromo-5-oxo-5,6-dihydropyrazolo[1,5-*c*]quinazoline-1,2-dicarboxylate (**4d**). Pale pink solid, 93% yield, mp: 196–198 °C. ^1^H NMR (400 MHz, chloroform-*d*) *δ* 8.9 (s, 1H), 7.6 (d, *J* = 9.1 Hz, 1H), 7.3 (d, *J* = 7.3 Hz, 2H), 7.3–7.2 (m, 3H), 7.2 (d, *J* = 8.8 Hz, 1H), 5.6 (s, 2H), 4.0 (s, 3H), 4.0 (s, 3H). ^13^C NMR (100 MHz, chloroform-*d*) *δ* 163.0, 162.2, 148.7, 144.9, 138.9, 135.1, 134.7, 134.4, 129.6, 129.3(2C), 128.2, 126.6(2C), 117.5, 117.4, 114.2, 110.81 53.1(2C), 48.4. HRMS (ESI) calcd. for C_21_H_17_BrN_3_O_5_ (M+H)^+^: 470.0346, found 470.0353. HPLC retention time 4.16 min, purity 98.4%.

Dimethyl 6-benzyl-9-nitro-5-oxo-5,6-dihydropyrazolo[1,5-*c*]quinazoline-1,2-dicarboxylate (**4e**). Light yellow solid, 96% yield, mp: 226–228 °C. ^1^H NMR (400 MHz, chloroform-*d*) *δ* 9.9 (d, *J*= 2.5 Hz, 1H), 8.3 (dd, *J* = 9.4 Hz, 2.5 Hz, 1H), 7.4 (d, *J* = 9.3 Hz, 1H), 7.4–7.3 (m, 3H), 7.3 (dd, *J* = 7.3 Hz, 1.3 Hz, 2H), 5.7 (s, 2H), 4.0 (s, 3H), 4.0 (s, 3H). ^13^C NMR (100 MHz, chloroform-*d*) *δ* 162.5, 161.9, 149.3, 144.7, 143.5, 139.9, 138.9, 133.8, 129.5(2C), 128.5, 126.8, 126.6(2C), 123.4, 116.8, 112.9, 111.41, 53.3, 53.3, 48.9. HRMS (ESI) calcd. for C_21_H_17_N_4_O_7_ (M+H)^+^: 437.1092, found 437.1093. HPLC retention time 3.72 min, purity 97.0%.

Dimethyl 6-benzyl-9-methyl-5-oxo-5,6-dihydropyrazolo[1,5-*c*]quinazoline-1,2-dicarboxylate (**4f**). Pale pink solid, 96% yield, mp: 165–167 °C. ^1^H NMR (400 MHz, chloroform-*d*) *δ* 8.4 (s, 1H), 7.3 (d, *J* = 7.1 Hz, 1H), 7.3 (d, 2H), 7.2–7.2 (m, 3H), 7.1 (d, *J* = 9.8 Hz, 1H), 5.5 (s, 2H), 4.0 (s, 3H), 4.0 (s, 3H), 2.4 (s, 3H). ^13^C NMR (100 MHz, chloroform-*d*) *δ* 163.5, 162.3, 148.0, 145.2, 140.0, 134.9, 134.2, 133.6, 133.3, 129.1(2C), 128.0, 126.7(2C), 126.6, 115.7, 112.4, 110.29, 53.0(2C), 48.1, 21.0. HRMS (ESI) calcd. for C_22_H_20_N_3_O_5_ (M+H)^+^: 406.1397, found 406.1390. HPLC retention time 3.97 min, purity 98.6%.

Dimethyl 6-benzyl-9-methoxy-5-oxo-5,6-dihydropyrazolo[1,5-*c*]quinazoline-1,2-dicarboxylate (**4g**). Grey–white solid, 92% yield, mp: 172–174 °C. ^1^H NMR (400 MHz, chloroform-*d*) *δ* 8.4 (s, 1H), 7.3 (d, *J* = 7.5 Hz, 2H), 7.2–7.2 (m, 3H), 7.2 (d, *J* = 8.3 Hz, 1H), 7.0 (d, *J* = 9.0 Hz, 1H), 5.5 (s, 2H), 4.0 (s, 3H), 3.9 (s, 3H), 3.8 (s, 3H). ^13^C NMR (100 MHz, chloroform-*d*) *δ* 163.3, 162.5, 155.9, 148.6, 145.1, 140.2, 135.0, 129.7, 129.1(2C), 128.0, 126.7(2C), 120.5, 117.1, 113.3, 110.0, 109.5, 55.8, 53.0, 52.9, 48.2. HRMS (ESI) calcd. for C_22_H_20_N_3_O_6_ (M+H)^+^: 422.1347, found 422.1352. HPLC retention time 3.80 min, purity 98.8%.

Diethyl 6-benzyl-5-oxo-5,6-dihydropyrazolo[1,5-*c*]quinazoline-1,2-dicarboxylate (**4h**). Yellow solid, 92% yield, mp: 168–169 °C. ^1^H NMR (400 MHz, chloroform-*d*) *δ* 8.8 (d, *J* = 8.0 Hz, 1H), 7.5 (t, *J* = 7.2 Hz, 1H), 7.3 (d, *J* = 7.4 Hz, 1H), 7.3–7.3 (m, 3H), 7.3–7.3 (m, 3H), 5.6 (s, 2H), 4.5 (qd, *J* = 7.1, 3.9 Hz, 4H), 1.4 (dt, *J* = 11.3, 7.1 Hz, 6H). ^13^C NMR (100 MHz, chloroform-*d*) *δ* 163.0, 162.1, 145.4, 140.0, 135.7, 134.9, 132.2, 129.2(2C), 128.0, 127.1, 126.7(2C), 124.3, 115.8, 112.8, 62.4, 62.1, 48.2, 14.3, 14.1. HRMS (ESI) calcd. for C_23_H_22_N_3_O_5_ (M+H)^+^: 420.1554, found 420.1557. HPLC retention time 3.05 min, purity 97.8%.

Diethyl 6-benzyl-9-fluoro-5-oxo-5,6-dihydropyrazolo[1,5-*c*]quinazoline-1,2-dicarboxylate (**4i**). White–pink solid, 95% yield, mp: 137–139 °C. ^1^H NMR (400 MHz, chloroform-*d*) *δ* 8.6 (dd, *J* = 9.5, 2.9 Hz, 1H), 7.2 (d, 1H), 7.2–7.2 (m, 1H), 7.2–7.1 (m, 3H), 7.1–7.1 (m, 2H), 5.5 (s, 2H), 4.4 (dq, *J* = 9.9, 7.1 Hz, 4H), 1.3 (dt, *J* = 9.9, 7.1 Hz, 6H). ^13^C NMR (100 MHz, chloroform-*d*) *δ* 162.3, 162.0, 149.3, 144.9, 139.24, 134.6, 132.1, 129.1(2C), 128.0, 126.6(2C), 120.0, 119.7, 117.8, 117.7, 113.4, 110.5, 62.4, 62.2, 48.3, 14.2, 13.9. HRMS (ESI) calcd. for C_23_H_21_FN_3_O_5_ (M+H)^+^: 438.1460, found 438.1463. HPLC retention time 4.42. min, purity 98.4%.

Diethyl 6-benzyl-9-chloro-5-oxo-5,6-dihydropyrazolo[1,5-*c*]quinazoline-1,2-dicarboxylate (**4j**). White solid, 94% yield, mp: 165–166 °C. ^1^H NMR (400 MHz, chloroform-*d*) *δ* 8.8 (s, 1H), 7.3 (d, *J* = 9.2 Hz, 1H), 7.2 (d, *J* = 7.8 Hz, 1H), 7.2 (d, 2H), 7.2 (t, 3H), 5.5 (s, 2H), 4.4 (dq, *J* = 7.4, 3.7 Hz, 4H), 1.4 (q, *J* = 6.6, 6.1 Hz, 6H). ^13^C NMR (100 MHz, chloroform-*d*) *δ* 162.3, 162.0, 149.3, 145.0, 138.9, 134.5, 134.2, 132.2, 129.8(2C), 129.2, 128.1, 126.6(3C), 117.3, 113.7, 110.7, 62.5, 62.3, 48.3, 14.2, 14.0. HRMS (ESI) calcd. for C_23_H_21_ClN_3_O_5_ (M+H)^+^: 454.1164, found 454.1176. HPLC retention time 4.59 min, purity 97.6%.

Diethyl 6-benzyl-9-bromo-5-oxo-5,6-dihydropyrazolo[1,5-*c*]quinazoline-1,2-dicarboxylate (**4k**). White solid, 96% yield, mp: 167–168 °C. ^1^H NMR (400 MHz, chloroform-*d*) *δ* 8.9 (s, 1H), 7.5 (d, *J* = 9.0 Hz, 1H), 7.2 (d, *J* = 6.8 Hz, 2H), 7.2–7.1 (m, 3H), 7.1 (d, *J* = 9.1 Hz, 1H), 5.5 (s, 2H), 4.4 (qd, *J* = 7.2, 3.4 Hz, 4H), 1.4 (td, *J* = 7.0, 3.5 Hz, 6H). ^13^C NMR (100 MHz, chloroform-*d*) *δ* 162.3, 161.9, 149.3, 144.9, 138.7, 135.0, 134.6, 134.5, 129.5, 129.2(2C), 128.1, 126.6(2C), 117.6, 117.2, 114.1, 110.7, 62.5, 62.3, 48.2, 14.2, 14.0. HRMS (ESI) calcd. for C_23_H_21_BrN_3_O_5_ (M+H)^+^: 498.0659, found 498.0657. HPLC retention time 4.76 min, purity 98.4%.

Diethyl 6-benzyl-9-nitro-5-oxo-5,6-dihydropyrazolo[1,5-*c*]quinazoline-1,2-dicarboxylate (**4l**). Light yellow solid, 95% yield, mp: 168–169 °C. ^1^H NMR (400 MHz, chloroform-*d*) *δ* 9.9 (s, 1H), 8.3 (d, *J* = 9.2 Hz, 1H), 7.4 (d, *J* = 9.5 Hz, 1H), 7.3–7.3 (m, 3H), 7.2 (d, *J* = 7.3 Hz, 2H), 5.6 (s, 2H), 4.5–4.4 (m, 4H), 1.4 (t, *J* = 7.2 Hz, 6H). ^13^C NMR (100 MHz, chloroform-*d*) *δ* 162.0, 161.7, 149.9, 144.8, 143.5, 139.9, 138.7, 133.8, 129.4(2C), 128.5, 126.7, 126.6(2C), 123.5, 116.8, 112.9, 111.5, 62.7, 62.6, 48.9, 14.2, 14.0. HRMS (ESI) calcd. for C_23_H_21_N_4_O_7_ (M+H)^+^: 465.1405, found 465.1412. HPLC retention time 4.44 min, purity 98.1%.

Diethyl 6-benzyl-9-methyl-5-oxo-5,6-dihydropyrazolo[1,5-*c*]quinazoline-1,2-dicarboxylate (**4m**). White solid, 95% yield, mp: 141–143 °C. ^1^H NMR (400 MHz, chloroform-*d*) *δ* 8.5 (s, 1H), 7.2 (d, *J* = 8.0 Hz, 1H), 7.2 (d, 2H), 7.2–7.1 (m, 3H), 7.1 (d, *J* = 8.8 Hz, 1H), 5.5 (s, 2H), 4.4 (q, *J* = 7.3 Hz, 4H), 2.3 (s, 3H), 1.4 (q, *J* = 6.4 Hz, 6H). ^13^C NMR (100 MHz, chloroform-*d*) *δ* 162.9, 162.1, 148.7, 145.3, 139.8, 135.0, 134.1, 133.4, 133.2, 129.0(2C), 127.9, 126.7(3C), 115.7, 112.4, 110.3, 62.3, 62.0, 47.9, 20.9, 14.2, 14.1. HRMS (ESI) calcd. for C_24_H_24_N_3_O_5_ (M+H)^+^: 434.1710, found 434.1716. HPLC retention time 4.69 min, purity 98.8%.

Diethyl 6-benzyl-9-methoxy-5-oxo-5,6-dihydropyrazolo[1,5-*c*]quinazoline-1,2-dicarboxylate (**4n**). White solid, 95% yield, mp: 116–117 °C. ^1^H NMR (400 MHz, chloroform-*d*) *δ* 8.4 (s, 1H), 7.2 (d, *J* = 7.6 Hz, 2H), 7.2–7.1 (m, 3H), 7.1 (d, *J* = 9.3 Hz, 1H), 6.9 (d, *J* = 9.3 Hz, 1H), 5.5 (s, 2H), 4.4 (dq, *J* = 19.4, 6.7 Hz, 4H), 3.8 (s, 3H), 1.4 (dt, *J* = 14.9, 7.0 Hz, 6H). ^13^C NMR (100 MHz, chloroform-*d*) *δ* 162.7, 162.3, 155.8, 149.2, 145.1, 140.1, 135.0, 129.5, 129.0(2C), 127.9, 126.7(2C), 120.3, 117.0, 113.3, 109.9, 109.5, 62.3, 62.0, 55.7, 48.0, 14.2, 14.0. HRMS (ESI) calcd. for C_24_H_24_N_3_O_6_ (M+H)^+^: 450.1660, found 450.1660. HPLC retention time 4.39 min, purity 98.5%.

Diethyl 6-benzyl-7-fluoro-5-oxo-5,6-dihydropyrazolo[1,5-*c*]quinazoline-1,2-dicarboxylate **(4o)**. White–pink solid, 92% yield, mp: 149–150 °C. ^1^H NMR (400 MHz, chloroform-*d*) *δ* 8.5 (d, *J* = 5.5 Hz, 1H), 7.2 (d, *J* = 2.2 Hz, 1H), 7.2–7.2 (m, 5H), 7.2 (d, *J* = 2.2 Hz, 1H), 5.7 (s, 2H), 4.5–4.3 (m, 4H), 1.4 (dt, *J* = 13.2, 7.4 Hz, 6H). ^13^C NMR (100 MHz, chloroform-*d*) *δ* 162.9, 161.7, 148.8, 145.5, 136.2, 128.7(2C), 127.7, 126.6(2C), 125.3, 125.2, 122.8, 122.8, 120.3, 120.0, 115.2, 111.3, 62.4, 62.3, 51.0, 14.2, 14.0. HRMS (ESI) calcd. for C_23_H_21_FN_3_O_5_ (M+H)^+^: 438.1460, found 438.1456. HPLC retention time 4.32 min, purity 97.0%.

Diethyl 6-benzyl-8,10-difluoro-5-oxo-5,6-dihydropyrazolo[1,5-*c*]quinazoline-1,2-dicarboxylate (**4p**). Light yellow solid, 87% yield, mp: 194–196 °C. ^1^H NMR (400 MHz, chloroform-*d*) *δ* 7.4 (d, *J* = 7.8 Hz, 1H), 7.3–7.3 (m, 2H), 7.3 (d, *J* = 3.7 Hz, 2H), 6.9 (d, *J* = 11.4 Hz, 1H), 6.8–6.8 (m, 1H), 5.5 (s, 2H), 4.5 (q, *J* = 7.3 Hz, 4H), 1.5–1.4 (m, 6H). ^13^C NMR (100 MHz, chloroform-*d*) *δ* 163.5, 160.6, 160.3, 157.79, 145.9, 145.0, 137.8, 134.0, 132.5, 129.3(2C), 128.4, 126.6(2C), 115.0, 100.5, 100.3, 99.9, 62.4, 62.3, 48.9, 14.2, 14.1. HRMS (ESI) calcd. for C_23_H_20_F_2_N_3_O_5_ (M+H)^+^: 456.1366, found 456.1363. HPLC retention time 4.09 min, purity 97.6%.

Diethyl 6-methyl-5-oxo-5,6-dihydropyrazolo[1,5-*c*]quinazoline-1,2-dicarboxylate (**4q**). Orange solid, 95% yield, mp: 117–119 °C. ^1^H NMR (400 MHz, chloroform-*d*) *δ* 8.8 (d, *J* = 8.2 Hz, 1H), 7.6 (t, *J* = 7.9 Hz, 1H), 7.4–7.3 (m, 2H), 4.5–4.4 (m, 4H), 3.8 (s, 3H), 1.4 (td, *J* = 8.2, 6.0 Hz, 6H). ^13^C NMR (100 MHz, chloroform-*d*) *δ* 162.9, 162.1, 148.7, 144.8, 139.8, 136.4, 132.3, 127.0, 124.2, 114.8, 112.5, 110.3, 62.3, 62.0, 31.8, 14.2, 14.0. HRMS (ESI) calcd. for C_17_H_18_N_3_O_5_ (M+H)^+^: 344.1241, found 344.1244. HPLC retention time 3.22 min, purity 97.0%.

6-(*tert*-butyl) 1,2-diethyl 5-oxopyrazolo[1,5-*c*]quinazoline-1,2,6(5*H*)-tricarboxylate (**4r**). Light yellow solid, 95% yield, mp: 181–182 °C. ^1^H NMR (400 MHz, chloroform-*d*) *δ* 8.8 (d, *J* = 8.2 Hz, 1H), 7.6 (t, *J* = 7.9 Hz, 1H), 7.4 (t, *J* = 7.7 Hz, 1H), 7.2 (d, *J* = 8.4 Hz, 1H), 4.4 (q, *J* = 7.2 Hz, 4H), 1.7 (s, 9H), 1.4 (q, *J* = 7.2 Hz, 6H). ^13^C NMR (100 MHz, chloroform-*d*) *δ* 162.8, 161.8, 149.1, 148.3, 142.22, 140.1, 133.3, 132.3, 127.0, 125.0, 114.6, 111.9, 111.25, 88.6, 62.4, 62.2, 27.6(3C), 14.2, 14.0. HRMS (ESI) calcd. for C_21_H_24_N_3_O_7_ (M-100+H)^+^: 330.1084, found 330.1087. HPLC retention time 2.28 min, purity 98.9%.

Diethyl 5-oxo-5,6-dihydropyrazolo[1,5-*c*]quinazoline-1,2-dicarboxylate (**4s**). White solid, 96% yield, mp: 184–186 °C. ^1^H NMR (400 MHz, DMSO-*d*_6_) *δ* 12.34 (s, 1H), 8.46 (dd, *J* = 8.1, 1.3 Hz, 1H), 7.61 (ddd, *J* = 8.4, 7.3, 1.4 Hz, 1H), 7.38 (d, *J* = 7.2 Hz, 1H), 7.32 (ddd, *J* = 8.3, 7.3, 1.2 Hz, 1H), 4.37 (qd, *J* = 7.1, 5.1 Hz, 4H), 1.32 (dt, *J* = 11.2, 7.1 Hz, 6H). ^13^C NMR (100 MHz, DMSO-*d*_6_) *δ* 163.1, 162.3, 147.2, 144.1, 141.1, 136.2, 132.5, 125.7, 123.9, 116.7, 111.7, 109.8, 62.4, 62.2, 14.5, 14.3. HRMS (ESI) calcd. for C_16_H_16_N_3_O_5_ (M+H)^+^: 330.1084, found 330.1089. HPLC retention time 4.23 min, purity 95.0%.

Dimethyl 5-oxo-5,6-dihydropyrazolo[1,5-*c*]quinazoline-1,2-dicarboxylate (**4t**). Yellow solid, 92% yield, mp: 248–250 °C. ^1^H NMR (400 MHz, DMSO-*d*_6_) *δ* 12.3 (s, 1H), 8.4 (d, *J* = 8.1 Hz, 1H), 7.6 (td, *J* = 7.8, 7.2, 1.3 Hz, 1H), 7.4 (d, *J* = 8.3 Hz, 1H), 7.3 (t, *J* = 7.7 Hz, 1H), 3.9 (s, 3H), 3.9 (s, 3H). ^13^C NMR (100 MHz, DMSO-*d*_6_) *δ* 163.6, 162.6, 146.7, 144.0, 141.1, 136.1, 132.5, 125.5, 123.9, 116.7, 111.6, 109.8, 53.5, 53.4. HRMS (ESI) calcd. for C_14_H_12_N_3_O_5_ (M+H)^+^: 302.0771, found 302.0774. HPLC retention time 3.26 min, purity 98.1%.

6-benzyl-9-fluoro-1,2-diphenylpyrazolo[1,5-*c*]quinazolin-5(6*H*)-one (**4u**). White–pink solid, 33% yield, mp: 232–233 °C. ^1^H NMR (400 MHz, chloroform-*d*) *δ* 7.6 (d, *J* = 7.1 Hz, 2H), 7.5–7.5 (m, 3H), 7.5–7.4 (m, 2H), 7.4–7.3 (m, 4H), 7.3–7.3 (m, 4H), 7.2 (dd, *J* = 8.9, 4.3 Hz, 1H), 7.0 (q, *J* = 9.0 Hz, 2H), 5.6 (s, 2H). ^13^C NMR (100 MHz, chloroform-*d*) *δ* 154.9, 146.1, 135.5, 132.2, 131.7, 131.6, 130.6(2C), 129.7(2C), 129.1(2C), 128.9, 128.8(2C), 128.3(2C), 127.9, 126.7(2C), 118.2, 117.5(2C), 117.4, 117.3, 110.3, 110.0, 48.1. HRMS (ESI) calcd. for C_29_H_21_FN_3_O (M+H)^+^: 446.1663, found 446.1660. HPLC retention time 5.07 min, purity 99.3%.

1,2-diphenylpyrazolo[1,5-*c*]quinazolin-5(6*H*)-one (**4v**)**.** Yellow solid, 26% yield, mp: 189–190 °C. ^1^H NMR (400 MHz, DMSO-*d*_6_) *δ* 12.0 (s, 1H), 7.6–7.5 (m, 3H), 7.5–7.4 (m, 5H), 7.3 (d, *J* = 8.3 Hz, 1H), 7.3 (d, *J* = 5.9 Hz, 3H), 7.1 (d, *J* = 8.1 Hz, 1H), 7.0 (t, *J* = 7.6 Hz, 1H). ^13^C NMR (100 MHz, DMSO-*d*_6_) *δ* 153.2, 144.9, 137.7, 135.3, 133.0, 132.4, 131.1(2C), 130.6, 130.0(2C), 129.2, 129.1, 128.9(2C), 128.4(2C), 123.3, 122.9, 117.1, 116.5, 113.1. HRMS (ESI) calcd. for C_22_H_16_N_3_O (M+H)^+^: 338.1288, found 338.1286. HPLC retention time 4.52 min, purity 98.4%.

### 4.2. Biology and In Silico Study Methods

The experimental details are presented in Supplementary Materials.

## Figures and Tables

**Table 1:** Optimisation of the conditions for the formation of **4a** ^a.^

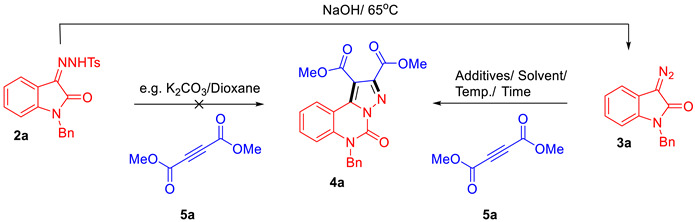						
Entry	2a/3a	Additives	Solvent	Temp. (^o^C)	Time (hour)	Yield (%)
1	2a	K_2_CO_3_ ^b^	Dioxane	105	24	- ^c^
2	2a	Cs_2_CO_3_ ^b^	Dioxane	105	24	- ^c^
3	2a	NaOH ^b^	Dioxane	105	24	- ^c^
4	2a	Bu_3_P ^d^	DCM	45	48	- ^c^
5	3a	-	Dioxane	105	4	83%
6 ^e^	3a	-	Dioxane	105	4	86%
7	3a	HCl ^f^	Dioxane	105	4	80%
8	3a	NaOH ^g^	Dioxane	95	4	Trace
9	3a	-	DME	90	4	56%
10	3a	-	DMF	65	12	Trace
11	3a	-	EtOH	85	2	51%
12	3a	-	CH_3_CN	85	12	Trace
13	3a	-	DCE	85	8	85%
14	3a	-	THF	65	6	88%
15 ^e^	3a	-	THF	45	8	86%
16	3a	-	Toluene	110	2	90%
17 ^e^	3a	-	Toluene	85	4	96%

^a^ Reaction conditions: the reactions were carried out in a round-bottomed flask on a 0.3 mmol scale of **5a** and 0.25 mmol scale of **2a** or **3a** in dry solvent (3 mL) under an argon atmosphere. Temp. = temperature. ^b^ Additives (1.2 equivalent). ^c^ The desired product was not observed. ^d^ Additives (0.3 equivalent). ^e^ The reaction was carried out under an open-air condition. ^f^ A total of 16 μL HCl was added to the reaction. ^g^ NaOH (2.0 equivalent) was added to the reaction.

**Table 2:** Green synthesis of **4t** ^a.^

Entry	Solvent (*v*/*v*) ^b^	Temp. (°C)	Time (h)	Yield (%)
1	H_2_O	85	8	Trace
2	H_2_O/acetone (2:1)	60	12	66
3	H_2_O/THF (2:1)	65	12	86
4	H_2_O/DME (2:1)	85	12	63

^a^ Reaction conditions: the reactions were performed in a round-bottomed flask on a 0.25 mmol scale of **3l** and 0.3 mmol scale of **5a** in solvent (3 mL). THF = tetrahydrofuran. DME = 1,2-dimethoxyethane. ^b^ *v*/*v* = volume/volume.

**Table 3:** Cytotoxic activities of compounds **4a**–**4v** against cancerous cell lines ^a.^

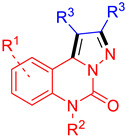								
Comp.	R^1^	R^2^	R^3^	A549 IC_50_ (μM)	MDA-MB-231 IC_50_ (μM)	tPSA (Å^2^)	ClogP	MW (kg/mol)
**4a**	H	Bn	COOMe	63.3	>100	88.5	2.28	391
**4b**	9-F	Bn	COOMe	99.4	>100	88.5	2.42	409
**4c**	9-Cl	Bn	COOMe	99.4	>100	88.5	2.99	426
**4d**	9-Br	Bn	COOMe	>100	85.8	88.5	3.14	470
**4e**	9-NO_2_	Bn	COOMe	>100	>100	140.3	2.02	436
**4f**	9-Me	Bn	COOMe	40.4	70.8	88.5	2.78	405
**4g**	9-OMe	Bn	COOMe	48.0	51.0	97.7	2.28	421
**4h**	H	Bn	COOEt	40.0	>100	88.5	3.34	419
**4i**	9-F	Bn	COOEt	17.0	75.3	88.5	3.48	437
**4j**	9-Cl	Bn	COOEt	36.7	65.8	88.5	4.05	453
**4k**	9-Br	Bn	COOEt	40.9	71.3	88.5	4.20	497
**4l**	9-NO_2_	Bn	COOEt	>100	>100	140.3	3.08	464
**4m**	9-Me	Bn	COOEt	14.2	>100	88.5	3.84	433
**4n**	9-OMe	Bn	COOEt	18.1	26.2	97.7	3.34	449
**4o**	7-F	Bn	COOEt	50.1	>100	88.5	3.48	437
**4p**	8,10-2F	Bn	COOEt	31.9	75.3	88.5	3.62	455
**4q**	H	Me	COOEt	31.0	93.0	88.5	1.57	343
**4r**	H	Boc	COOEt	37.3	74.2	114.8	2.50	429
**4s**	H	H	COOEt	56.2	>100	97.3	1.00	329
**4t**	H	H	COOMe	49.6	>100	97.3	−0.06	301
**4u**	9-F	Bn	Ph	46.4	>100	35.9	6.39	445
**4v**	H	H	Ph	44.7	22.5	44.7	3.91	337
**5-Fu**				21.2	24.6 ^b^	58.2	−0.58	130

^a^ 5-Fluorouracil (5-Fu) was used as positive controls. tPSA, ClogP and molecular weight (MW) values are calculated using the ACD/ChemSketch software. ^b^ The data reference source [36].

**Table 4:** The CDK2/7/9 activity of representative compounds **4h**, **4n**, and **4t** ^a^.

Comp.	CDK9		CDK2	CDK7
	IC_50_ (μM)	At 1.0 μM % of Activity	At 1.0 μM % of Activity	At 1.0 μM % of Activity
**4h**	n.d.	87.1% ± 0.2%	87.7% ± 5.2%	100.4% ± 1.8%
**4n**	9.8	75.0% ± 2.2%	90.0% ± 9.9%	102.7% ± 6.2%
**4t**	4.7	68.5% ± 0.2%	97.8% ± 1.6%	102.4% ± 4.5%

^a^ All data are reported as the average of duplicate experiments. n.d.: not determined.

**Table 5:** Pharmacokinetic aspects and drug-likeness parameters of compounds **4a**–**4v** ^a^.

	Pharmaco-Kinetic Aspects						Drug-Likeness (#Violations)					
Comp.	GI	BBB	P-gp	2D6	3A4	Lipinski	Ghose	Veber	Egan	Muegge	BS	PAINS
**4a**	High	No	No	No	No	0	0	0	0	0	0.55	0
**4b**	High	No	No	No	No	0	0	0	0	0	0.55	0
**4c**	High	No	No	No	No	0	0	0	0	0	0.55	0
**4d**	High	No	No	No	No	0	0	0	0	0	0.55	0
**4e**	High	No	No	No	Yes	1	0	0	1	0	0.55	0
**4f**	High	No	No	No	No	0	0	0	0	0	0.55	0
**4g**	High	No	No	No	Yes	0	0	0	0	0	0.55	0
**4h**	High	No	No	No	Yes	0	0	0	0	0	0.55	0
**4i**	High	No	No	No	Yes	0	0	0	0	0	0.55	0
**4j**	High	No	No	No	Yes	0	0	0	0	0	0.55	0
**4k**	High	No	No	No	Yes	0	1	0	0	0	0.55	0
**4l**	Low	No	No	No	Yes	1	0	0	1	0	0.55	0
**4m**	High	No	No	No	Yes	0	0	0	0	0	0.55	0
**4n**	High	No	No	No	Yes	0	0	0	0	0	0.55	0
**4o**	High	No	No	No	Yes	0	0	0	0	0	0.55	0
**4p**	High	No	No	No	Yes	0	0	0	0	0	0.55	0
**4q**	High	No	No	No	No	0	0	0	0	0	0.55	0
**4r**	High	No	No	No	Yes	0	0	0	0	0	0.55	0
**4s**	High	No	No	No	No	0	0	0	0	0	0.55	0
**4t**	High	No	No	No	No	0	0	0	0	0	0.55	0
**4u**	High	No	No	No	No	1	2	0	1	1	0.55	0
**4v**	High	Yes	Yes	No	No	1	0	0	0	0	0.55	0

^a^ GI: gastrointestinal absorption; BBB: blood–brain barrier; P-gp: poly-glycoprotein substrate; 2D6: CYP2D6 inhibitor; 3A4: CYP3A4 inhibitor; BS: bioavailability score; PAINS: pan-assay interference compounds alert.

**Table 6:** Toxicity risks of compounds **4a**–**4v**.

Comp.	Chronic Toxicity Risks ^a^ (OSIRIS)				Acute Oral Toxicity ^b^ (ProTox-II)	
	MUT	TUM	IRRIT	RE	LD_50_ (mg/kg)	Toxicity Class (1–6)
**4a**	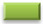	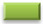	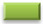	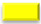	564	4
**4b**	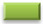	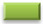	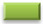	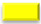	564	4
**4c**	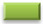	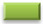	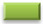	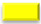	1140	4
**4d**	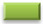	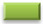	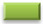	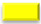	564	4
**4e**	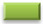	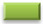	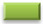	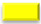	564	4
**4f**	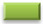	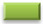	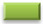	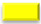	564	4
**4g**	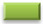	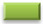	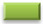	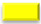	564	4
**4h**	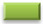	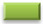	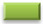	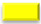	564	4
**4i**	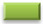	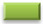	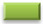	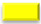	540	4
**4j**	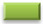	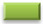	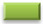	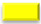	564	4
**4k**	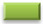	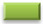	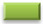	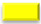	564	4
**4l**	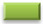	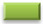	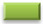	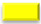	564	4
**4m**	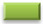	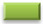	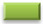	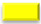	540	4
**4n**	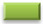	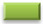	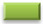	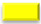	564	4
**4o**	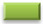	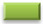	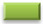	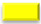	540	4
**4p**	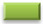	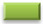	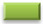	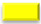	540	4
**4q**	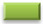	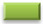	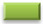	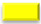	564	4
**4r**	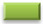	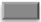	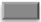	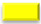	300	3
**4s**	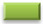	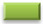	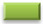	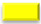	564	4
**4t**	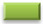	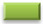	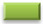	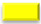	564	4
**4u**	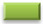	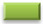	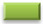	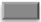	1000	4
**4v**	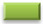	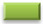	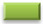	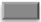	1000	4

^a^ The results are colour-coded as grey, green, and yellow. Green colour suggests low toxic potential, yellow means mild toxicity, and grey colour indicates high probability of toxicity. ^b^ Toxicity class 1–6 (1 = high risk, 6 = low risk). Class 1: fatal if swallowed (LD_50_ ≤ 5 mg/kg), class 2: fatal if swallowed (5 < LD_50_ ≤ 50 mg/kg), class 3: toxic if swallowed (50 < LD_50_ ≤ 300 mg/kg), class 4: harmful if swallowed (300 < LD_50_ ≤ 2000 mg/kg), class 5: may be harmful if swallowed (2000 < LD_50_ ≤ 5000 mg/kg), and class 6: nontoxic (LD_50_ > 5000 mg/kg).

## Data Availability

The data presented in this study are available upon request from the corresponding author.

## Supplementary Materials

The following supporting information can be downloaded at: https://www.mdpi.com/article/10.3390/molecules28186606/s1. Table S1. Green synthesis of **4k**/**4o** (page 2). Table S2. Cell growth inhibition of the pyrazolo-[1,5-c]quinazolinone derivatives on A549 cells, MDA-MB-231 cells, U87 cells, and HepG2 cells (30 μM) (page 3). Figure S1. Compound IC_50_ data for CDK9 inhibition (page 5). Figure S2. The ProTox-II toxicity profiles for compounds **4a**–**4v** (page 6). Figure S3. Copies of NMR spectra and HRMS spectra for compounds **4a**–**4v** (page 14). Figure S4. Copies of HPLC analysis for products **4a**–**4v** (page 58). Biology methods (page 69). In silico study methods (page 70).

## Sample Availability

Samples of the compound are available from the authors.
