# Supplementary material for: Design, Synthesis, Antitumour Evaluation, and In Silico Studies of Pyrazolo-[1,5-c]quinazolinone Derivatives Targeting Potential Cyclin-Dependent Kinases
Source: Molecules. 2023 Sep 13;28(18):6606. doi: 10.3390/molecules28186606 (PMC10536637; doi:10.3390/molecules28186606)
Supplement: Supplementary file 1 [file molecules-28-06606-s001.zip › molecules-2527425-supplementary.pdf]

Design, Synthesis, Antitumour Evaluation, and In Silico Studies of Pyrazolo-[1,5-c]quinazolinone Derivatives Targeting Potential Cyclin-Dependent Kinases

Danyang Zheng [1,†], Chenqi Yang [1,†], Xiaogang Li [1,†], Dong Liu [1], Yan Wang [1], Xuesong Wang [1], Xueying Zhang [1], Yinfeng Tan [1], Yuchen Zhang [1], Dulin Kong [1], Youbin Li [1,*] and Junyu Xu [1,*]

[1] Key Laboratory of Tropical Translational Medicine of Ministry of Education, Hainan Provincial Key Laboratory for Research and Development of Tropical Herbs, Haikou Key Laboratory of Li Nationality Medicine, School of Pharmacy, Hainan Medical University, Haikou 571199, China.

*Corresponding authors, E-mails: xujy201309@hainmc.edu.cn (J. Xu), liyoubinli@sohu.com (Y. Li).
† These authors contributed equally to this work.

Table of contents

1. **Table S1**. Green synthesis of **4k**/**4o** 2

2. **Table S2**. Cell growth inhibition of the pyrazolo-[1,5-*c*]quinazolinone derivatives on A549 cells, MDA-MB-231 cells, U87 cells and HepG2 cells (30 μM). 3

3. **Figure S1**. Compound IC_{50} data for CDK9 inhibition. 5

4. **Figure S2.** The ProTox-II toxicity profiles for compounds **4a** – **4v**. 6

5. **Figure S3.** Copies of NMR Spectra and HRMS Spectra for compounds **4a** – **4v** 14

6. **Figure S4.** Copies of HPLC analysis for products **4a** – **4v** 58

7. **Biology methods** 69

8. ***In silico* study methods** 70

1. **Table S1**. Green synthesis of **4k**/**4o**

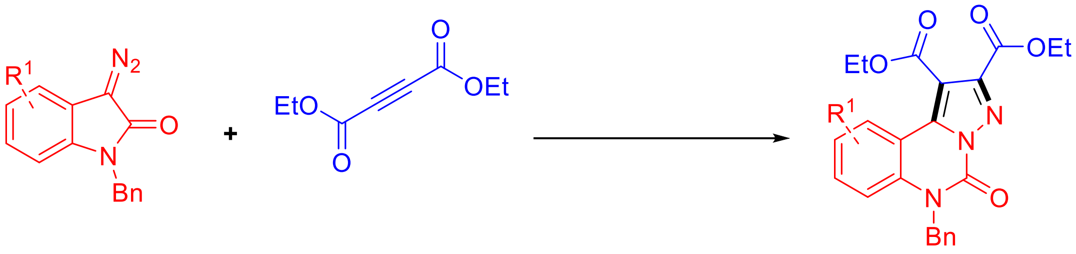

3d, R^1 = 5-Br
3h, R^1 = 7-F

5b

4k, R^1 = 8-Br
4o, R^1 = 6-F

**Entry**	**Reactant 3**	**Product 4**	**Solvent**	**Temp. (°C)**	**Time (h)**	**Yield (%)**
1	**3d**	**4k**	H_2O/Acetone (2:1)	60	12	73
2	**3d**	**4k**	H_2O/THF (2:1)	65	12	90
3	**3h**	**4o**	H_2O/Acetone (2:1)	60	12	68
4	**3h**	**4o**	H_2O/THF (2:1)	65	12	88

[a] Reaction conditions: the reactions were performed in a round-bottom flask on a 0.25 mmol scale of **3d/ 3h** and 0.3 mmol scale of **5b** in solvent (3 mL). THF = Tetrahydrofuran. DME = 1,2-dimethoxyethane.

2. Table S2. Cell growth inhibition of the pyrazolo-[1,5-*c*]quinazolinone derivatives on A549 cells, MDA-MB-231 cells, U87 cells and HepG2 cells (30 μM).

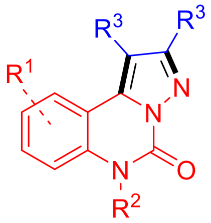

Comp.	R^1	R^2	R^3	Inhibition Rate [a]			
				**A549**	**MDA-MB-231**	**U87**	**HepG2**
**4a**	H	Bn	MeO	34%±4%	11%±3%	30%±4%	14%±5%
**4b**	9-F	Bn	MeO	24%±5%	12%±3%	9%±0%	37%±5%
**4c**	9-Cl	Bn	MeO	30%±4%	24%±4%	n.d.	8%±0%
**4d**	9-Br	Bn	MeO	32%±4%	11%±2%	34%±4%	7%±3%
**4e**	9-NO_2	Bn	MeO	22%±3%	27%±3%	21%±1%	-1%±2%
**4f**	9-Me	Bn	MeO	44%±3%	37%±4%	37%±4%	4%±2%
**4g**	9-OMe	Bn	MeO	45%±2%	41%±6%	12%±5%	18%±1%
**4h**	H	Bn	EtO	53%±2%	14%±2%	1%±2%	2%±0%
**4i**	9-F	Bn	EtO	63%±2%	29%±5%	-2%±1%	0%±3%
**4j**	9-Cl	Bn	EtO	47%±1%	14%±3%	52%±11%	-2%±4%
**4k**	9-Br	Bn	EtO	41%±3%	40%±5%	48%±4%	5%±4%
**4l**	9-NO_2	Bn	EtO	18%±4%	11%±5%	16%±5%	11%±2%
**4m**	9-Me	Bn	EtO	77%±2%	23%±3%	38%±3%	-1%±5%
**4n**	9-OMe	Bn	EtO	66%±2%	54%±7%	19%±8%	13%±3%
**4o**	7-F	Bn	EtO	19%±5%	38%±4%	22%±1%	5%±2%
**4p**	8,10-2F	Bn	EtO	40%±6%	29%±5%	-7%±1%	-1%±3%
**4q**	H	Me	EtO	46%±2%	23%±4%	2%±2%	24%±6%
**4r**	H	BOC	EtO	48%±3%	13%±2%	13%±3%	24%±6%
**4s**	H	H	EtO	30%±5%	13%±3%	2%±2%	2%±3%
**4t**	H	H	MeO	35%±3%	11%±4%	10%±4%	5%±3%

**4u**	9-F	Bn	Ph	27%±8%	15%±4%	20%±4%	17%±5%
**4v**	H	Bn	Ph	31%±1%	60%±7%	25%±6%	88%±3%
**5-Fu**				69%±1%	58%±4%	n.d.	58%±1%
**Abe**				83%±1%	88%±1%	86%±1%	86%±1%

[a] Cells were treated with compounds at the concentration of 30 μM for 72 h, the data represent mean ± SD (n = 3). 5-Fluorouracil (5-Fu) and Abemaciclib (Abe) were used as positive controls. n.d.: not determined.

3. Figure S1. Compound IC_{50} data for CDK9 inhibition.

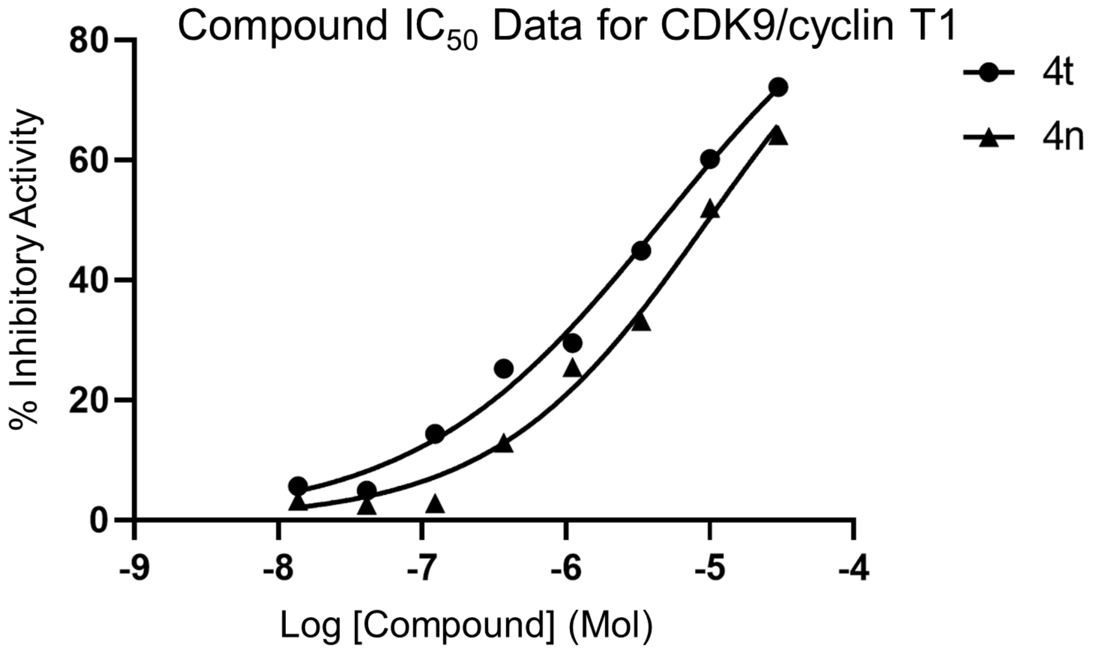

4. **Figure S2.** The ProTox-II toxicity profiles for compounds **4a – 4v**.

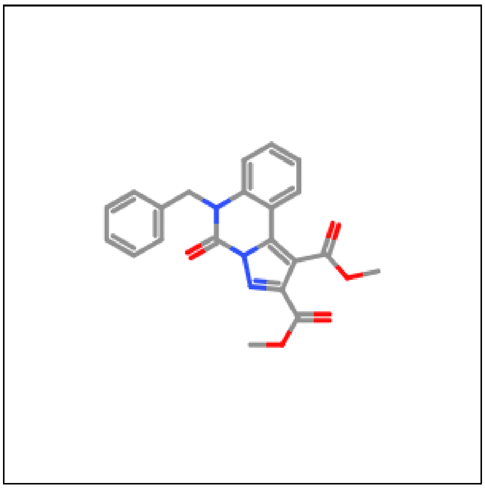

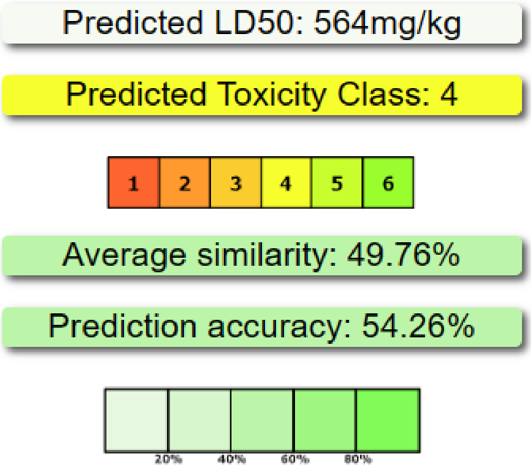

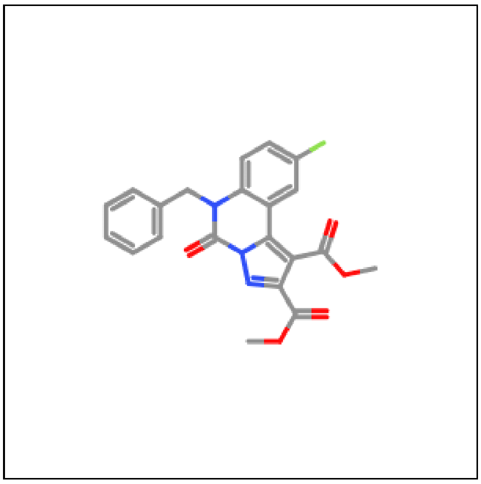

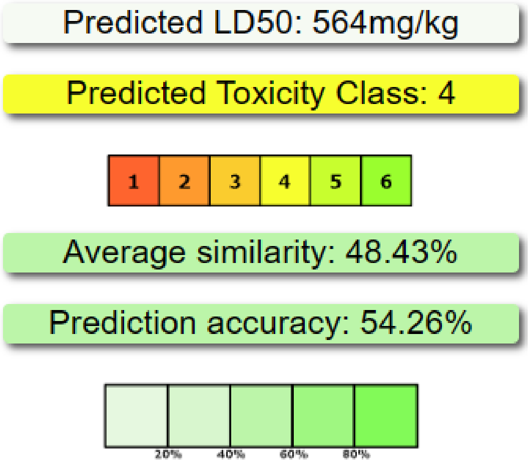

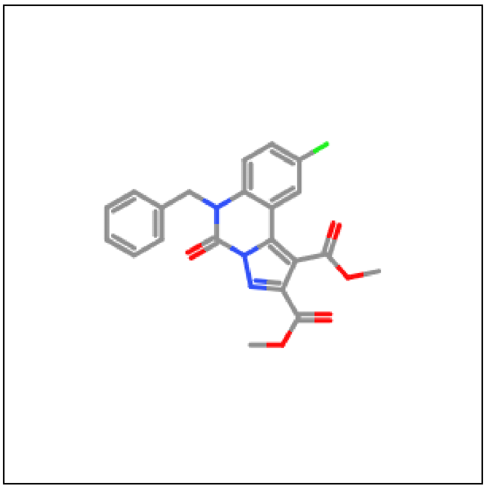

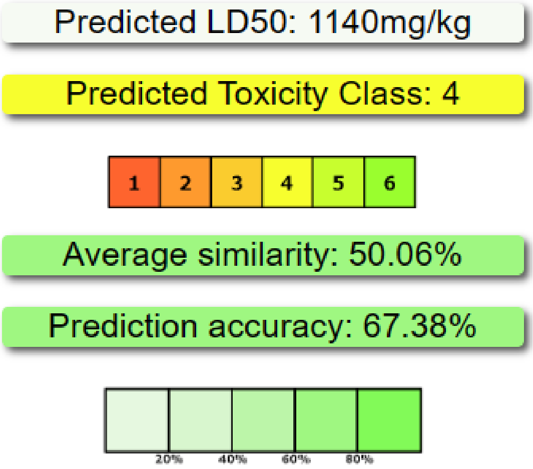

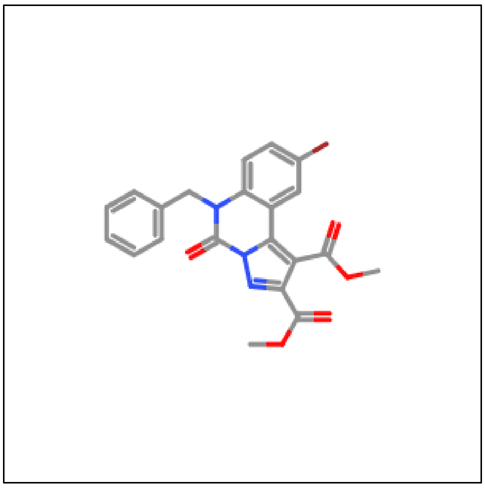

Predicted LD50: 564mg/kg

Predicted Toxicity Class: 4

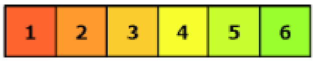

Average similarity: 47.95%

Prediction accuracy: 54.26%

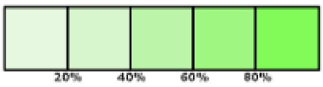

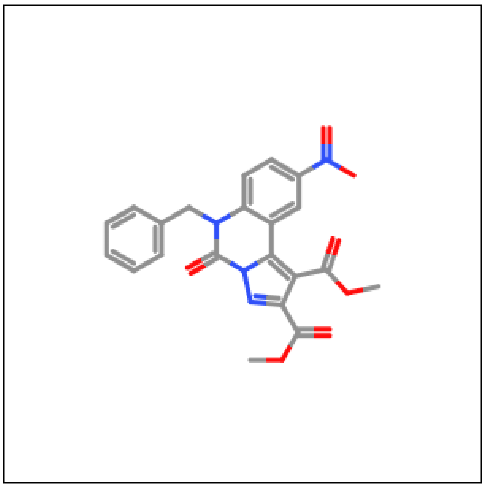

Predicted LD50: 564mg/kg

Predicted Toxicity Class: 4

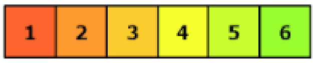

Average similarity: 47.53%

Prediction accuracy: 54.26%

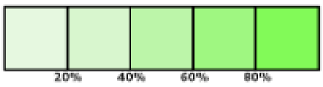

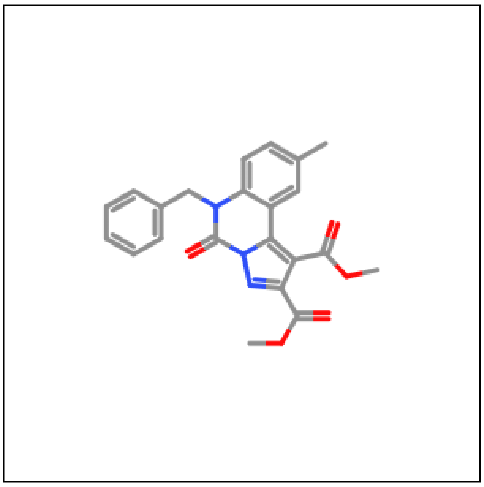

Predicted LD50: 564mg/kg

Predicted Toxicity Class: 4

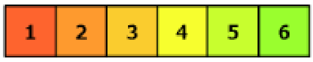

Average similarity: 50.26%

Prediction accuracy: 67.38%

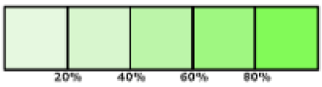

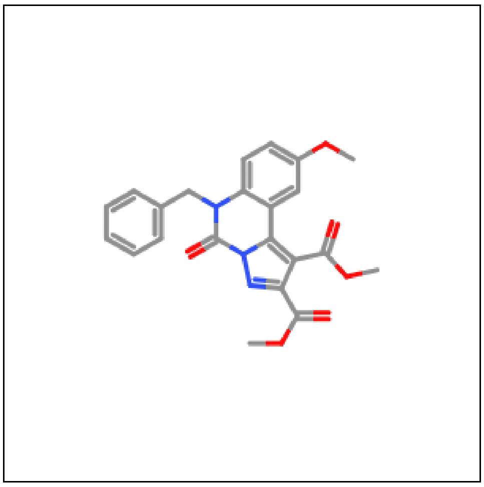

Predicted LD50: 564mg/kg

Predicted Toxicity Class: 4

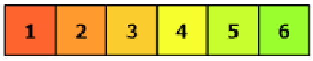

Average similarity: 48.85%

Prediction accuracy: 54.26%

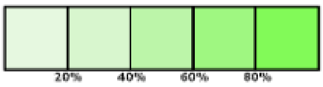

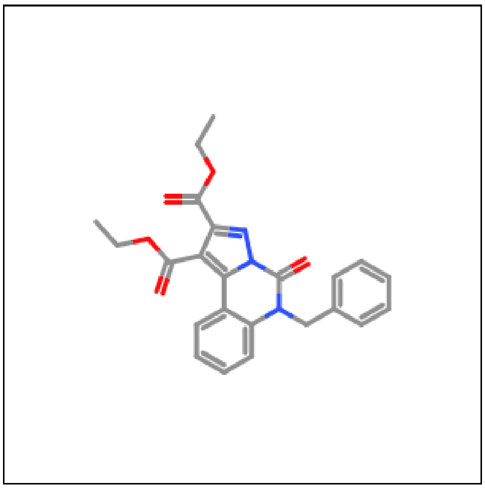

Predicted LD50: 564mg/kg

Predicted Toxicity Class: 4

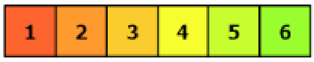

Average similarity: 50.05%

Prediction accuracy: 67.38%

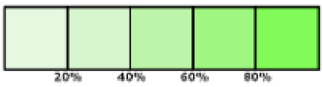

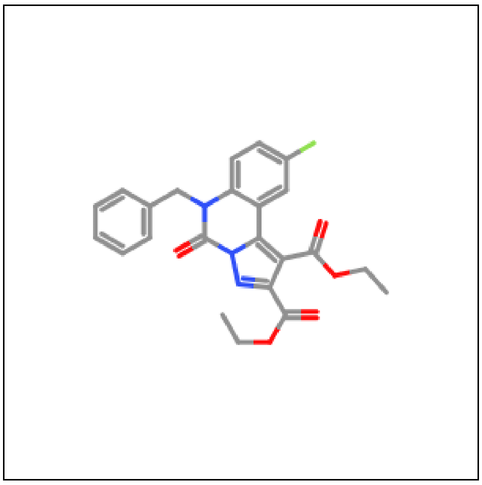

Predicted LD50: 540mg/kg

Predicted Toxicity Class: 4

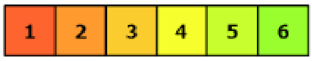

Average similarity: 49.29%

Prediction accuracy: 54.26%

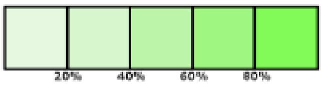

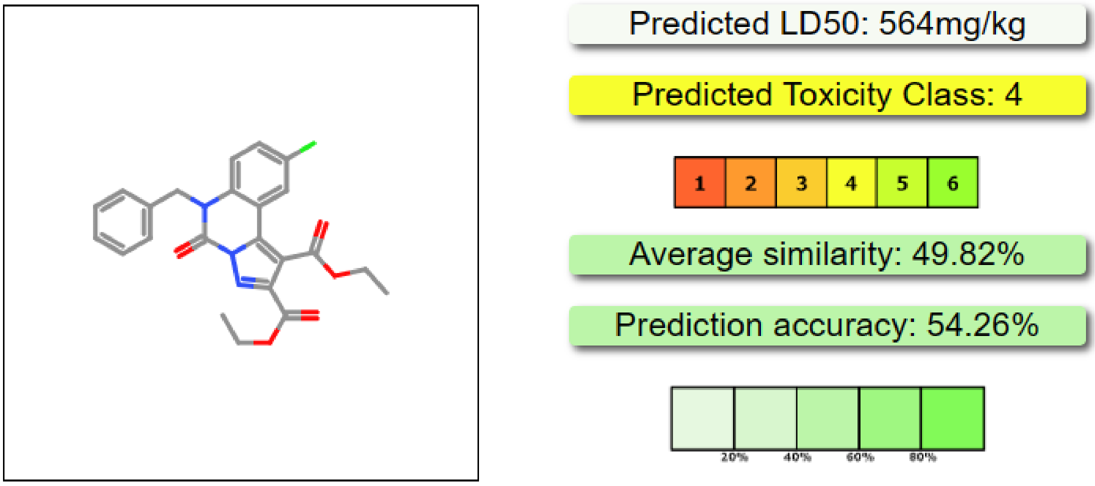
Predicted LD50: 564mg/kg
Predicted Toxicity Class: 4
1 2 3 4 5 6
Average similarity: 49.82%
Prediction accuracy: 54.26%
20% 40% 60% 80%

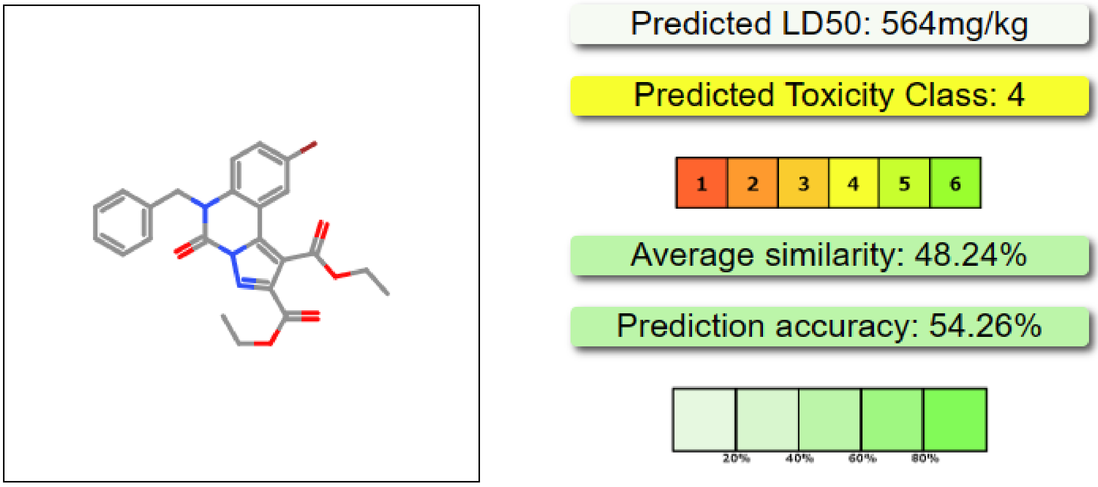
Predicted LD50: 564mg/kg
Predicted Toxicity Class: 4
1 2 3 4 5 6
Average similarity: 48.24%
Prediction accuracy: 54.26%
20% 40% 60% 80%

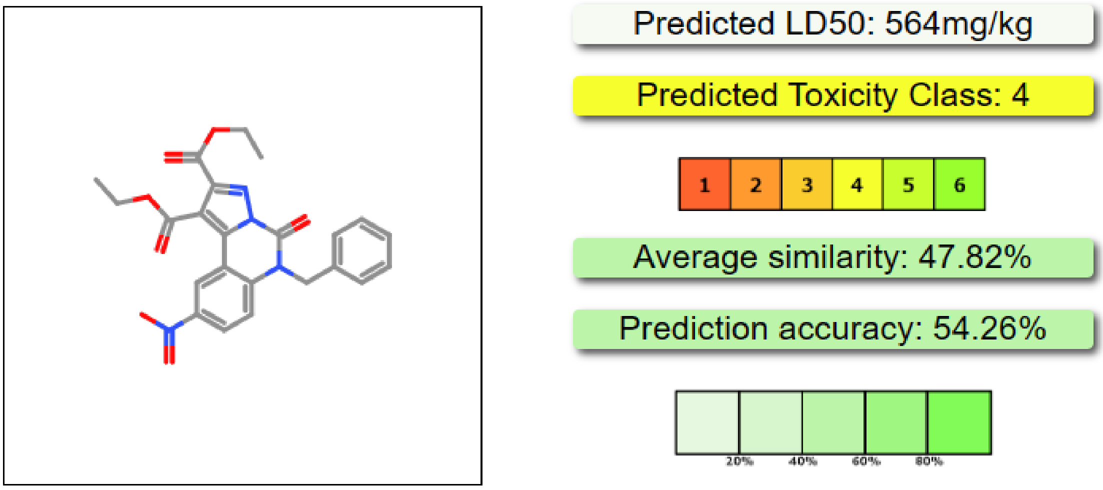
Predicted LD50: 564mg/kg
Predicted Toxicity Class: 4
1 2 3 4 5 6
Average similarity: 47.82%
Prediction accuracy: 54.26%
20% 40% 60% 80%

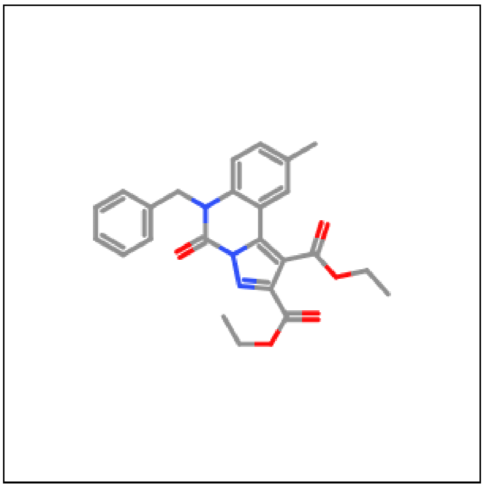

Predicted LD50: 540mg/kg

Predicted Toxicity Class: 4

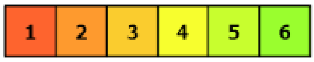

Average similarity: 50.63%

Prediction accuracy: 67.38%

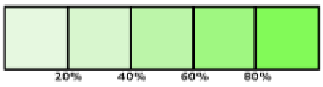

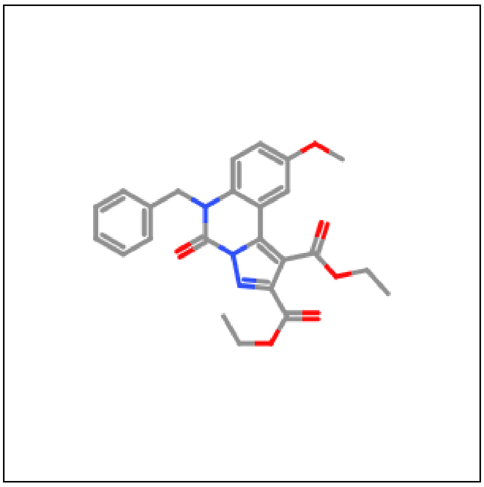

Predicted LD50: 564mg/kg

Predicted Toxicity Class: 4

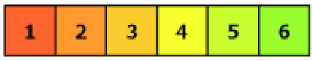

Average similarity: 49.14%

Prediction accuracy: 54.26%

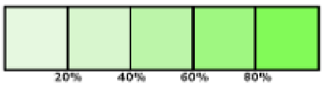

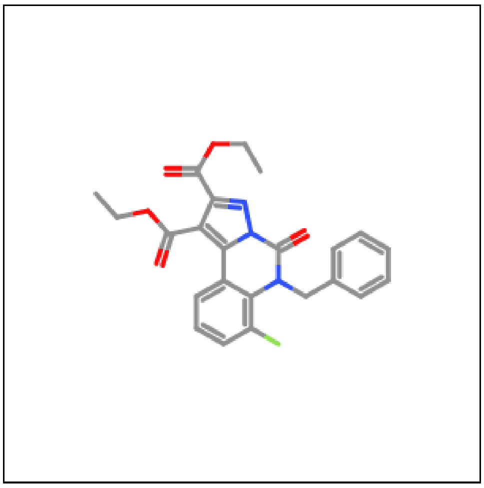

Predicted LD50: 540mg/kg

Predicted Toxicity Class: 4

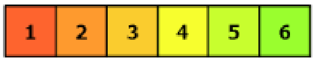

Average similarity: 48.39%

Prediction accuracy: 54.26%

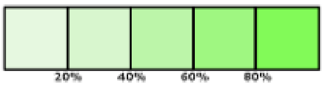

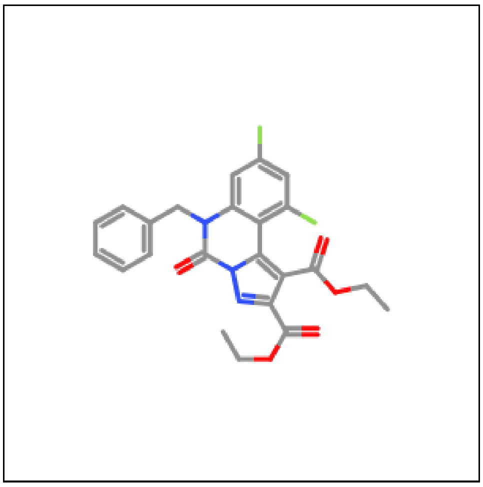

Predicted LD50: 540mg/kg

Predicted Toxicity Class: 4

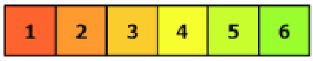

Average similarity: 48.19%

Prediction accuracy: 54.26%

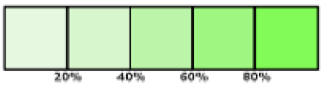

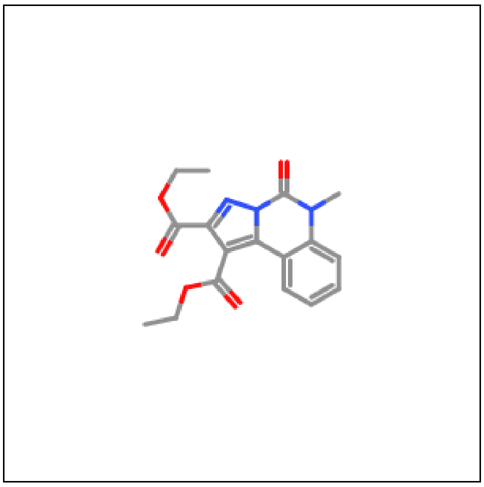

Predicted LD50: 564mg/kg

Predicted Toxicity Class: 4

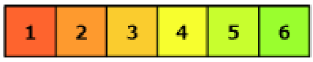

Average similarity: 46.46%

Prediction accuracy: 54.26%

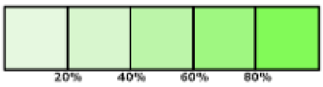

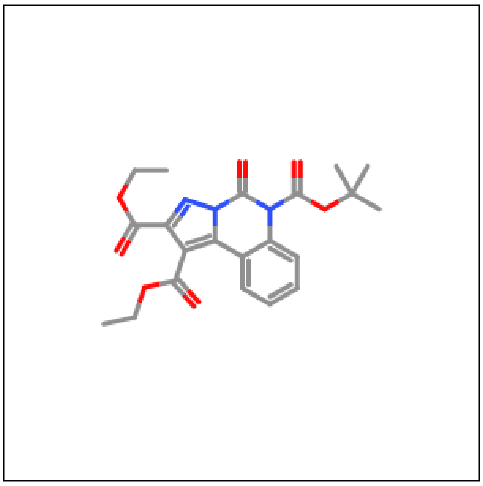

Predicted LD50: 300mg/kg

Predicted Toxicity Class: 3

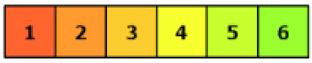

Average similarity: 43.95%

Prediction accuracy: 54.26%

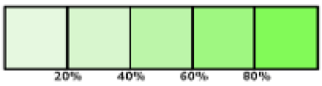

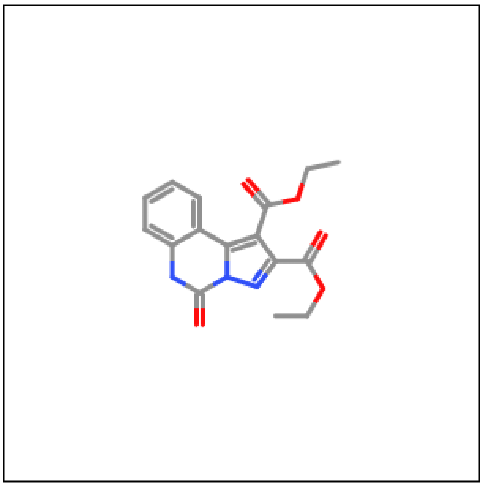

Predicted LD50: 564mg/kg

Predicted Toxicity Class: 4

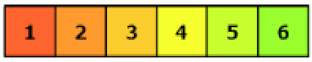

Average similarity: 46.36%

Prediction accuracy: 54.26%

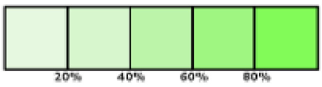

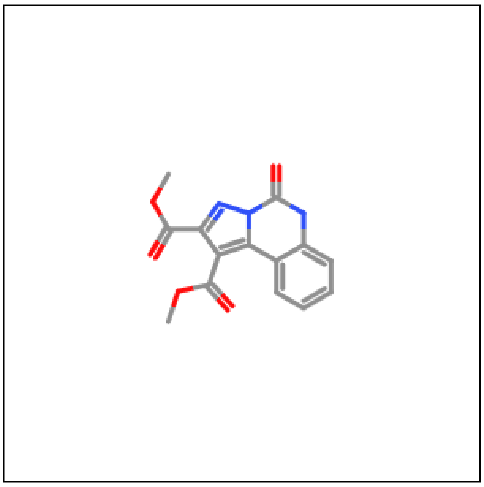

Predicted LD50: 564mg/kg

Predicted Toxicity Class: 4

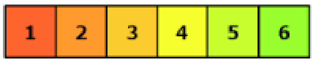

Average similarity: 45.49%

Prediction accuracy: 54.26%

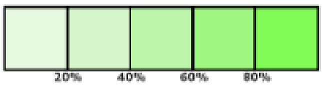

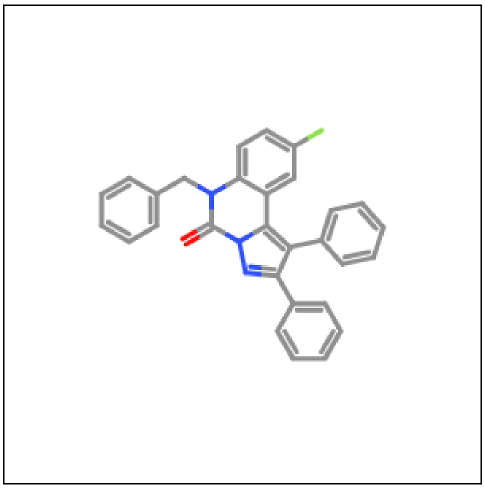

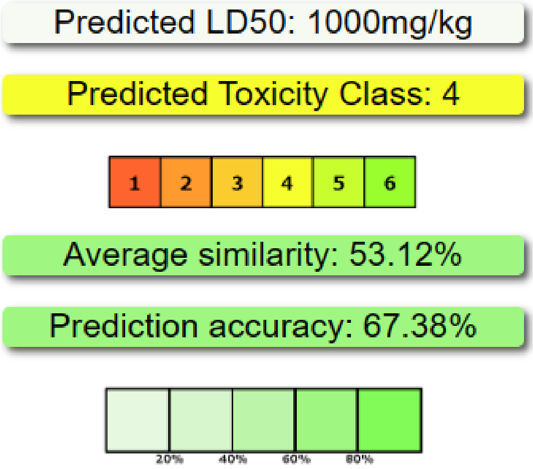
Predicted LD50: 1000mg/kg
Predicted Toxicity Class: 4
1 2 3 4 5 6
Average similarity: 53.12%
Prediction accuracy: 67.38%
20% 40% 60% 80%

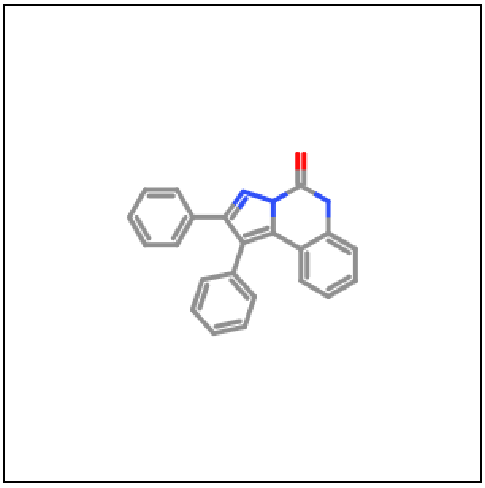

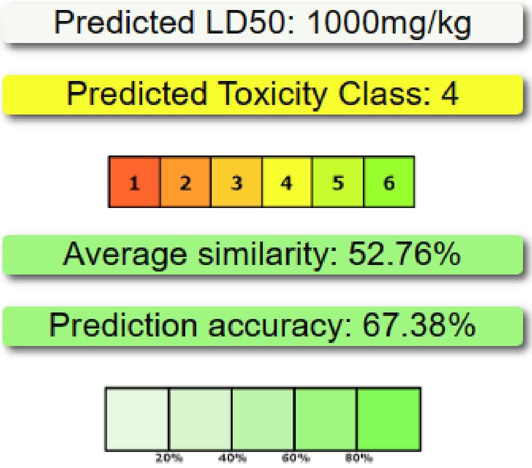
Predicted LD50: 1000mg/kg
Predicted Toxicity Class: 4
1 2 3 4 5 6
Average similarity: 52.76%
Prediction accuracy: 67.38%
20% 40% 60% 80%

5. **Figure S3.**Copies of NMR Spectra and HRMS Spectra for compounds **4a** – **4v**

Compound **4a**

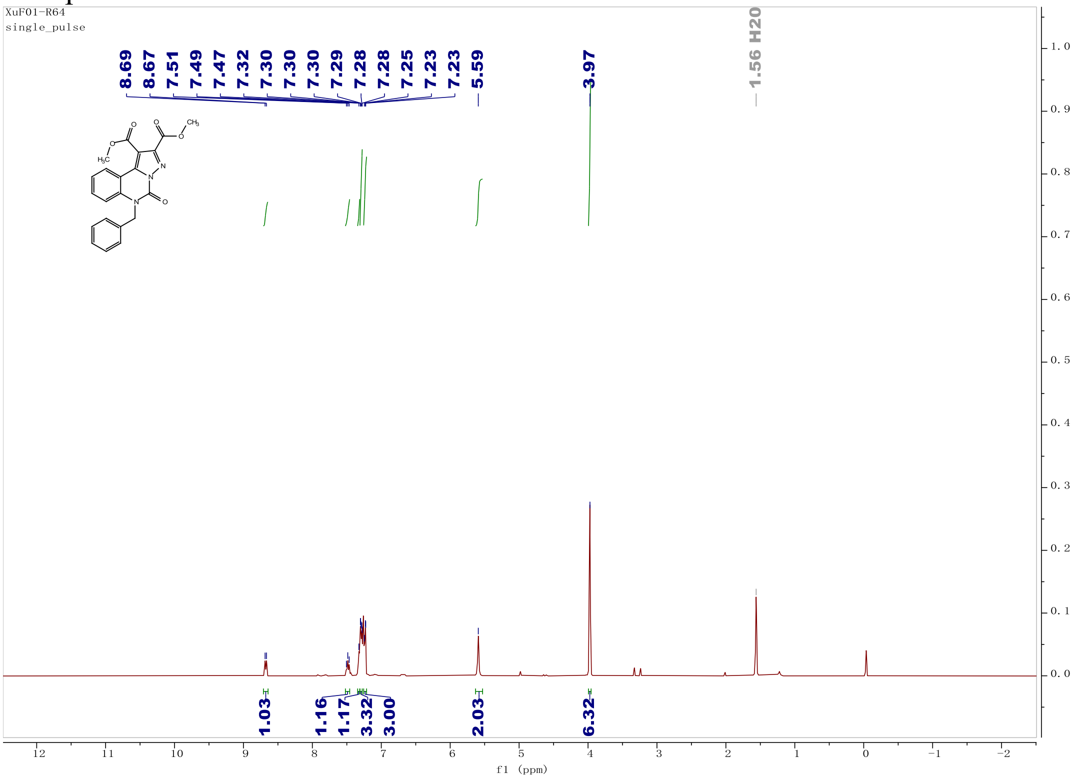

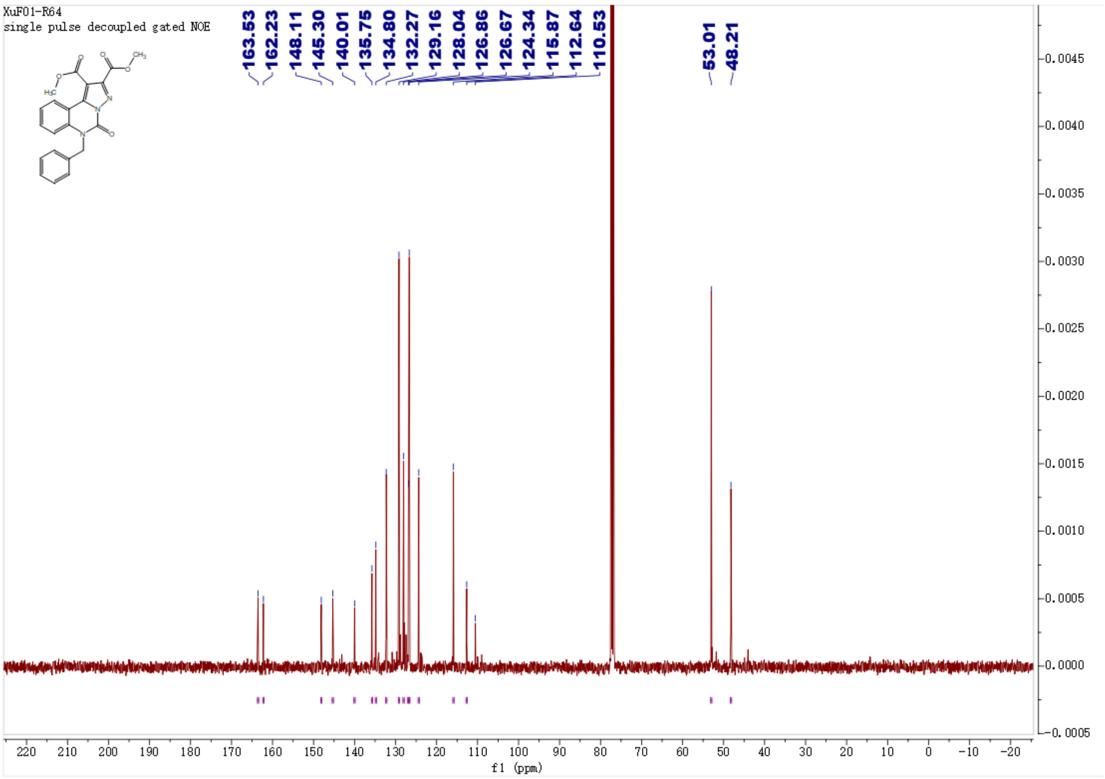

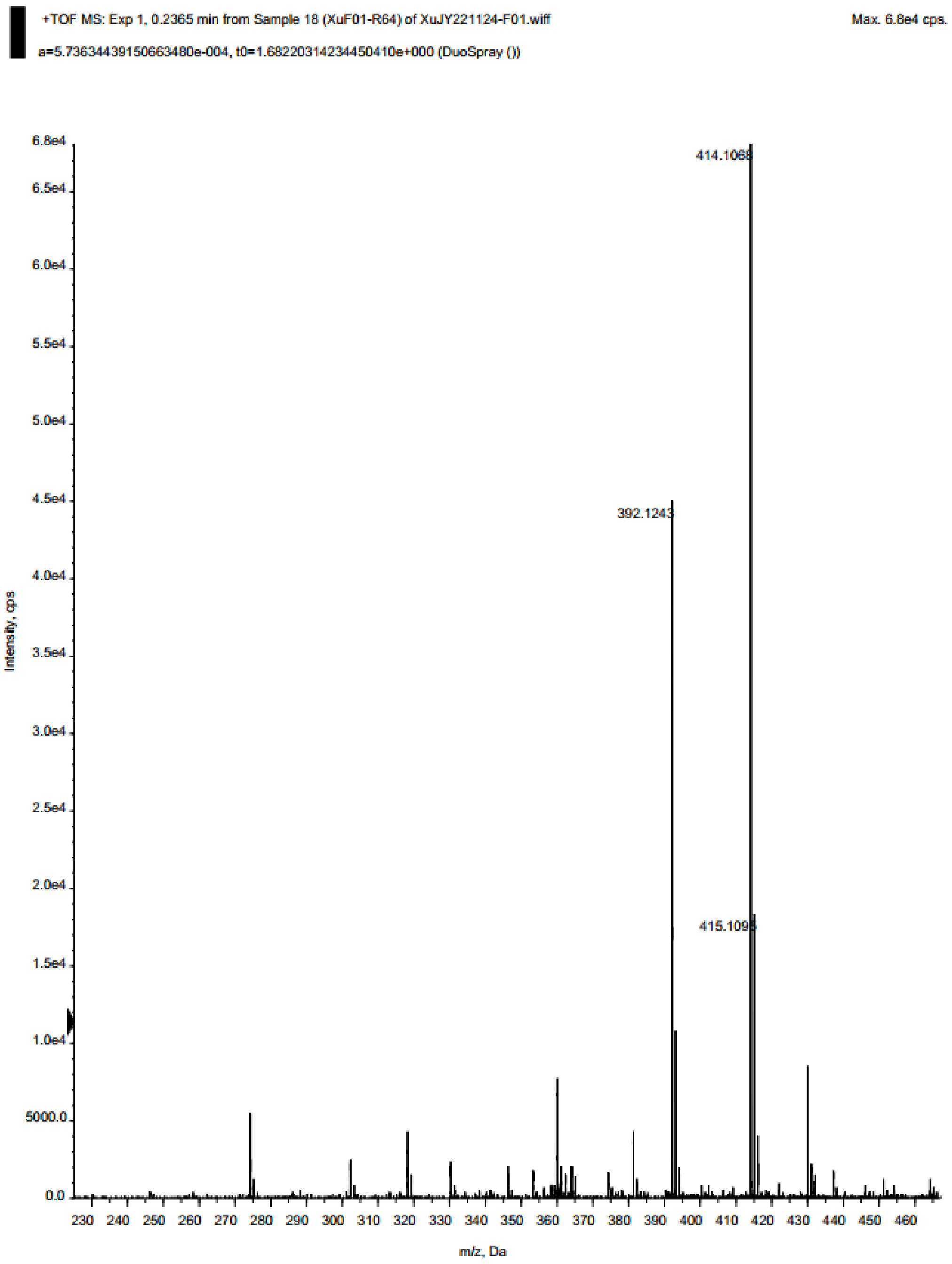
+TOF MS: Exp 1, 0.2365 min from Sample 18 (XuF01-R64) of XuJY221124-F01.wiff
Max. 6.8e4 cps.
a=5.73634439150663480e-004, t0=1.68220314234450410e+000 (DuoSpray ())
414.1068
392.1243
415.1098
Intensity, cps
m/z, Da

Compound **4b**

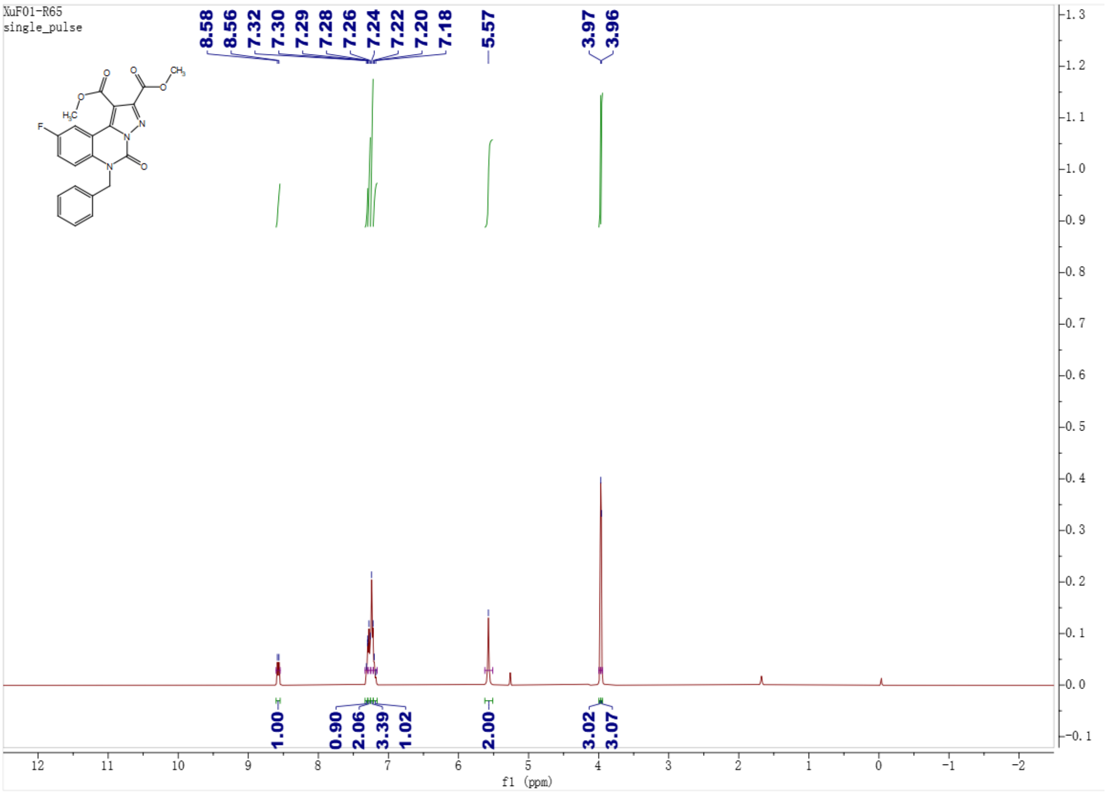
XuF01-R65
single_pulse
8.58 8.56 7.32 7.30 7.29 7.28 7.26 7.24 7.22 7.20 7.18
5.57
3.97 3.96
1.00 0.90 2.06 3.39 1.02 2.00 3.02 3.07
f1 (ppm)

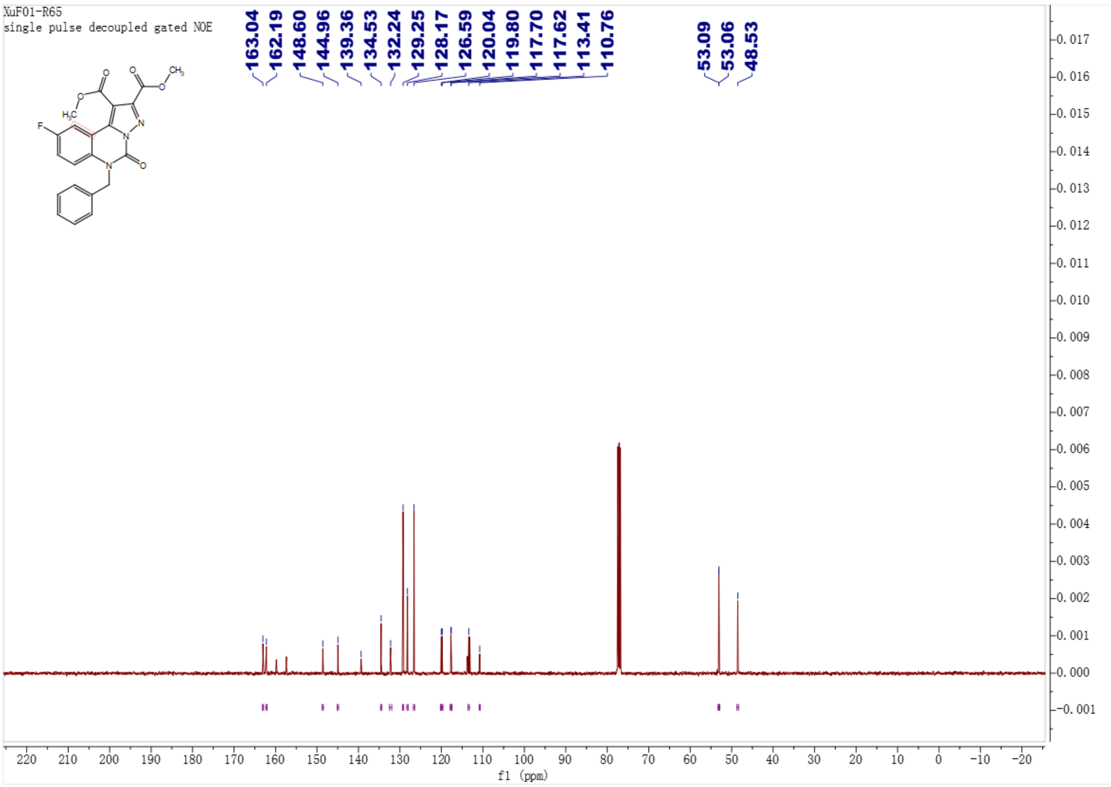
XuF01-R65
single pulse decoupled gated NOE
163.04 162.19 148.60 144.96 139.36 134.53 132.24 129.25 128.17 126.59 120.04 119.80 117.70 117.62 113.41 110.76
53.09 53.06 48.53
f1 (ppm)

+TOF MS: Exp 1, 0.2344 min from Sample 19 (XuF01-R65) of XuJY221124-F01.wiff

Max. 9.5e4 cps.

a=5.73634439150663480e-004, t0=1.68220314234450410e+000 (DuoSpray ())

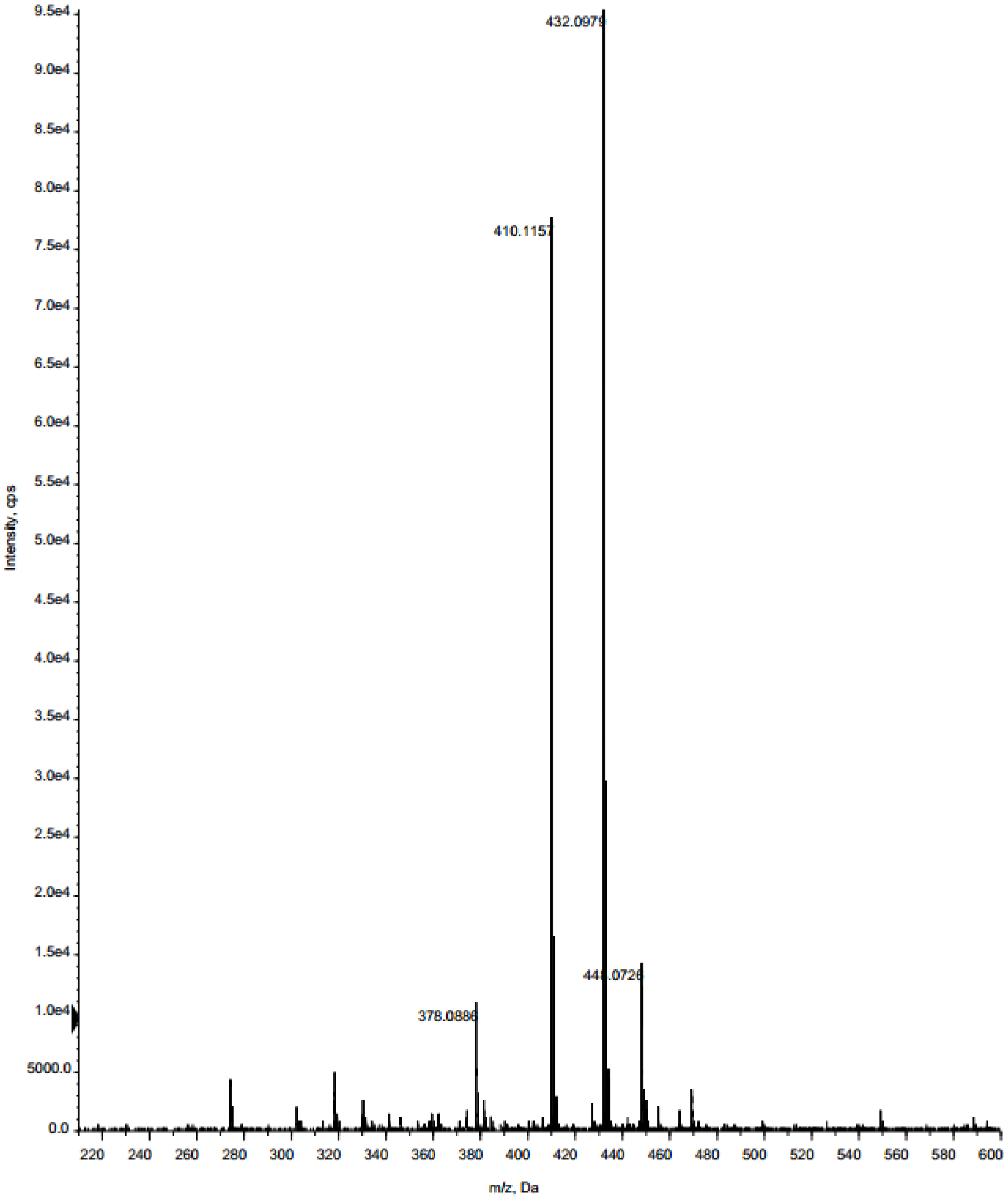

Compound **4c**

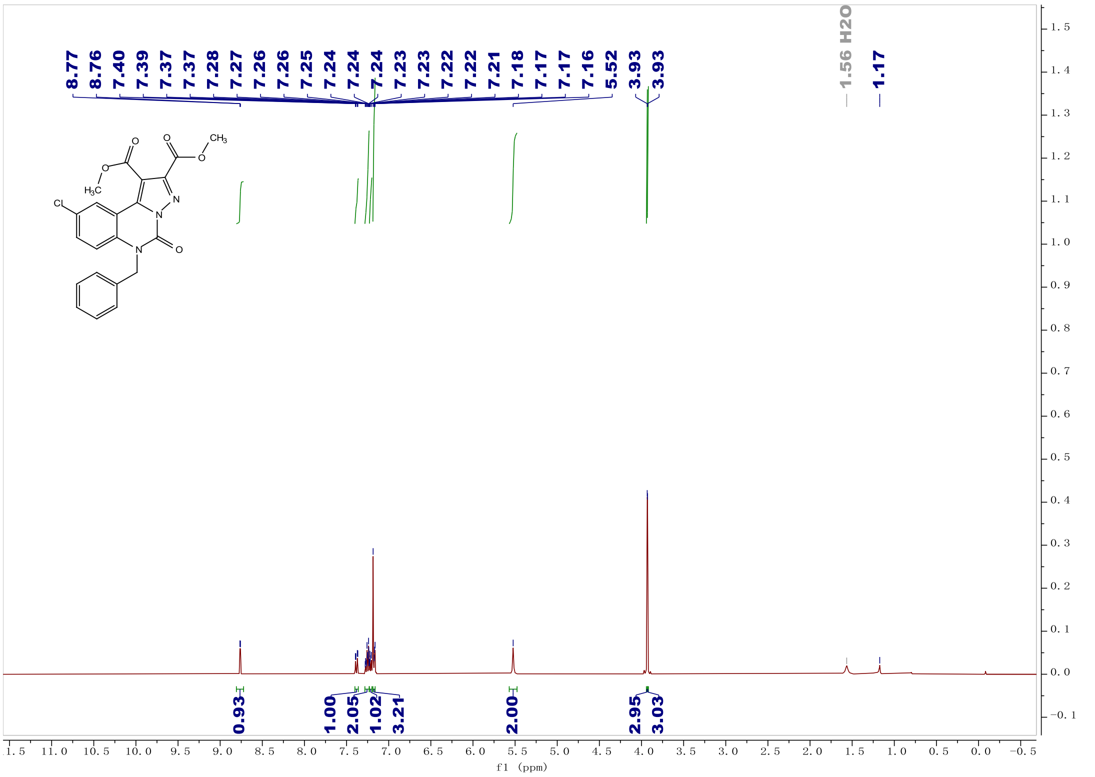

8.77 8.76 7.40 7.39 7.37 7.37 7.28 7.27 7.26 7.26 7.25 7.24 7.24 7.24 7.23 7.23 7.22 7.22 7.21 7.18 7.17 7.17 7.16 5.52 3.93 3.93
1.56 H2O
1.17
0.93 1.00 2.05 1.02 3.21 2.00 2.95 3.03
f1 (ppm)

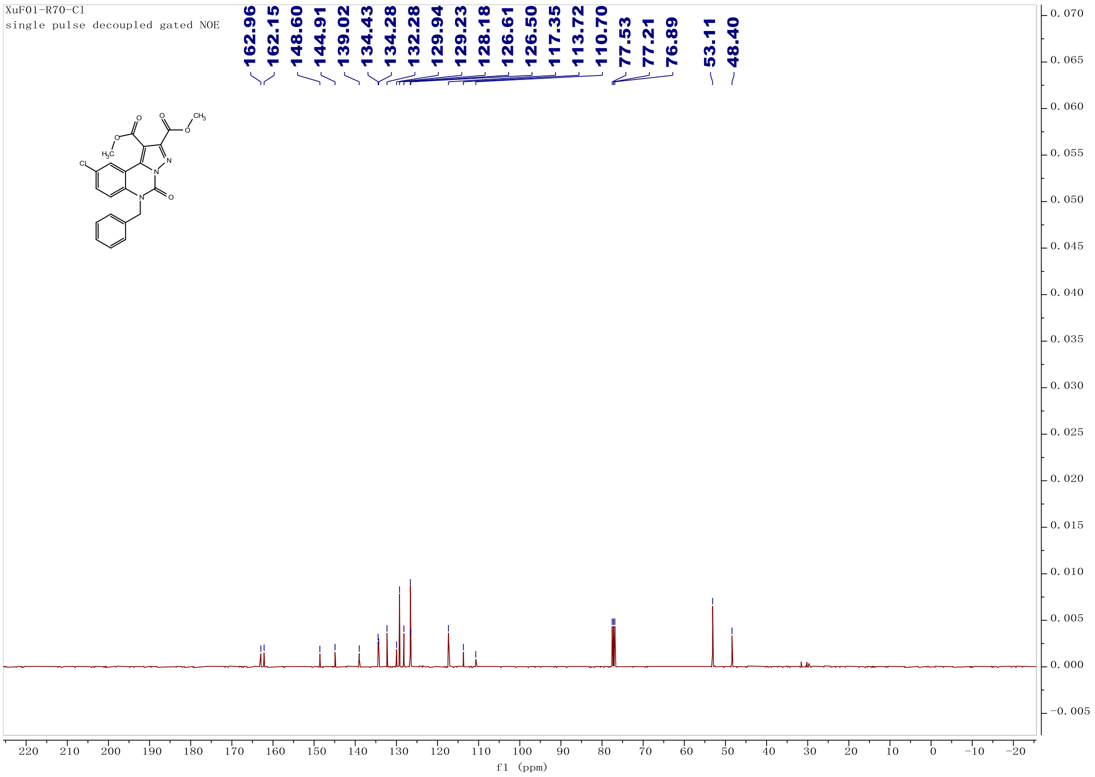

XuF01-R70-Cl
single pulse decoupled gated NOE
162.96 162.15 148.60 144.91 139.02 134.43 134.28 132.28 129.94 129.23 128.18 126.61 126.50 117.35 113.72 110.70 77.53 77.21 76.89 53.11 48.40
f1 (ppm)

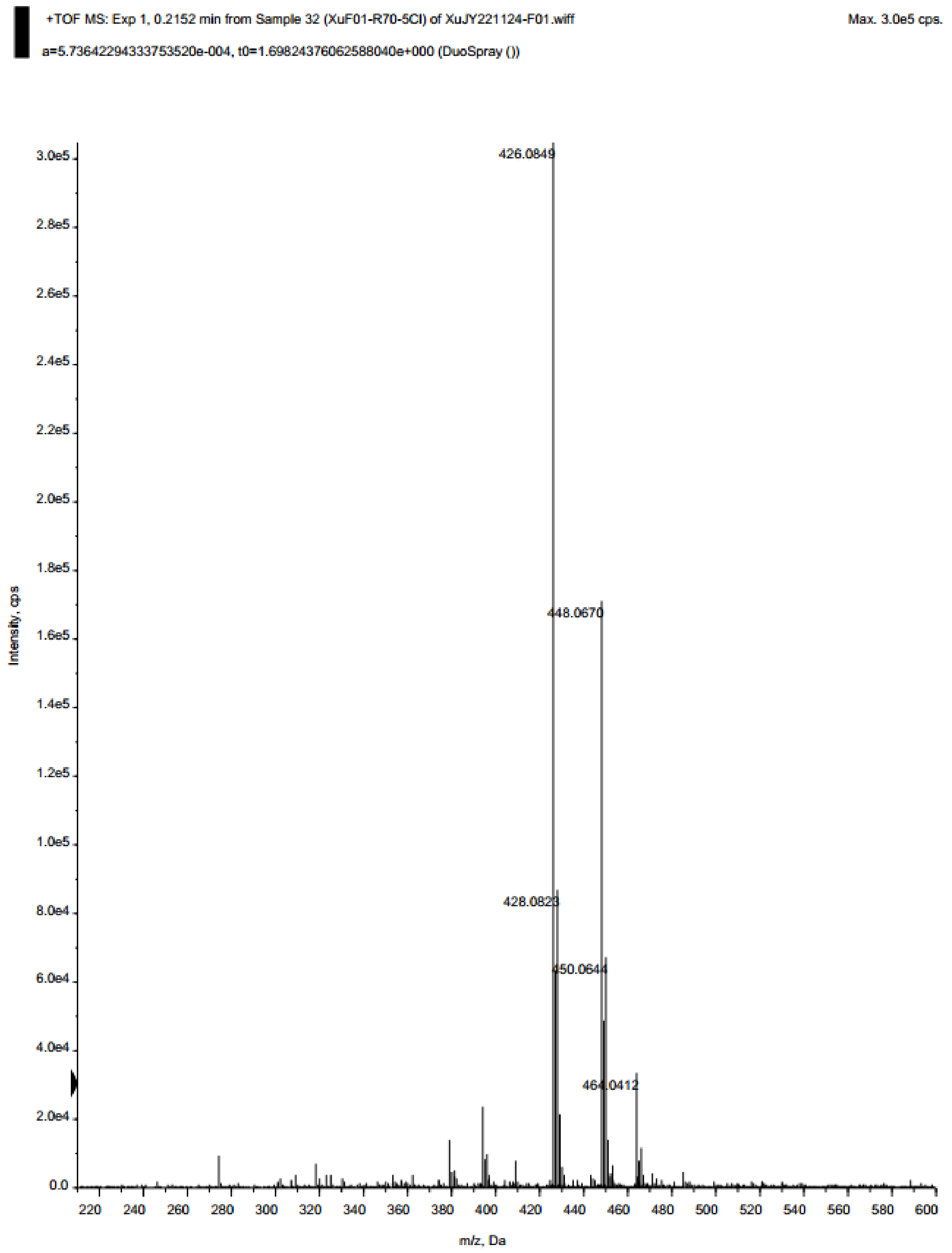

+TOF MS: Exp 1, 0.2152 min from Sample 32 (XuF01-R70-5Cl) of XuJY221124-F01.wiff
Max. 3.0e5 cps.
a=5.73642294333753520e-004, t0=1.69824376062588040e+000 (DuoSpray ())
426.0849
448.0670
428.0823
450.0644
464.0412
Intensity, cps
m/z, Da

Compound **4d**

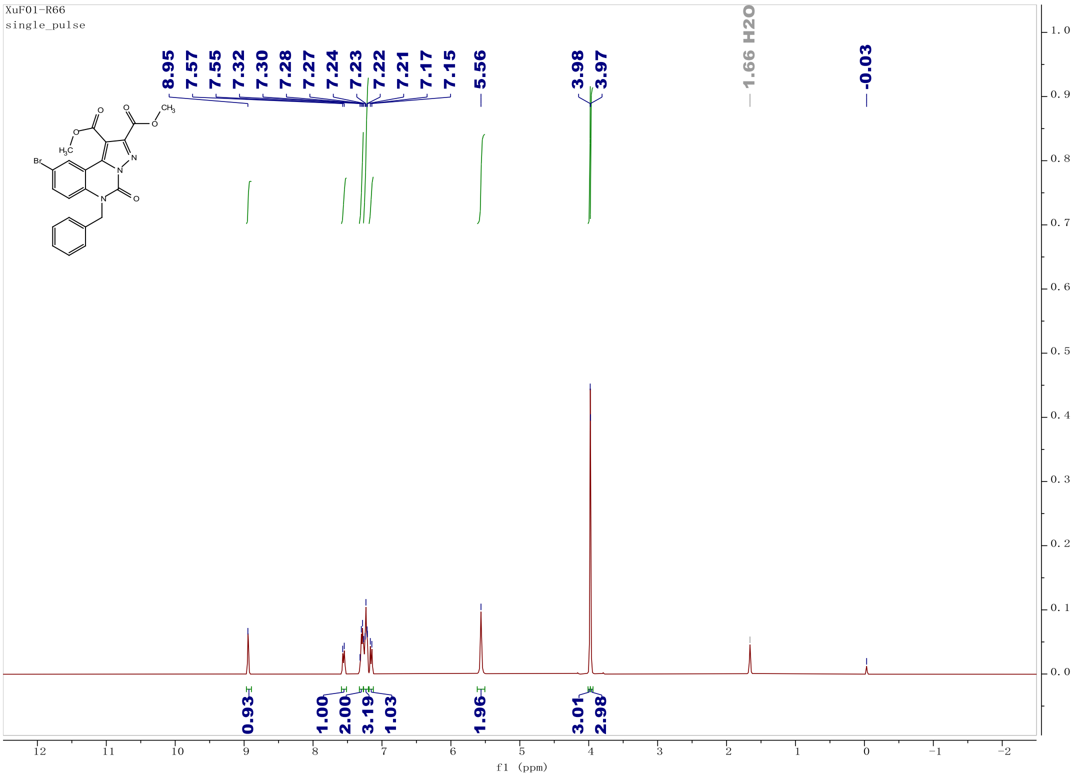

XuF01-R66
single_pulse
8.95
7.57
7.55
7.32
7.30
7.28
7.27
7.24
7.23
7.22
7.21
7.17
7.15
5.56
3.98
3.97
1.66 H2O
-0.03
0.93
1.00
2.00
3.19
1.03
1.96
3.01
2.98
f1 (ppm)

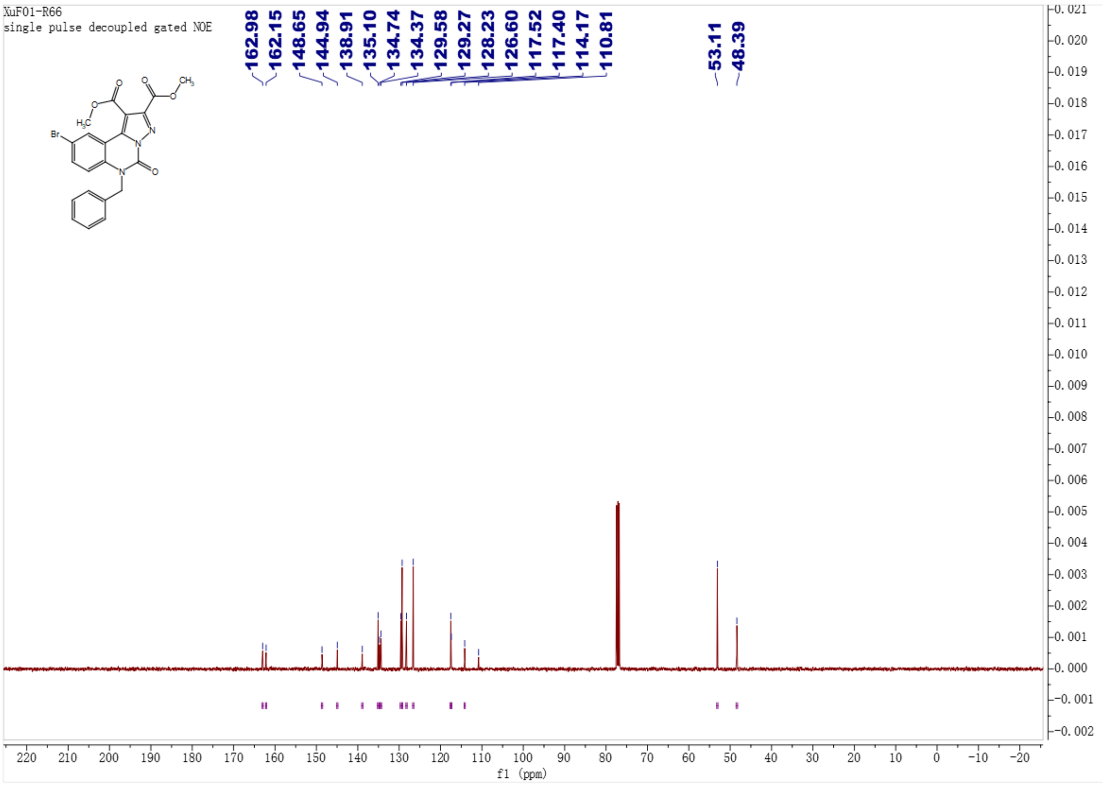

XuF01-R66
single pulse decoupled gated NOE
162.98
162.15
148.65
144.94
138.91
135.10
134.74
134.37
129.58
129.27
128.23
126.60
117.52
117.40
114.17
110.81
53.11
48.39
f1 (ppm)

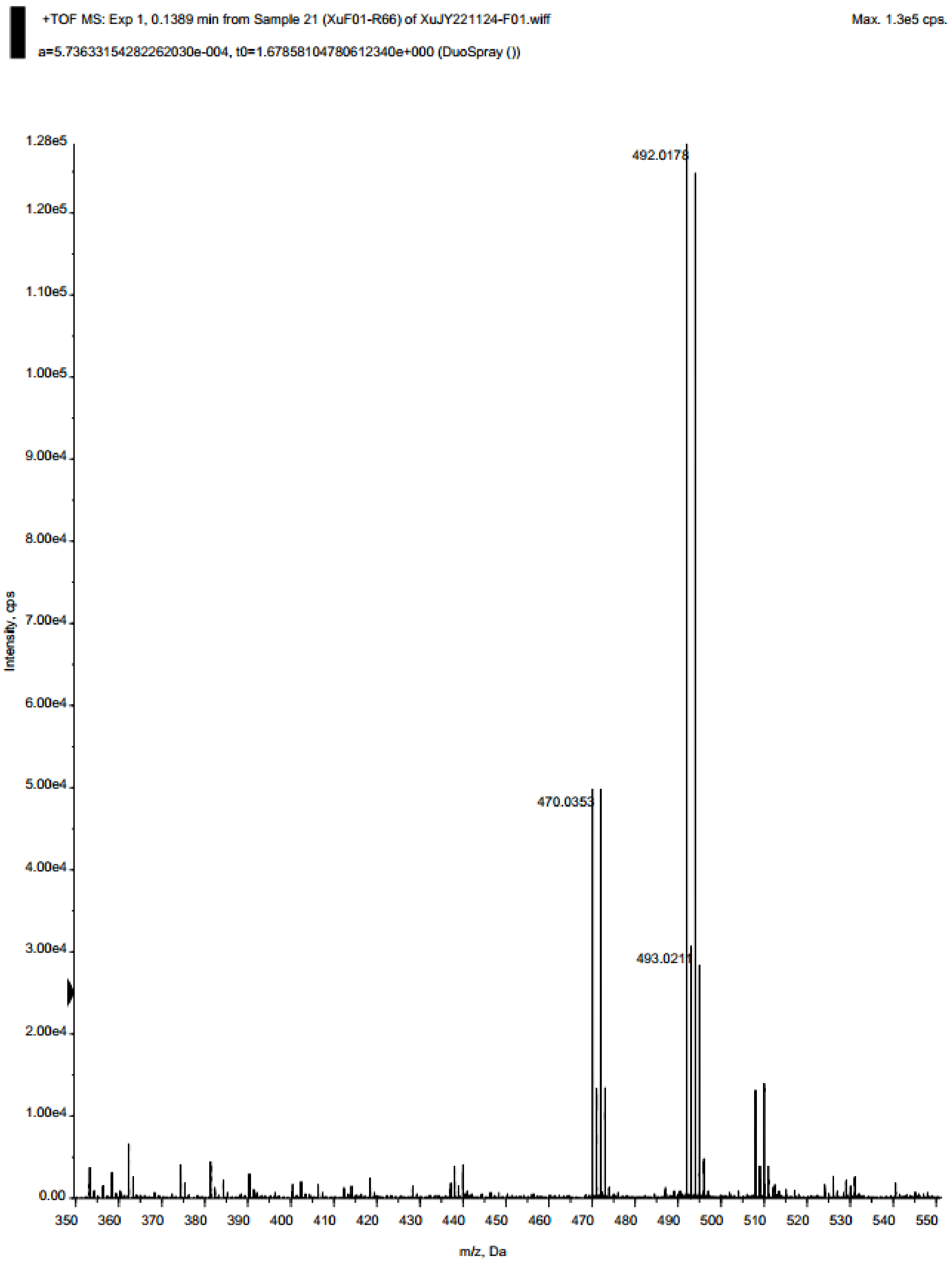

+TOF MS: Exp 1, 0.1389 min from Sample 21 (XuF01-R66) of XuJY221124-F01.wiff
Max. 1.3e5 cps.
a=5.73633154282262030e-004, t0=1.67858104780612340e+000 (DuoSpray ())
492.0178
470.0353
493.0211
Intensity, cps
m/z, Da

Compound **4e**

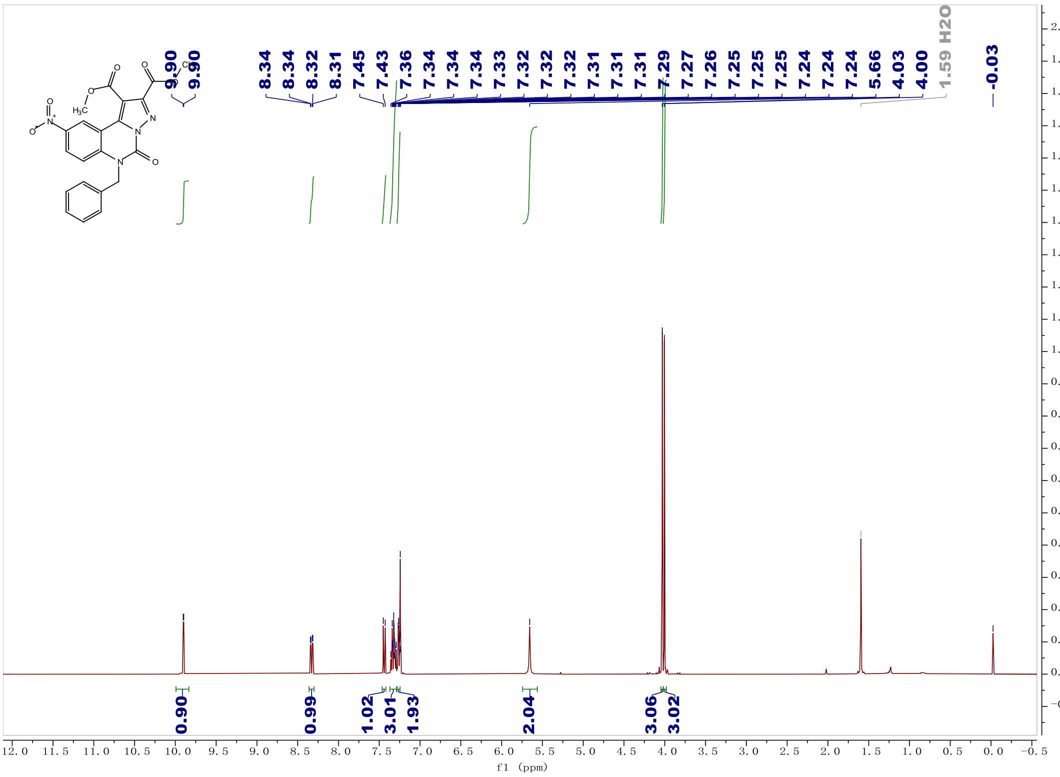

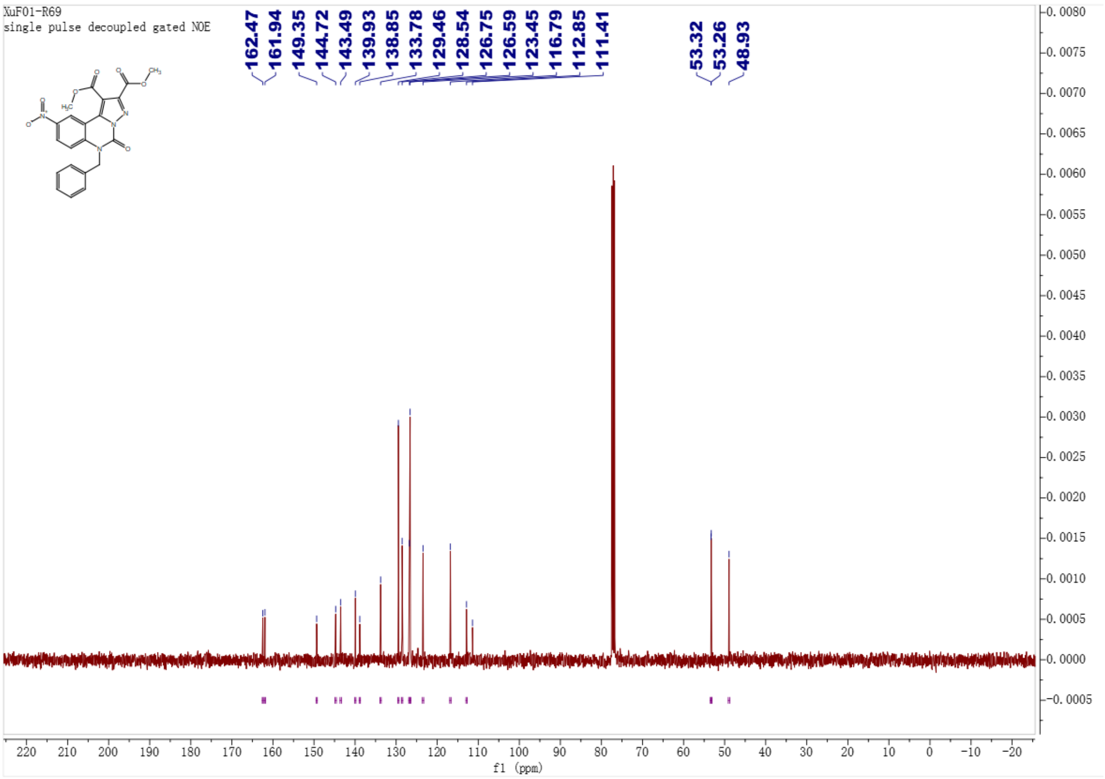

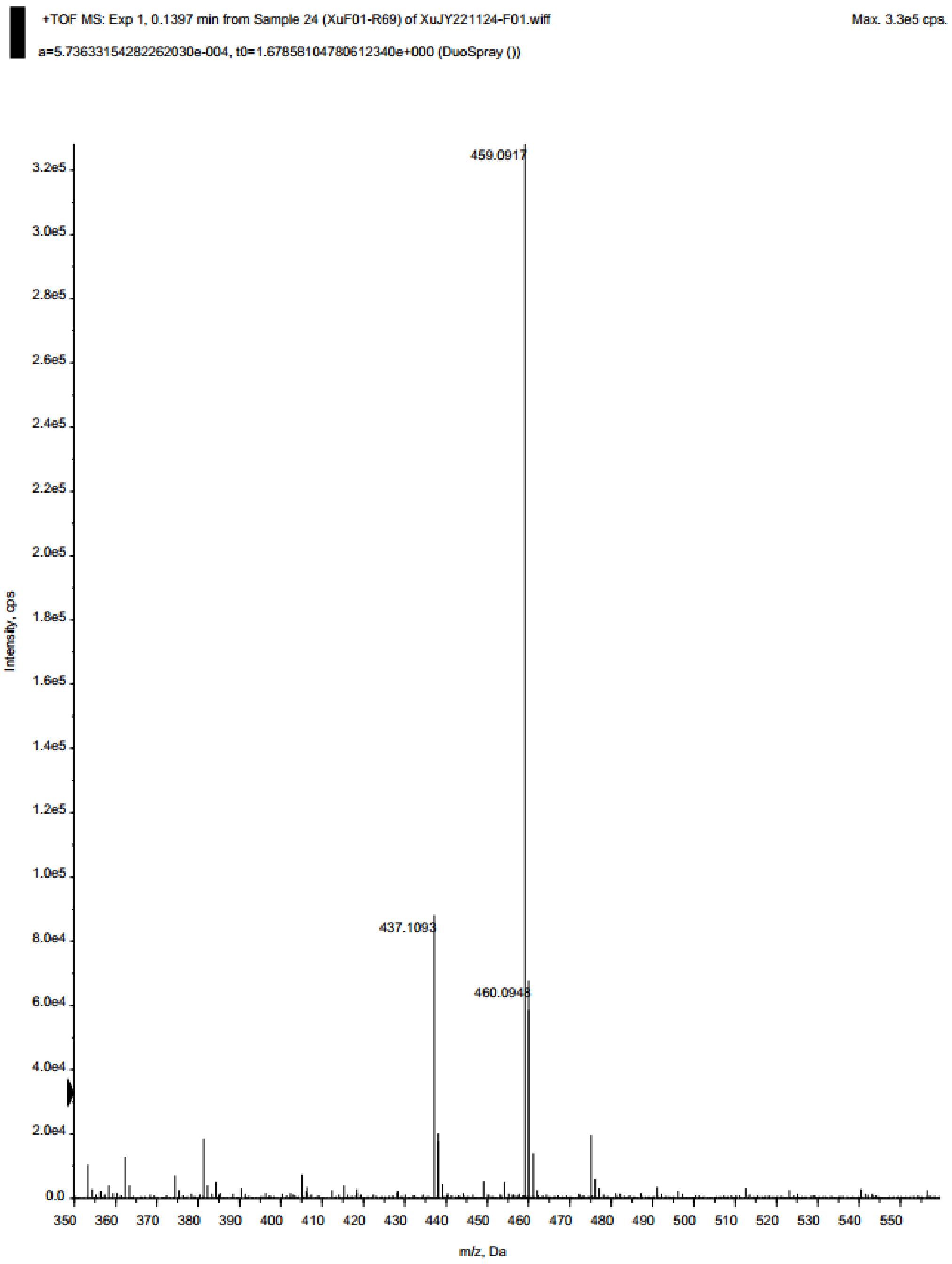

+TOF MS: Exp 1, 0.1397 min from Sample 24 (XuF01-R69) of XuJY221124-F01.wiff
Max. 3.3e5 cps.
a=5.73633154282262030e-004, t0=1.67858104780612340e+000 (DuoSpray ())
459.0917
437.1093
460.0948
Intensity, cps
m/z, Da

Compound **4f**

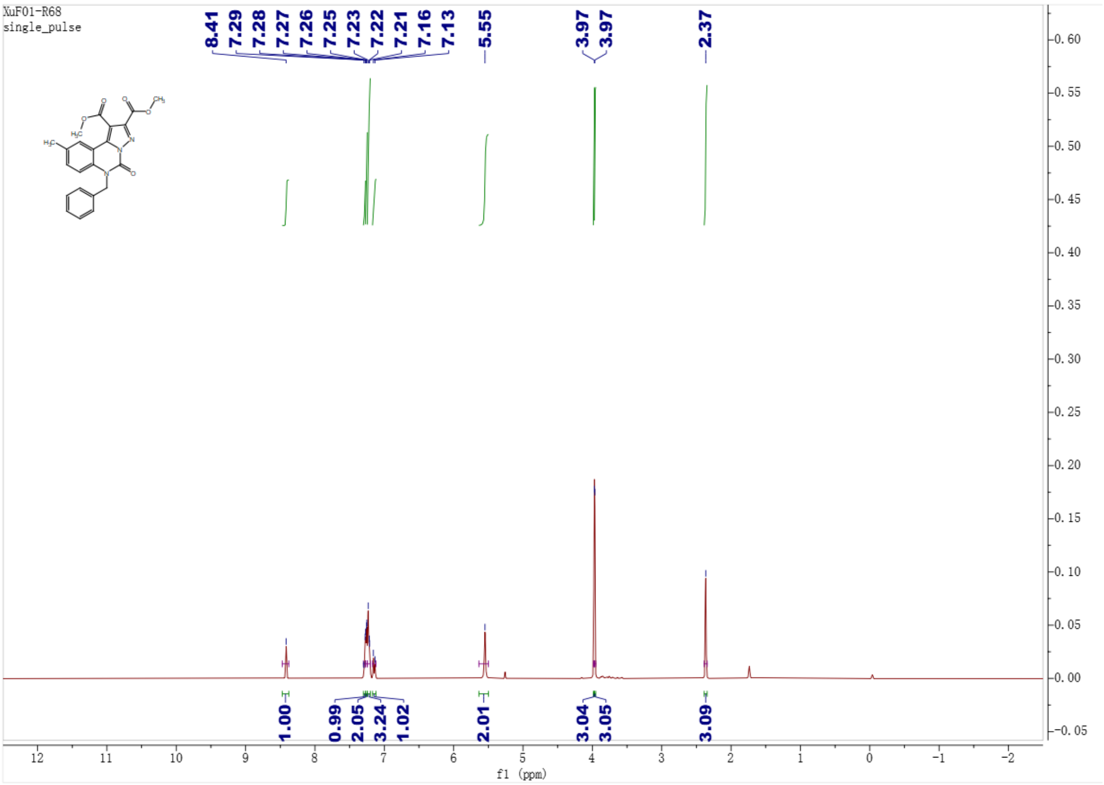

XuF01-R68
single_pulse
8.41
7.29
7.28
7.27
7.26
7.25
7.23
7.22
7.21
7.16
7.13
5.55
3.97
3.97
2.37
1.00
0.99
2.05
3.24
1.02
2.01
3.04
3.05
3.09
f1 (ppm)

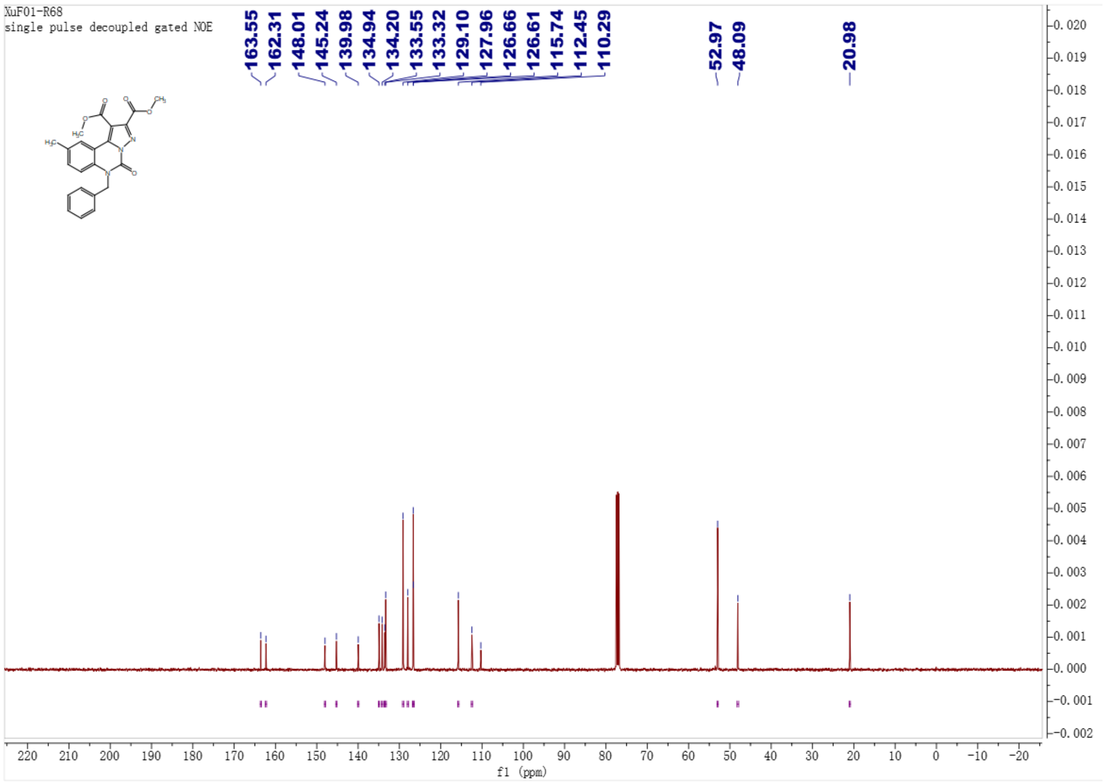

XuF01-R68
single pulse decoupled gated NOE
163.55
162.31
148.01
145.24
139.98
134.94
134.20
133.55
133.32
129.10
127.96
126.66
126.61
115.74
112.45
110.29
52.97
48.09
20.98
f1 (ppm)

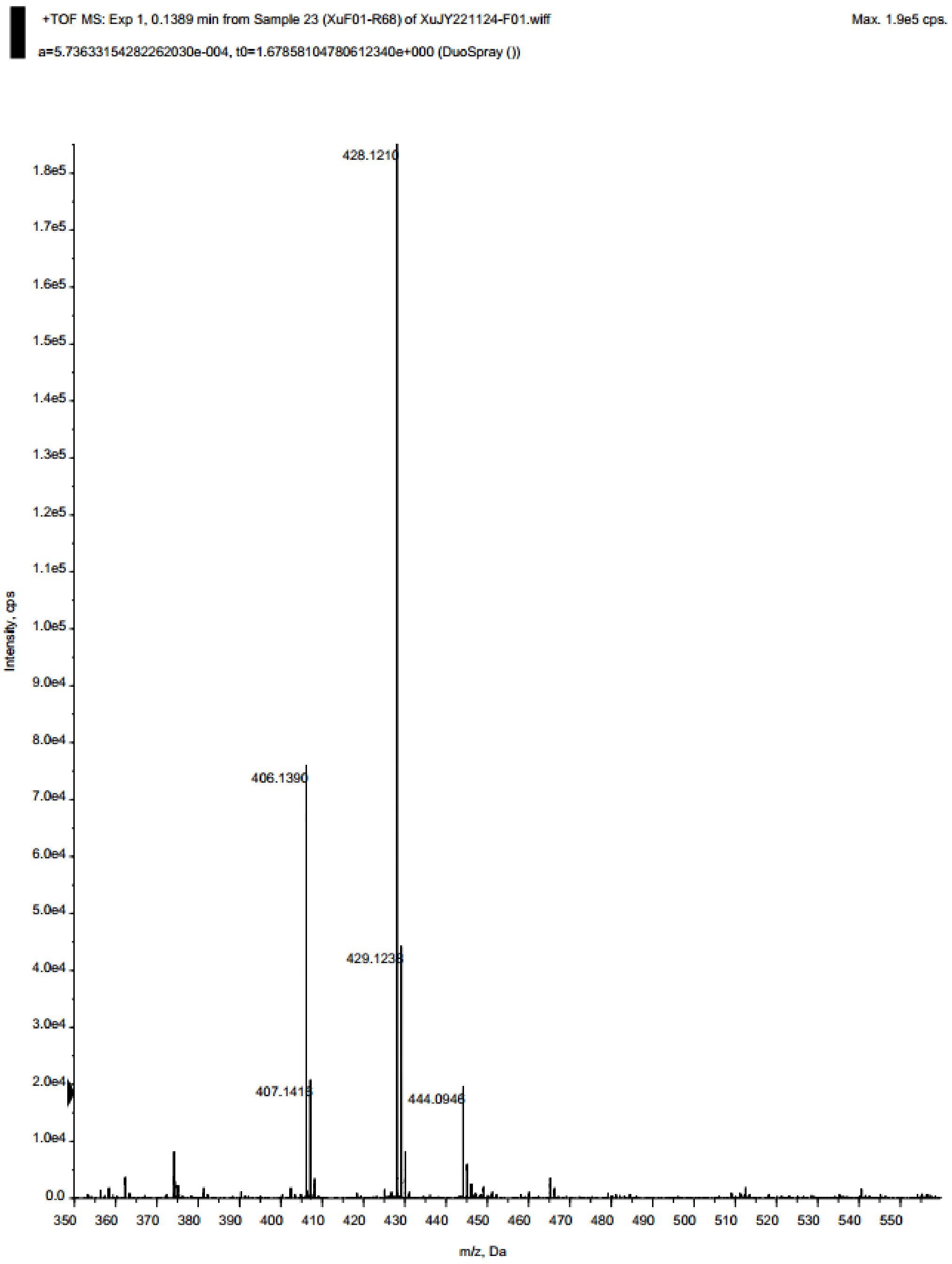
+TOF MS: Exp 1, 0.1389 min from Sample 23 (XuF01-R68) of XuJY221124-F01.wiff
a=5.73633154282262030e-004, t0=1.67858104780612340e+000 (DuoSpray ())
Max. 1.9e5 cps.
428.1210
406.1390
429.1238
407.1418
444.0946
Intensity, cps
m/z, Da

Compound **4g**

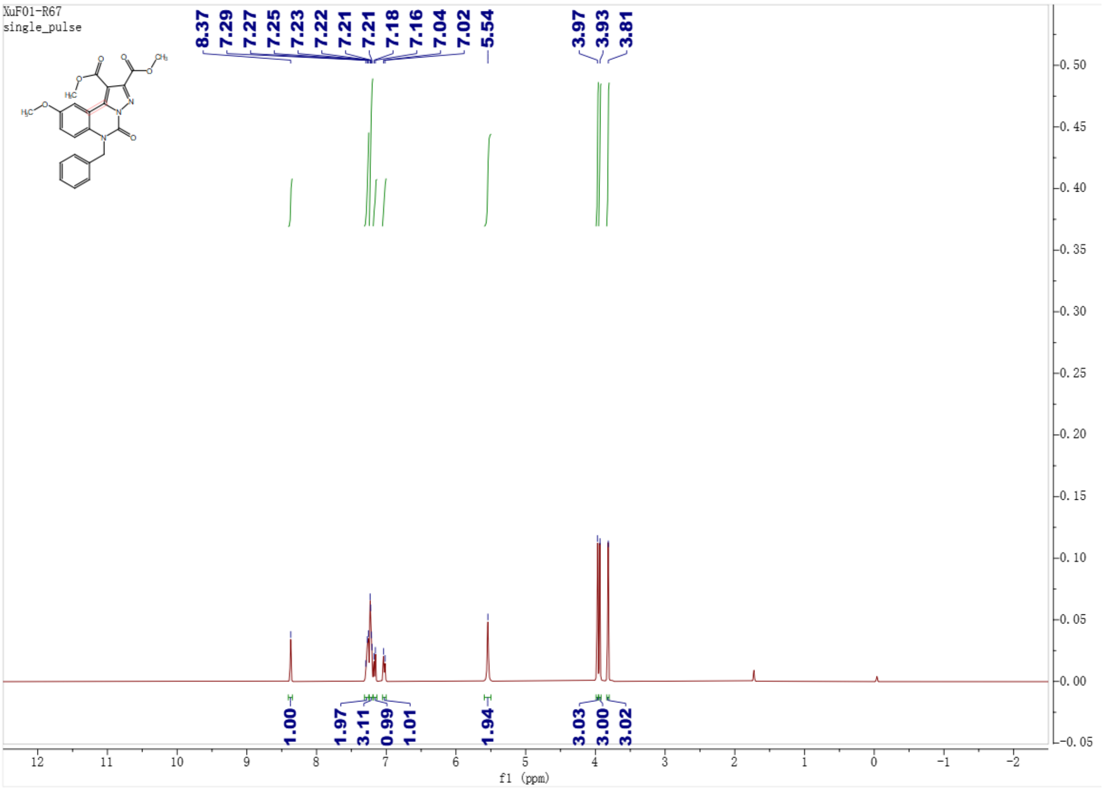

XuF01-R67
single_pulse
8.37
7.29
7.27
7.25
7.23
7.22
7.21
7.21
7.18
7.16
7.04
7.02
5.54
3.97
3.93
3.81
1.00
1.97
3.11
0.99
1.01
1.94
3.03
3.00
3.02
f1 (ppm)

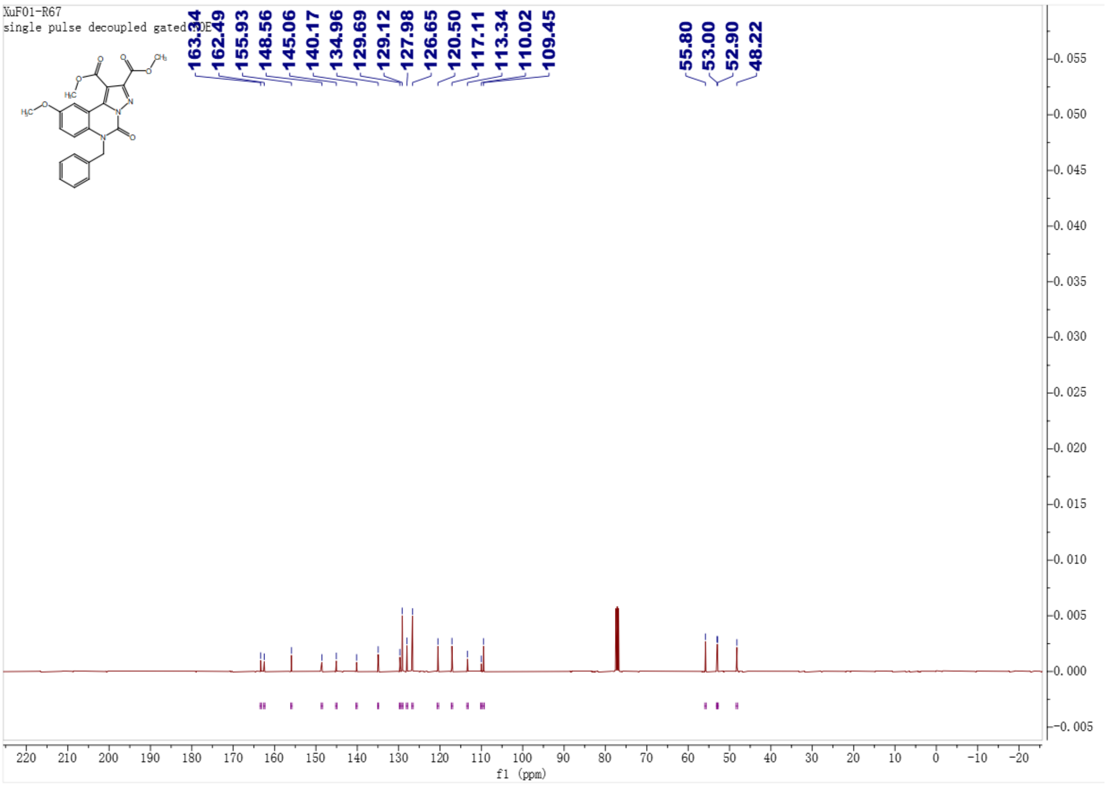

XuF01-R67
single pulse decoupled gated NOE
163.34
162.49
155.93
148.56
145.06
140.17
134.96
129.69
129.12
127.98
126.65
120.50
117.11
113.34
110.02
109.45
55.80
53.00
52.90
48.22
f1 (ppm)

+TOF MS: Exp 1, 0.1373 min from Sample 22 (XuF01-R67) of XuJY221124-F01.wiff Max. 2.3e5 cps.

a=5.73633154282262030e-004, t0=1.67858104780612340e+000 (DuoSpray ())

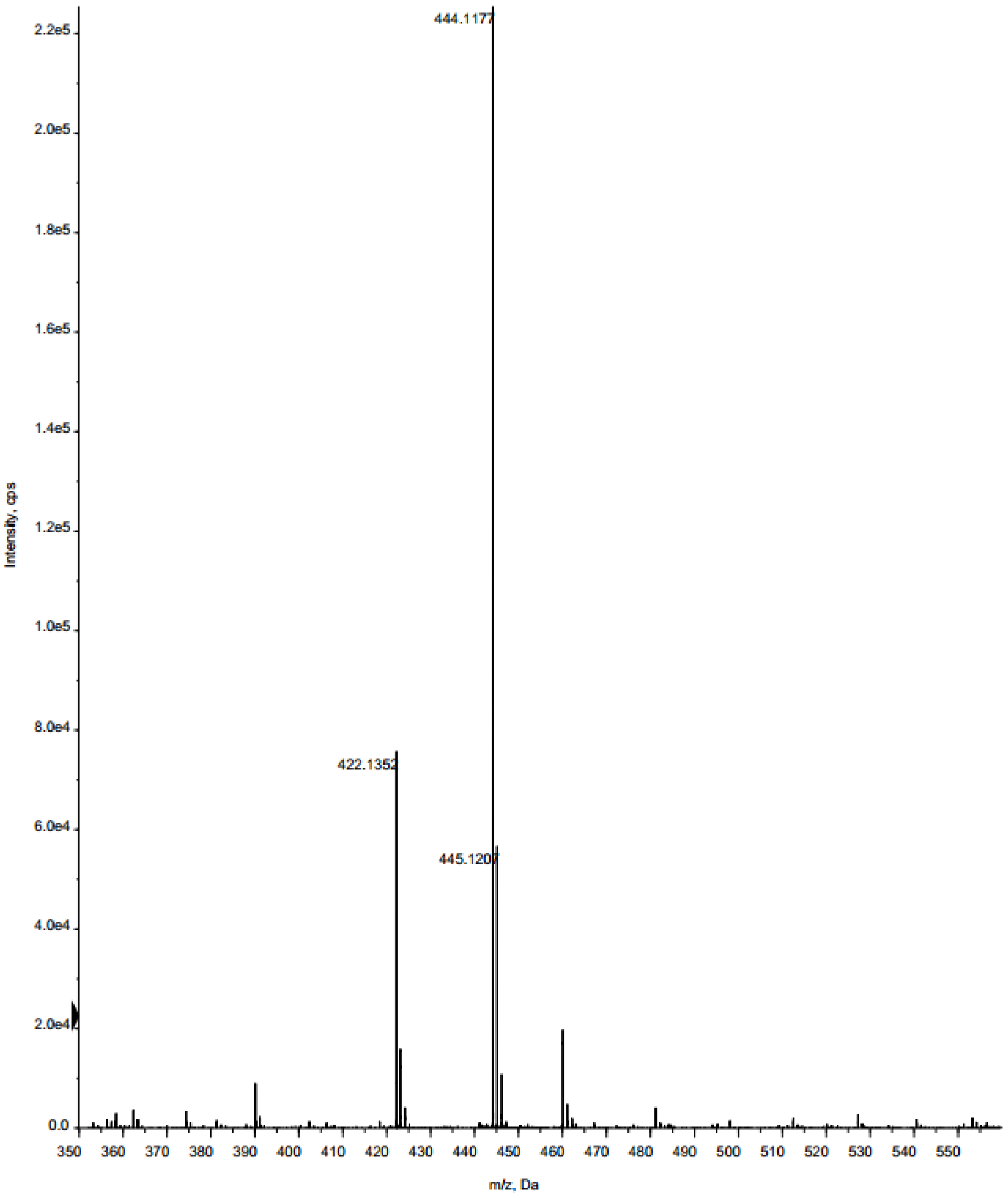

Compound **4h**

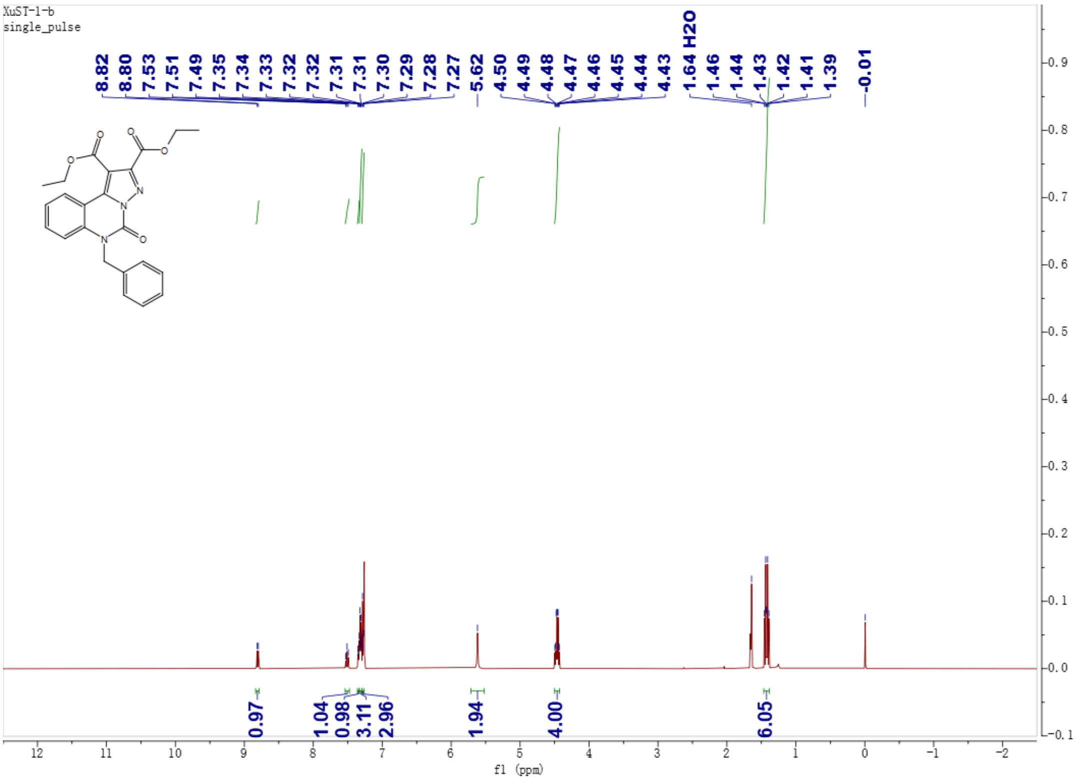
XuST-1-b
single_pulse
8.82 8.80 7.53 7.51 7.49 7.35 7.34 7.33 7.32 7.32 7.31 7.31 7.30 7.29 7.28 7.27 5.62 4.50 4.49 4.48 4.47 4.46 4.45 4.44 4.43 1.64 H2O 1.46 1.44 1.43 1.42 1.41 1.39 -0.01
0.97 1.04 0.98 3.11 2.96 1.94 4.00 6.05
f1 (ppm)

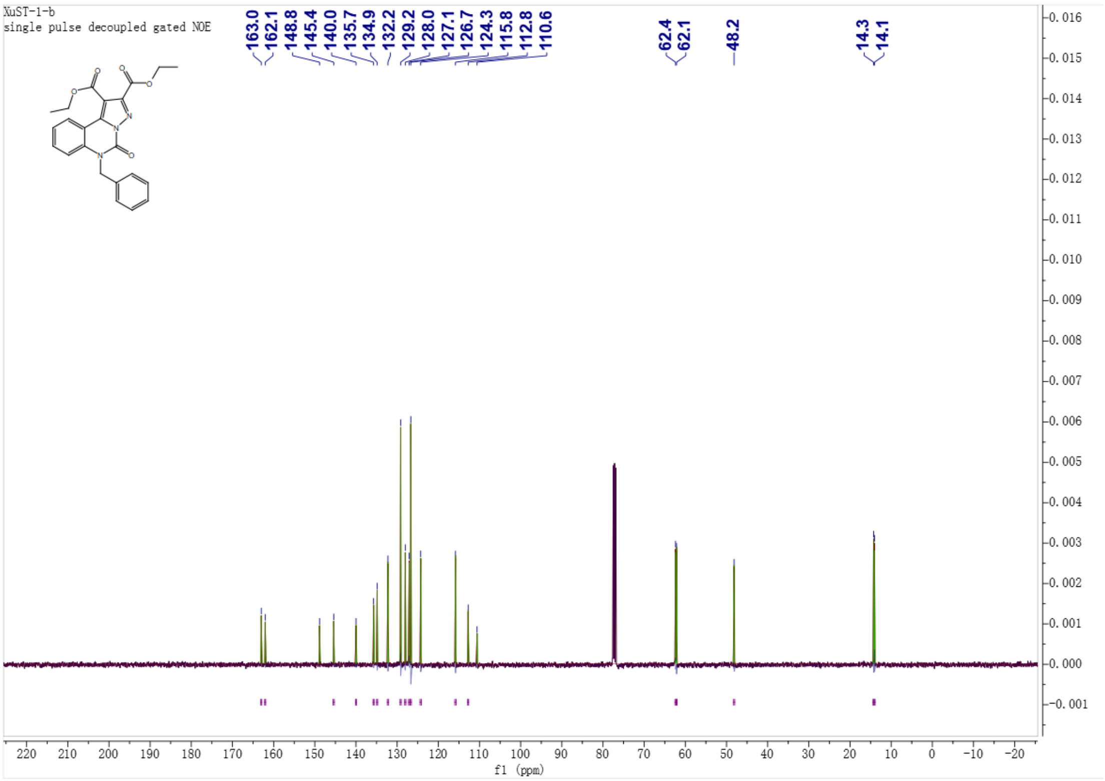
XuST-1-b
single pulse decoupled gated NOE
163.0 162.1 148.8 145.4 140.0 135.7 134.9 132.2 129.2 128.0 127.1 126.7 124.3 115.8 112.8 110.6 62.4 62.1 48.2 14.3 14.1
f1 (ppm)

+TOF MS: Exp 1, 0.1278 min from Sample 1 (XuST-1-b) of XuJY221124-F01.wiff   Max. 5.7e5 cps.

a=5.73633597263515710e-004, t0=1.64052178014956770e+000 (DuoSpray ())

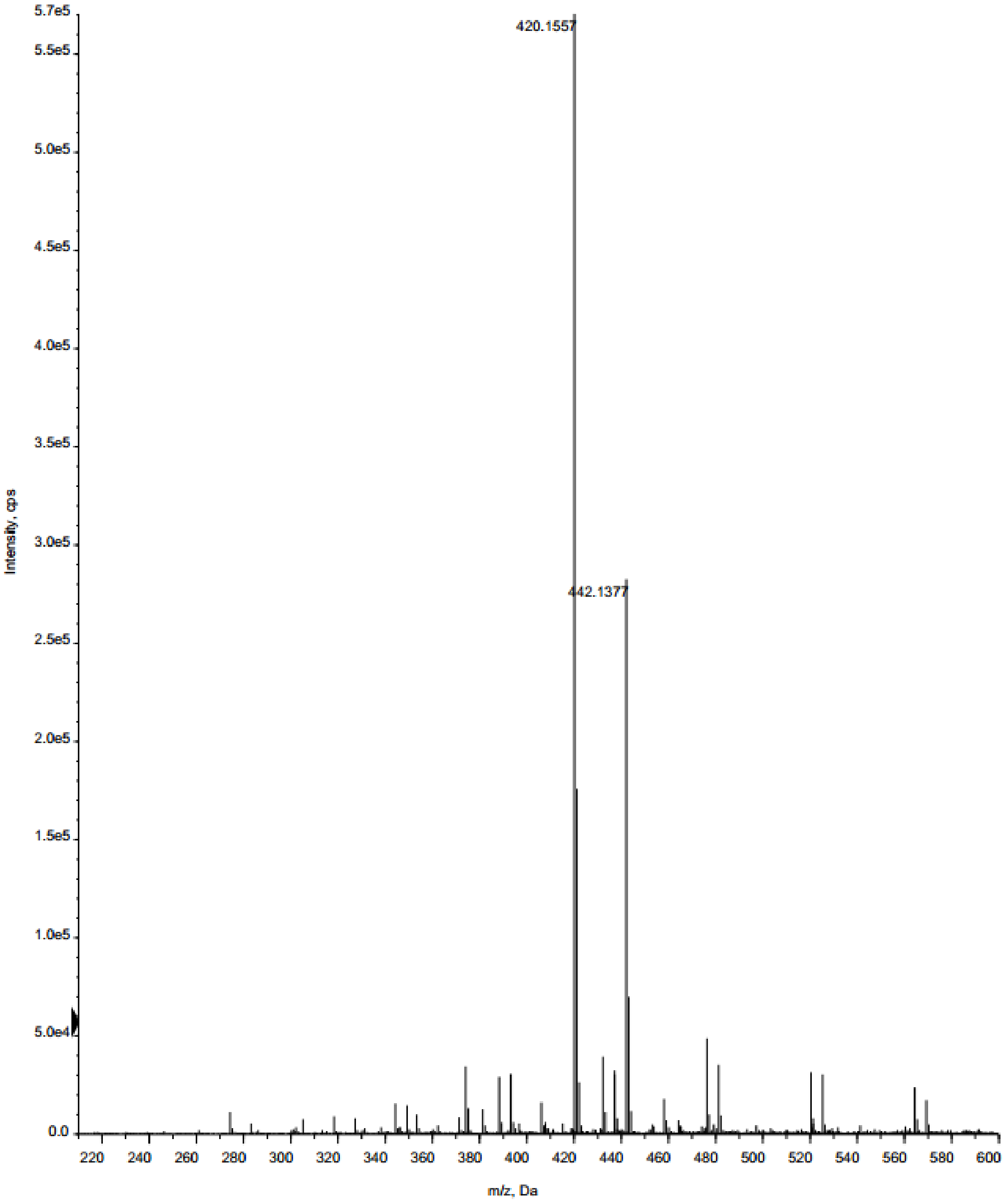

Compound **4i**

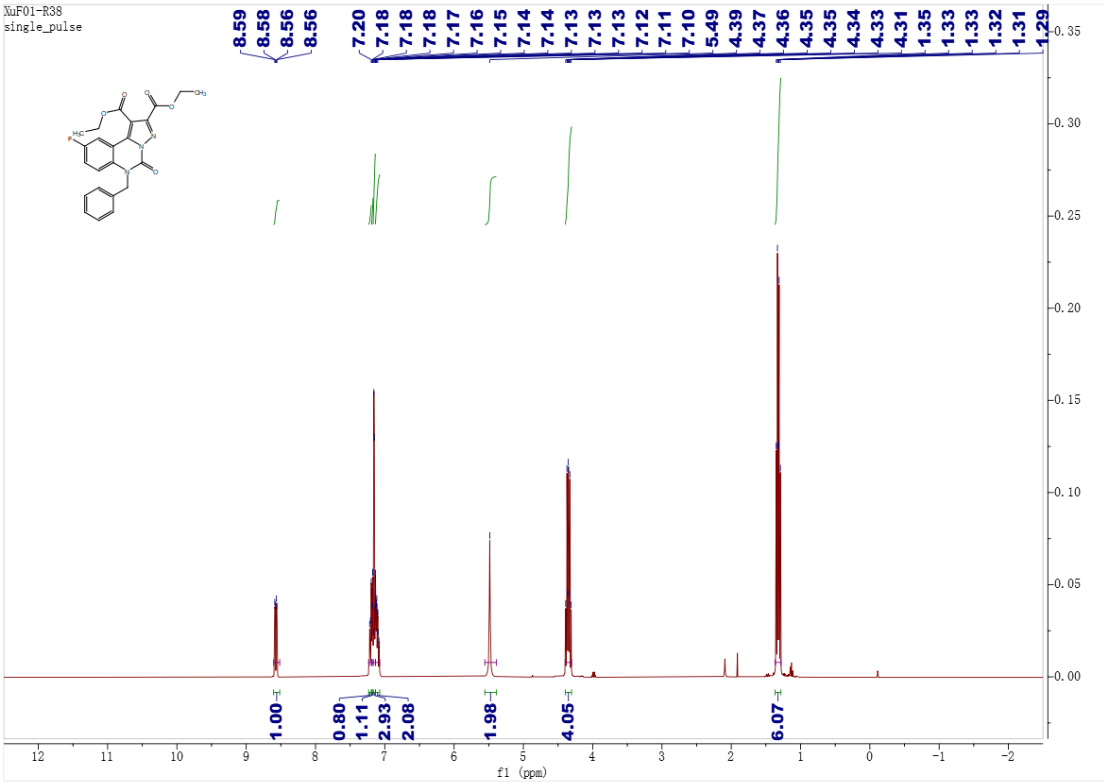
XuF01-R38
single_pulse
8.59
8.58
8.56
8.56
7.20
7.18
7.18
7.18
7.17
7.16
7.15
7.14
7.14
7.13
7.13
7.13
7.12
7.11
7.10
5.49
4.39
4.37
4.36
4.35
4.35
4.34
4.33
4.31
1.35
1.33
1.33
1.32
1.31
1.29
1.00
0.80
1.11
2.93
2.08
1.98
4.05
6.07
f1 (ppm)

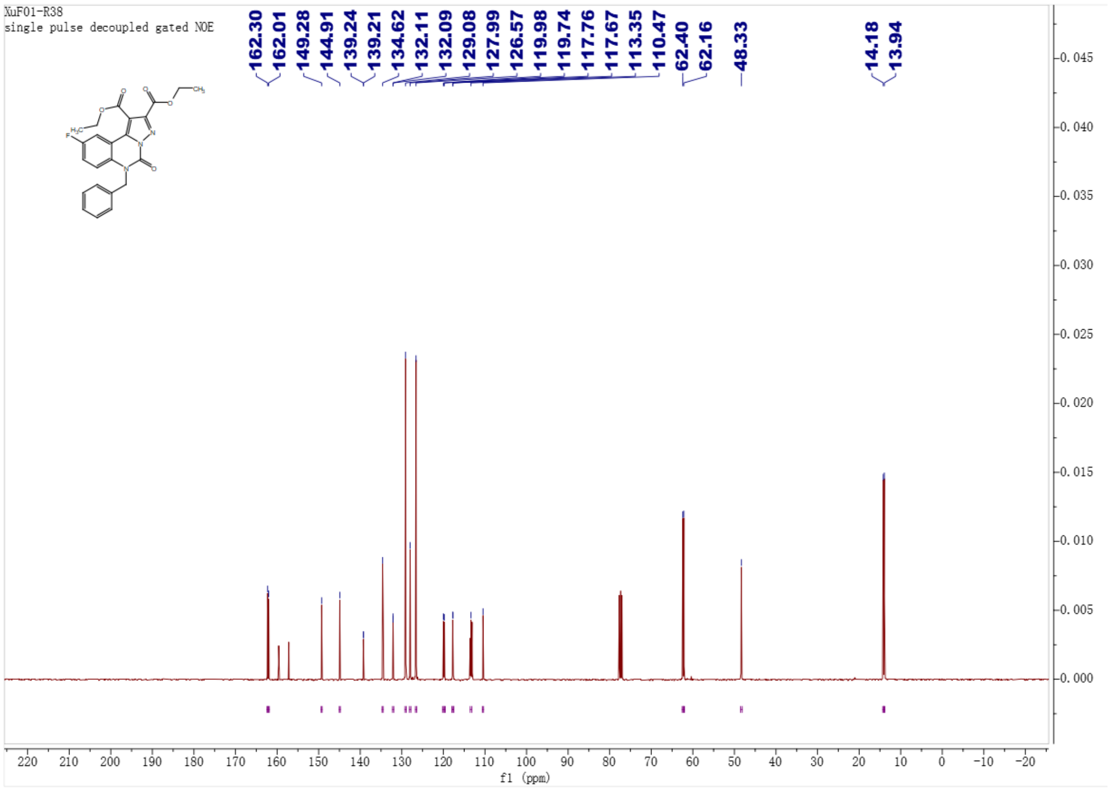
XuF01-R38
single pulse decoupled gated NOE
162.30
162.01
149.28
144.91
139.24
139.21
134.62
132.11
132.09
129.08
127.99
126.57
119.98
119.74
117.76
117.67
113.35
110.47
62.40
62.16
48.33
14.18
13.94
f1 (ppm)

+TOF MS: Exp 1, 0.1140 min from Sample 33 (XuF01-R38) of XuJY221124-F01.wiff Max. 4.8e5 cps.
a=5.73642294333753520e-004, t0=1.69824376062588040e+000 (DuoSpray ())

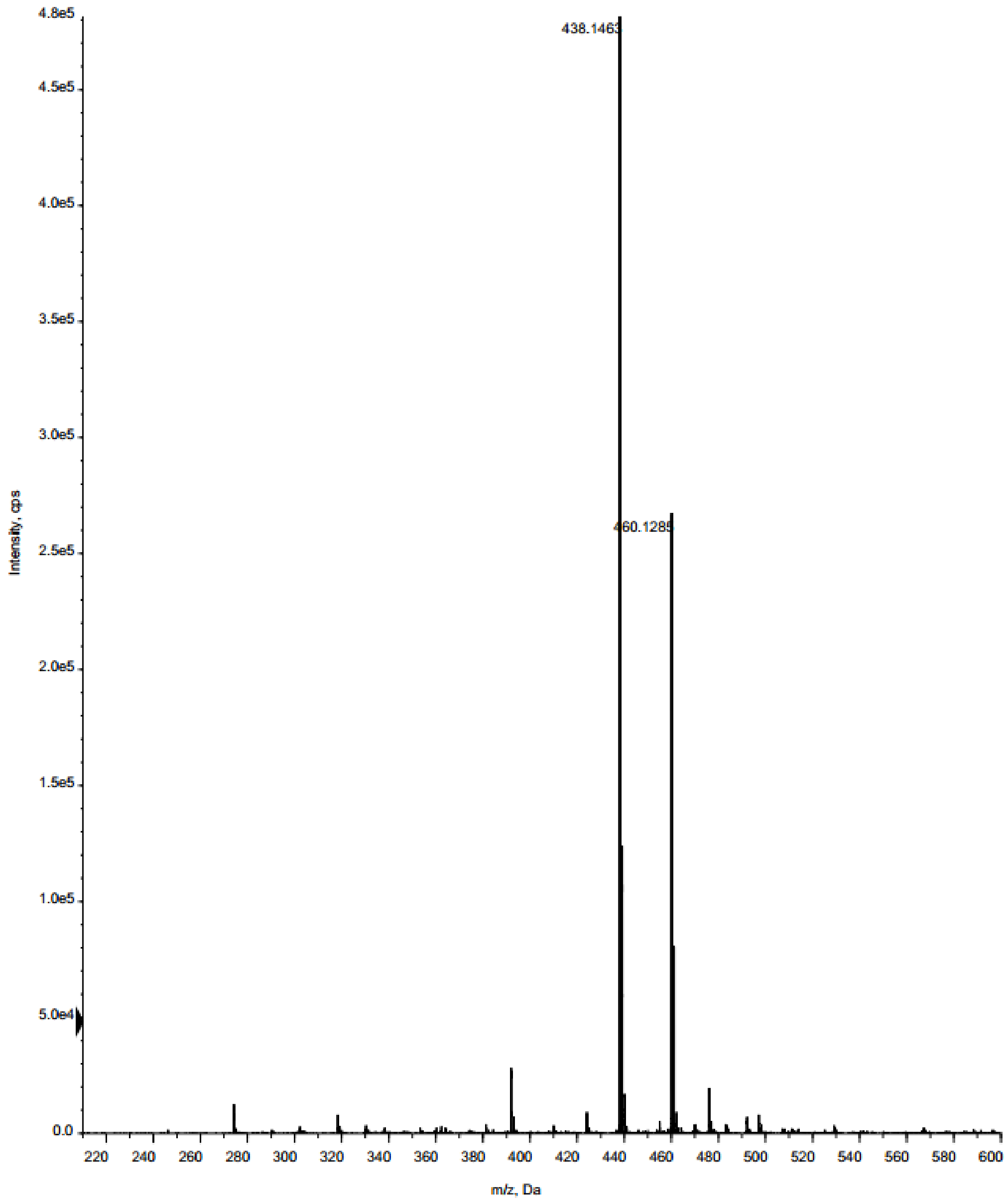

Compound **4j**

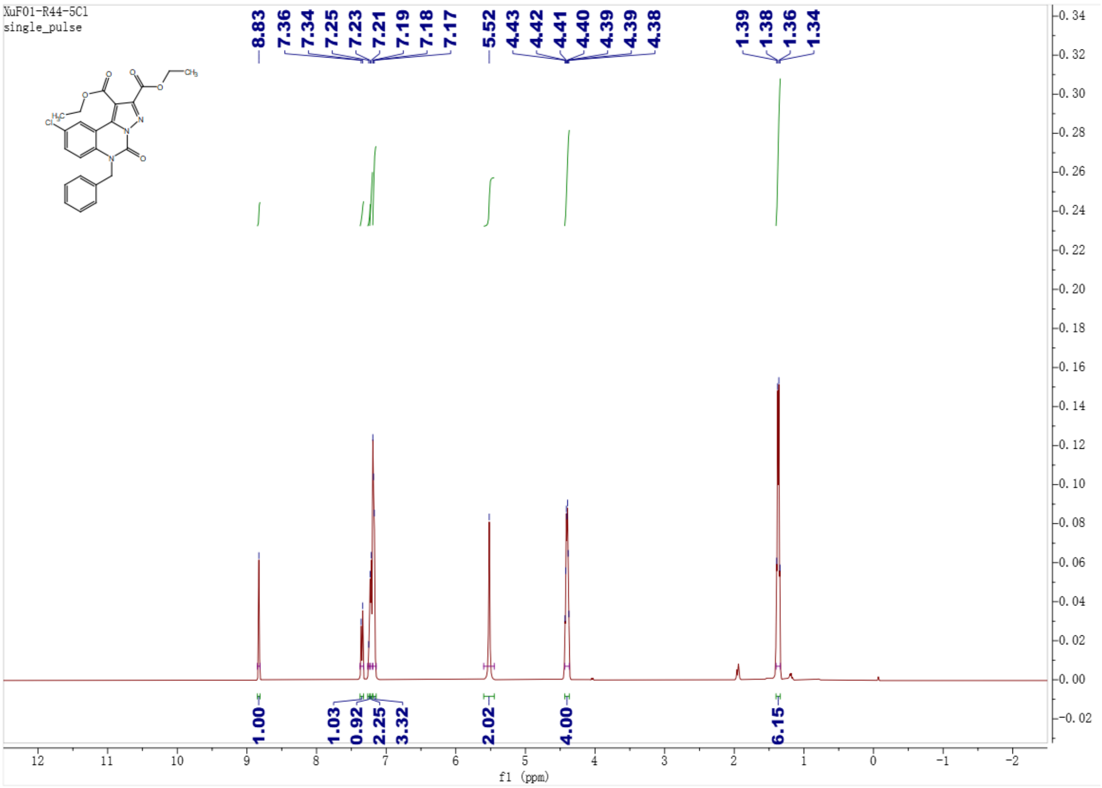
XuF01-R44-5Cl
single_pulse
8.83
7.36
7.34
7.25
7.23
7.21
7.19
7.18
7.17
5.52
4.43
4.42
4.41
4.40
4.39
4.39
4.38
1.39
1.38
1.36
1.34
1.00
1.03
0.92
2.25
3.32
2.02
4.00
6.15
f1 (ppm)

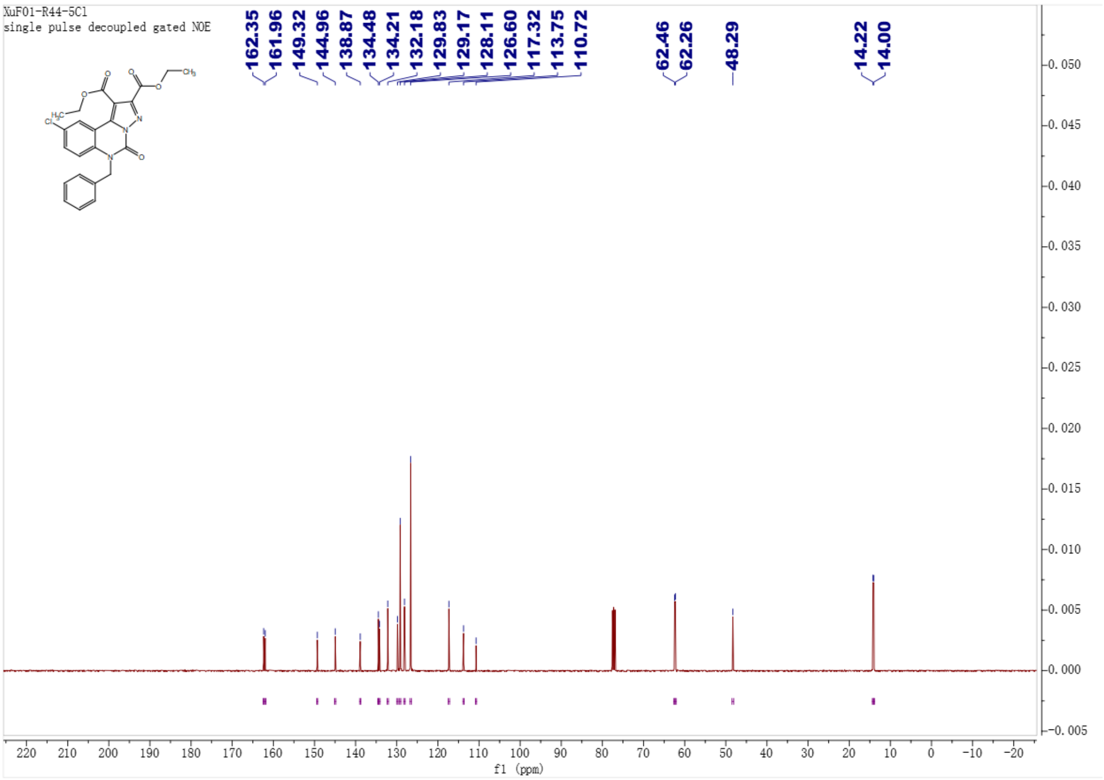
XuF01-R44-5Cl
single pulse decoupled gated NOE
162.35
161.96
149.32
144.96
138.87
134.48
134.21
132.18
129.83
129.17
128.11
126.60
117.32
113.75
110.72
62.46
62.26
48.29
14.22
14.00
f1 (ppm)

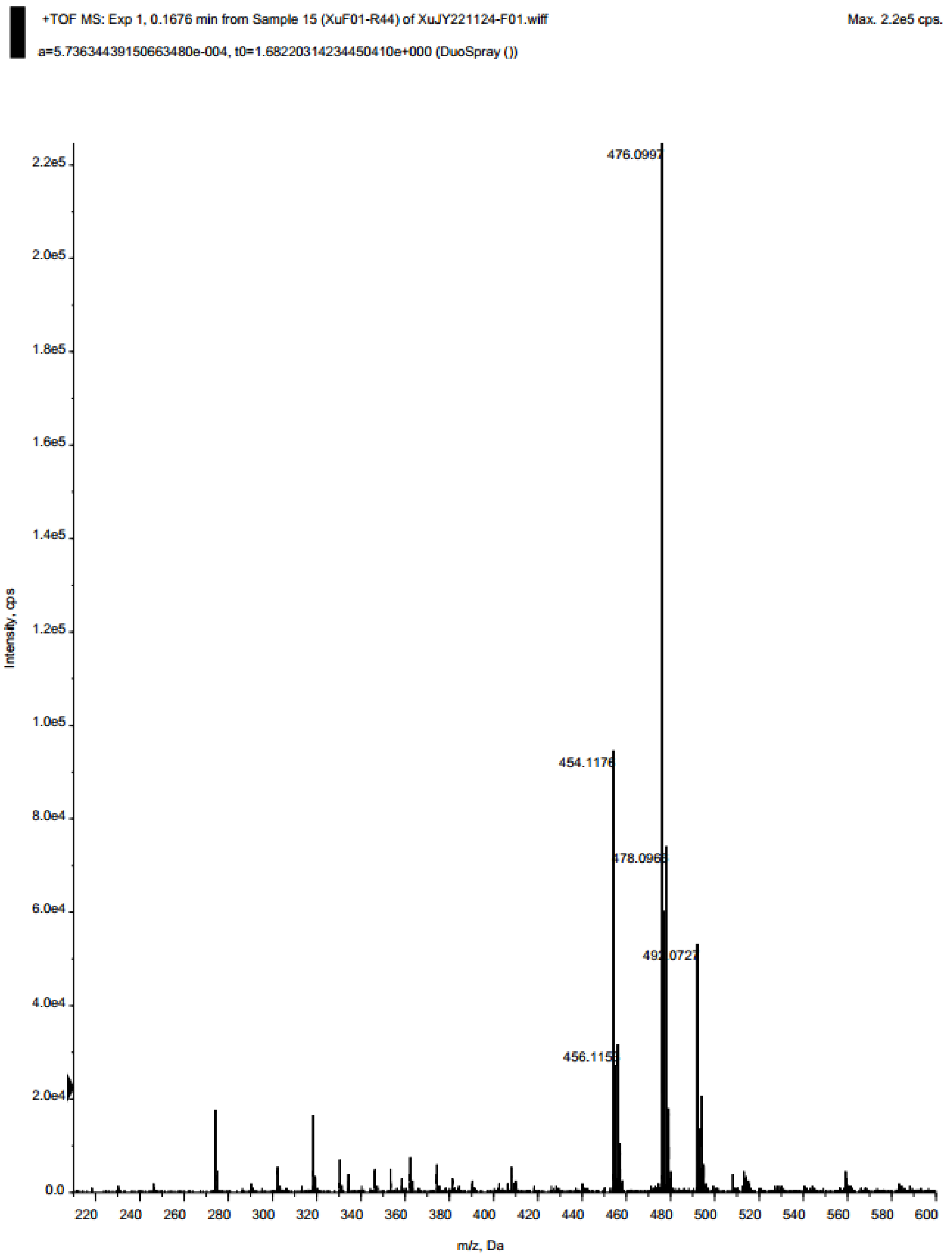
+TOF MS: Exp 1, 0.1676 min from Sample 15 (XuF01-R44) of XuJY221124-F01.wiff
Max. 2.2e5 cps.
a=5.73634439150663480e-004, t0=1.68220314234450410e+000 (DuoSpray ())
476.0997
454.1176
Intensity, cps
m/z, Da

Compound **4k**

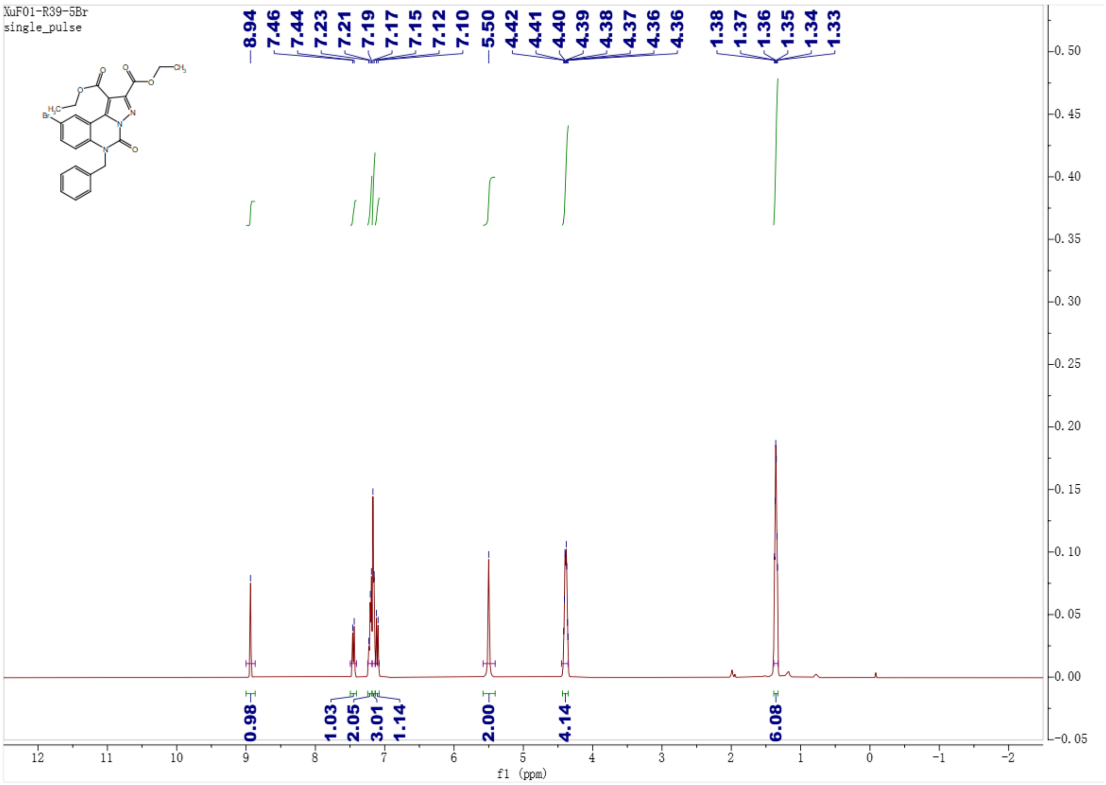

XuF01-R39-5Br
single_pulse
8.94
7.46 7.44 7.23 7.21 7.19 7.17 7.15 7.12 7.10
5.50
4.42 4.41 4.40 4.39 4.38 4.37 4.36 4.36
1.38 1.37 1.36 1.35 1.34 1.33
0.98 1.03 2.05 3.01 1.14 2.00 4.14 6.08
f1 (ppm)

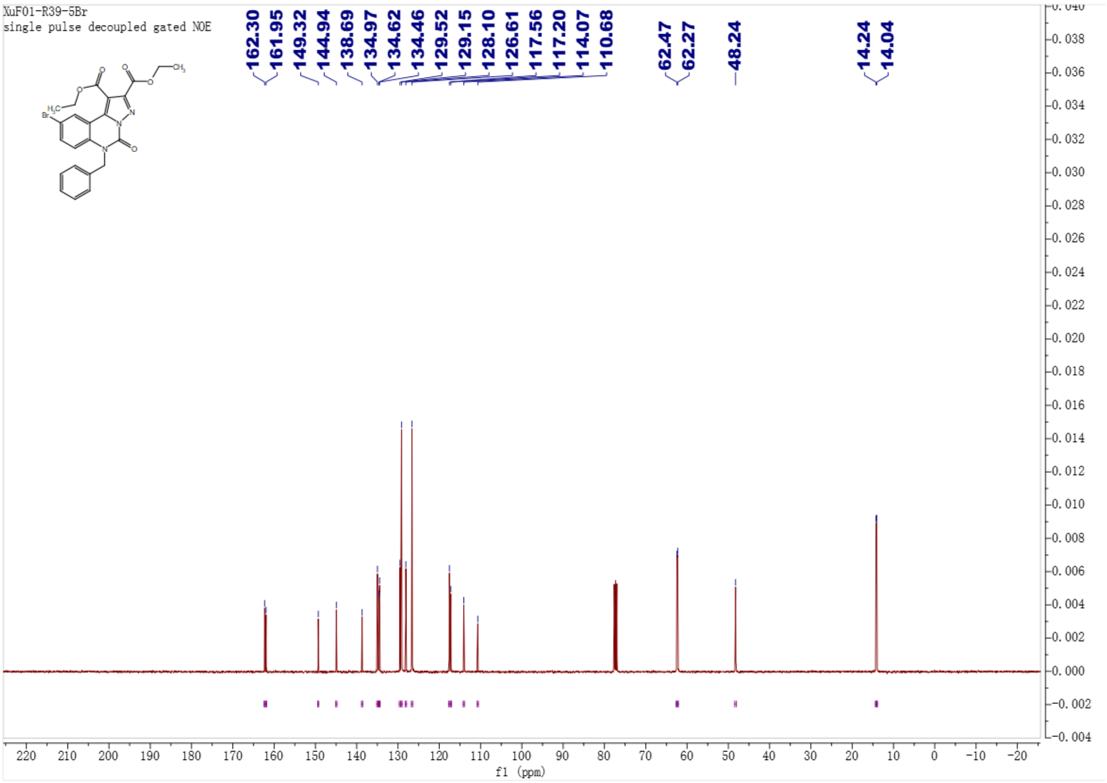

XuF01-R39-5Br
single pulse decoupled gated NOE
162.30 161.95 149.32 144.94 138.69 134.97 134.62 134.46 129.52 129.15 128.10 126.61 117.56 117.20 114.07 110.68
62.47 62.27
48.24
14.24 14.04
f1 (ppm)

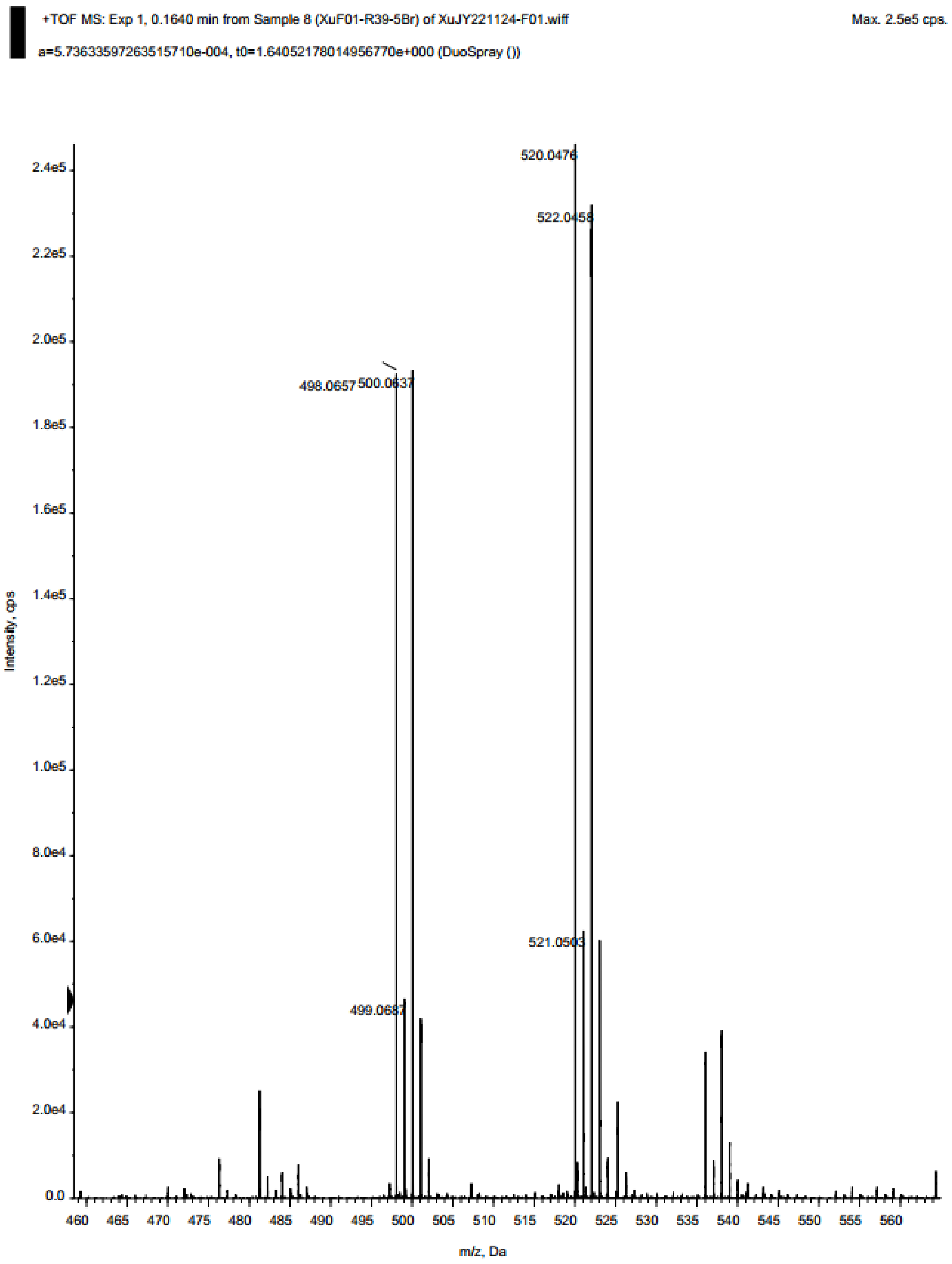
+TOF MS: Exp 1, 0.1640 min from Sample 8 (XuF01-R39-5Br) of XuJY221124-F01.wiff
Max. 2.5e5 cps.
a=5.73633597263515710e-004, t0=1.64052178014956770e+000 (DuoSpray ())
520.0476
522.0458
498.0657
500.0637
521.0503
499.0687
Intensity, cps
m/z, Da

Compound **4l**

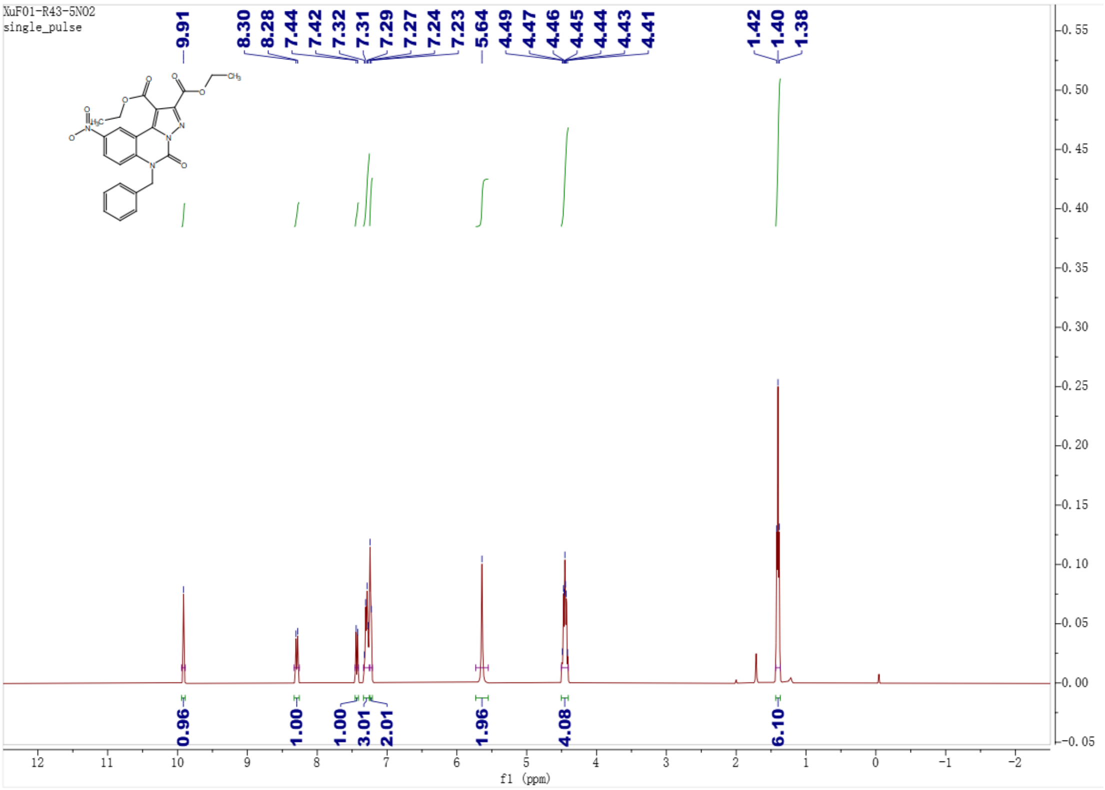
XuF01-R43-5NO2
single_pulse
9.91
8.30
8.28
7.44
7.42
7.32
7.31
7.29
7.27
7.24
7.23
5.64
4.49
4.47
4.46
4.45
4.44
4.43
4.41
1.42
1.40
1.38
0.96
1.00
1.00
3.01
2.01
1.96
4.08
6.10
f1 (ppm)

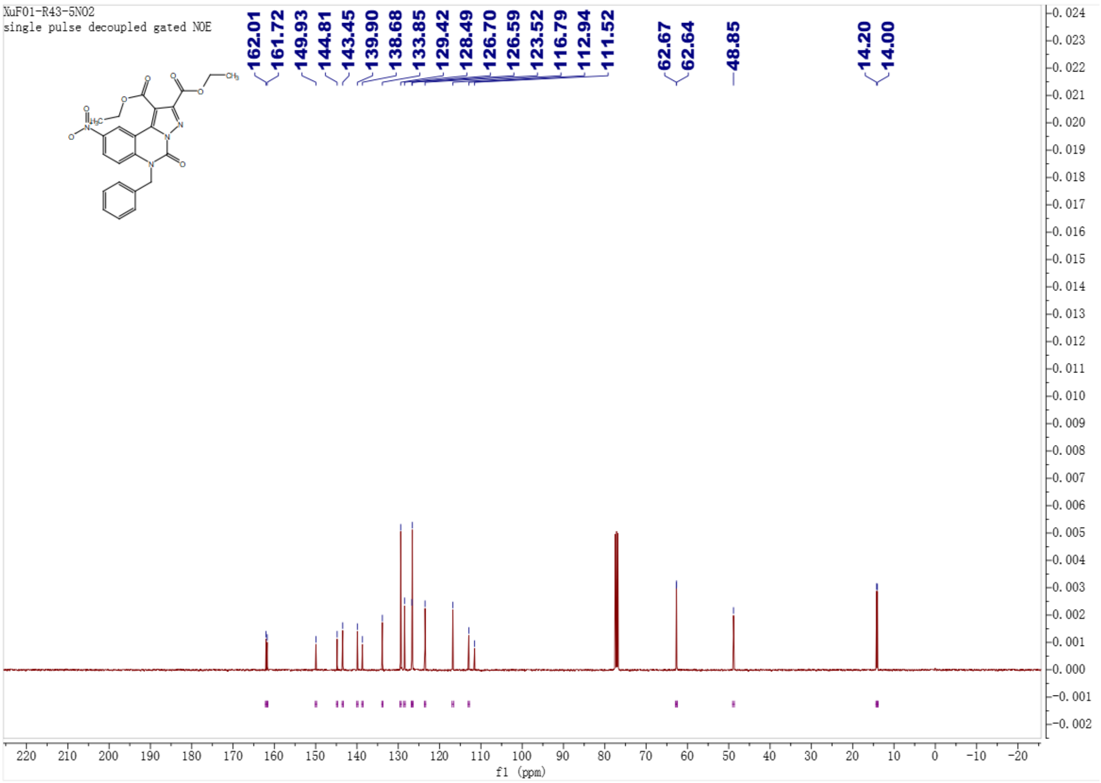
XuF01-R43-5NO2
single pulse decoupled gated NOE
162.01
161.72
149.93
144.81
143.45
139.90
138.68
133.85
129.42
128.49
126.70
126.59
123.52
116.79
112.94
111.52
62.67
62.64
48.85
14.20
14.00
f1 (ppm)

+TOF MS: Exp 1, 0.1139 min from Sample 14 (XuF01-R43) of XuJY221124-F01.wiff   Max. 1.7e5 cps.

a=5.73634439150663480e-004, t0=1.68220314234450410e+000 (DuoSpray ())

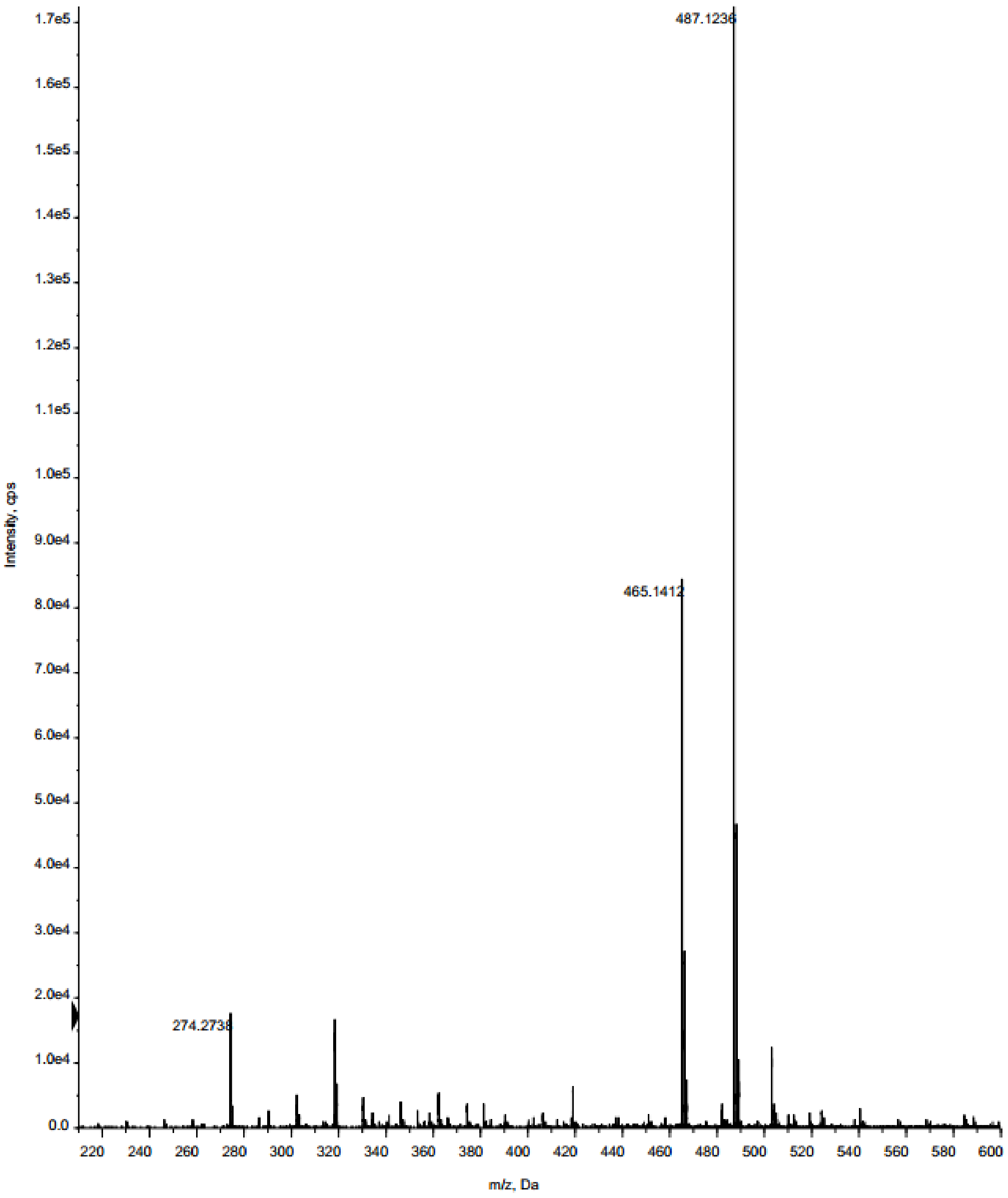

Compound **4m**

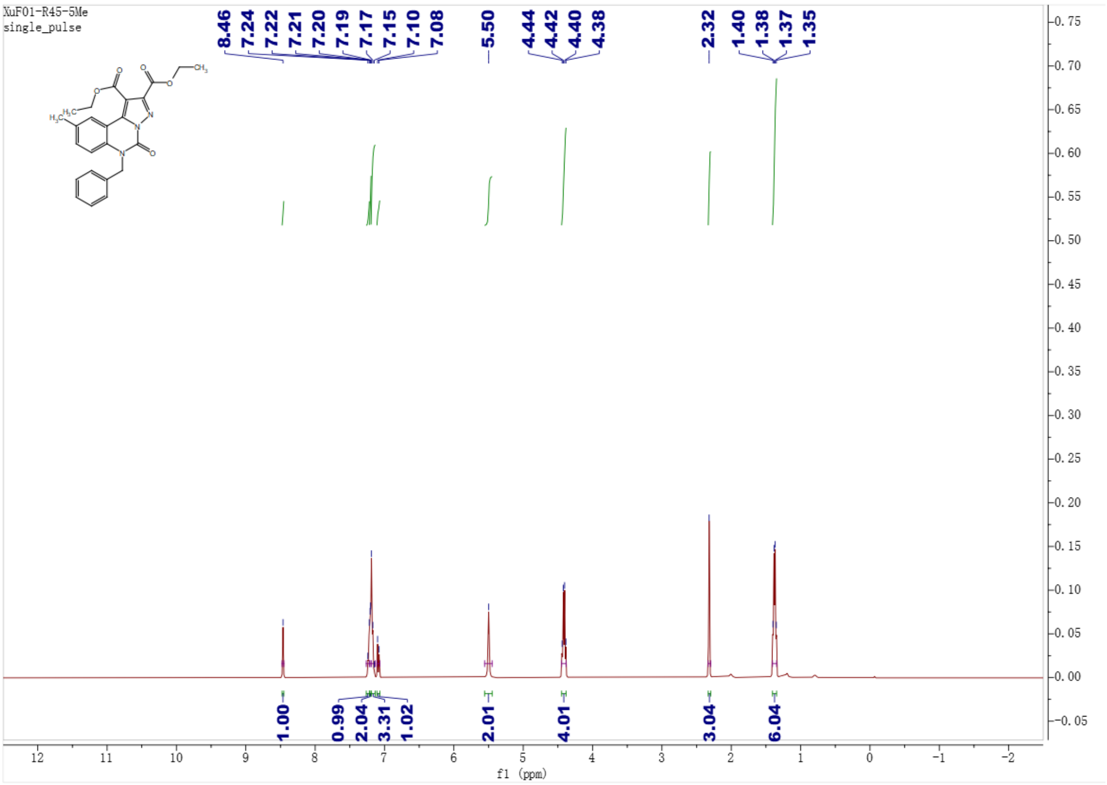
XuF01-R45-5Me
single_pulse
8.46
7.24
7.22
7.21
7.20
7.19
7.17
7.15
7.10
7.08
5.50
4.44
4.42
4.40
4.38
2.32
1.40
1.38
1.37
1.35
1.00
0.99
2.04
3.31
1.02
2.01
4.01
3.04
6.04
f1 (ppm)

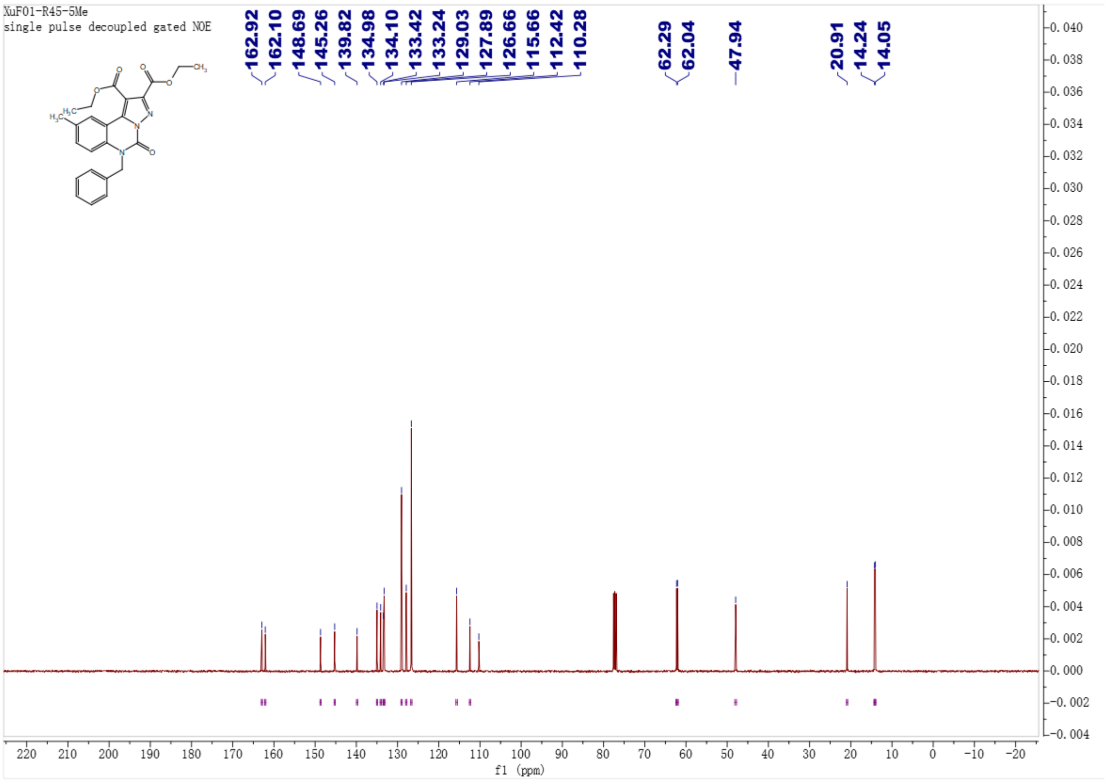
XuF01-R45-5Me
single pulse decoupled gated NOE
162.92
162.10
148.69
145.26
139.82
134.98
134.10
133.42
133.24
129.03
127.89
126.66
115.66
112.42
110.28
62.29
62.04
47.94
20.91
14.24
14.05
f1 (ppm)

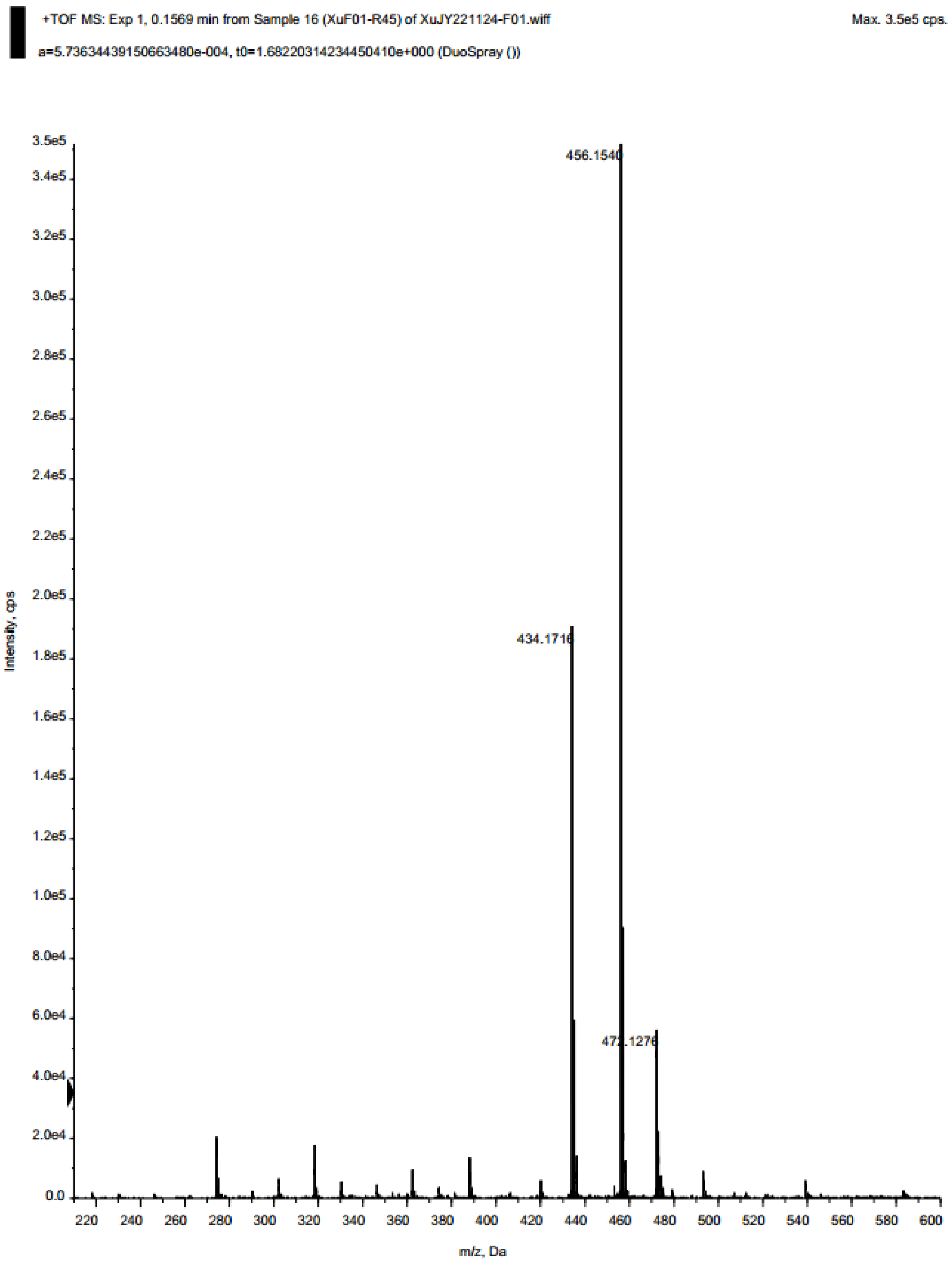
+TOF MS: Exp 1, 0.1569 min from Sample 16 (XuF01-R45) of XuJY221124-F01.wiff
Max. 3.5e5 cps.
a=5.73634439150663480e-004, t0=1.68220314234450410e+000 (DuoSpray ())
456.1540
434.1716
Intensity, cps
m/z, Da

Compound **4n**

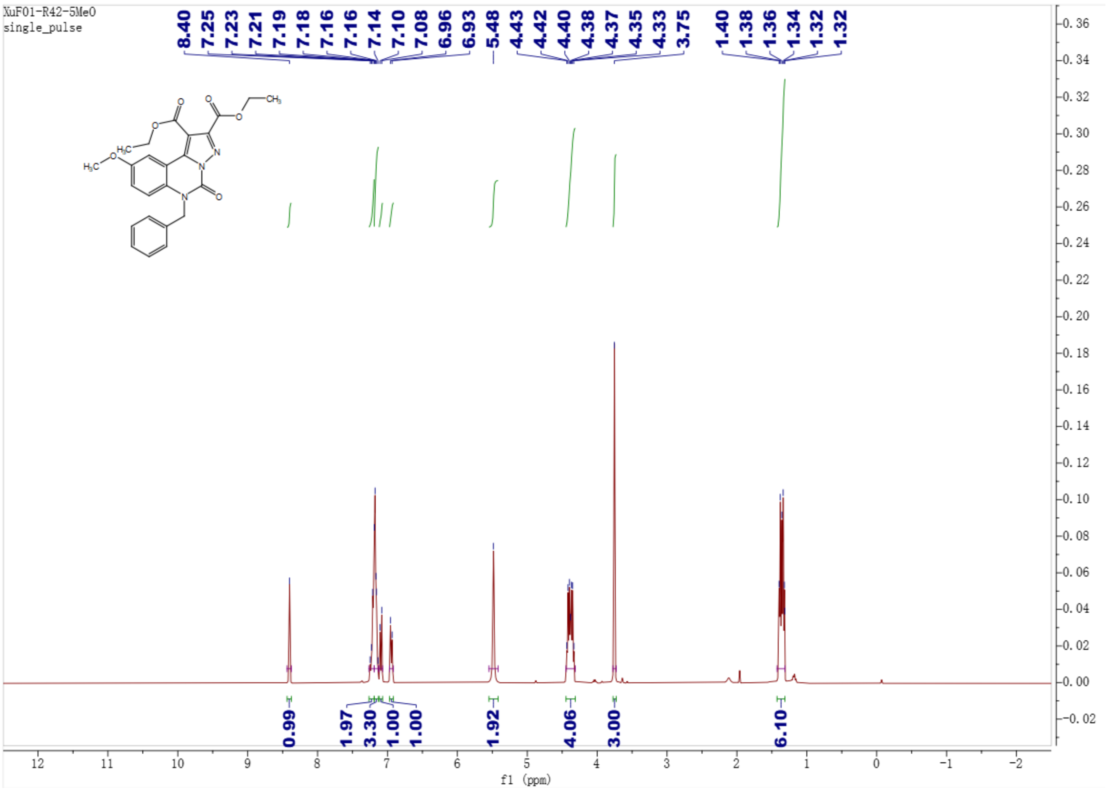
XuF01-R42-5MeO
single_pulse
8.40
7.25
7.23
7.21
7.19
7.18
7.16
7.16
7.14
7.10
7.08
6.96
6.93
5.48
4.43
4.42
4.40
4.38
4.37
4.35
4.33
3.75
1.40
1.38
1.36
1.34
1.32
1.32
0.99
1.97
3.30
1.00
1.00
1.92
4.06
3.00
6.10
f1 (ppm)

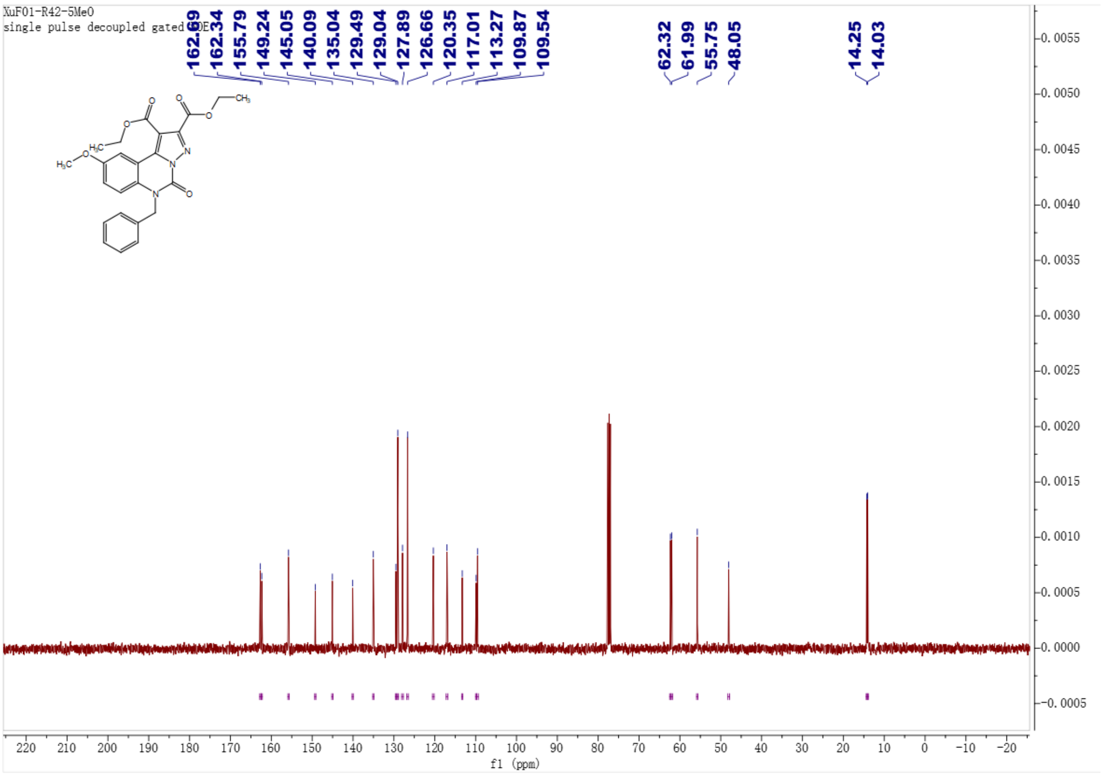
XuF01-R42-5MeO
single pulse decoupled gated NOE
162.69
162.34
155.79
149.24
145.05
140.09
135.04
129.49
129.04
127.89
126.66
120.35
117.01
113.27
109.87
109.54
62.32
61.99
55.75
48.05
14.25
14.03
f1 (ppm)

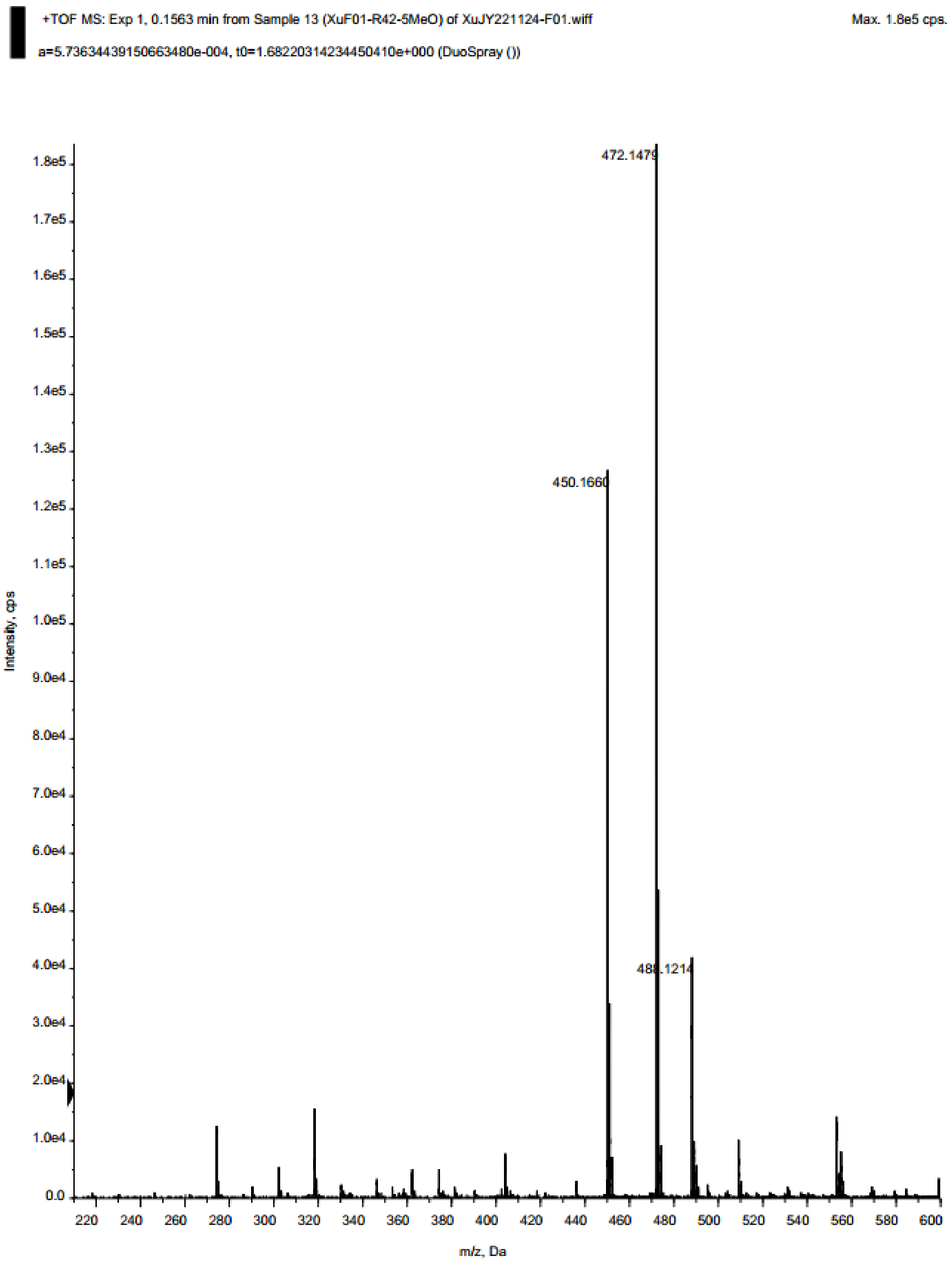
+TOF MS: Exp 1, 0.1563 min from Sample 13 (XuF01-R42-5MeO) of XuJY221124-F01.wiff
a=5.73634439150663480e-004, t0=1.68220314234450410e+000 (DuoSpray ())
Max. 1.8e5 cps.
472.1479
450.1660
Intensity, cps
m/z, Da

Compound **4o**

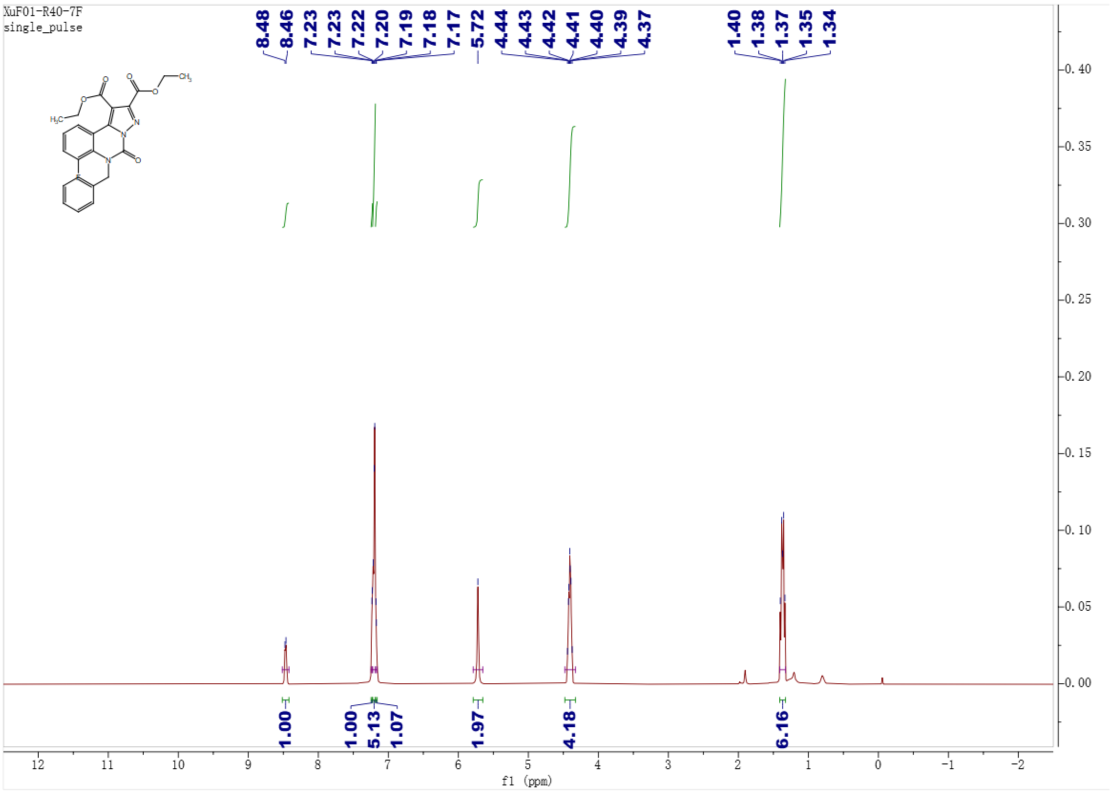
XuF01-R40-7F
single_pulse
f1 (ppm)

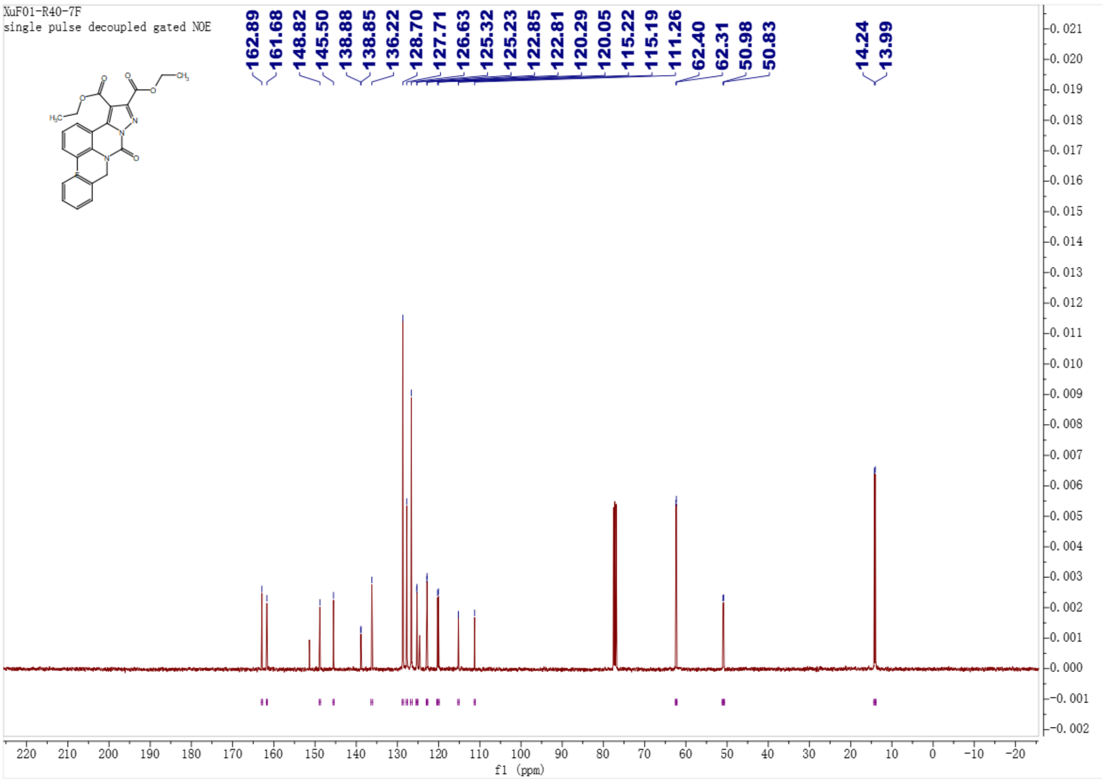
XuF01-R40-7F
single pulse decoupled gated NOE
f1 (ppm)

+TOF MS: Exp 1, 0.1568 min from Sample 11 (XuF01-R40-7F) of XuJY221124-F01.wiff Max. 4.4e5 cps.

a=5.73634439150663480e-004, t0=1.68220314234450410e+000 (DuoSpray ())

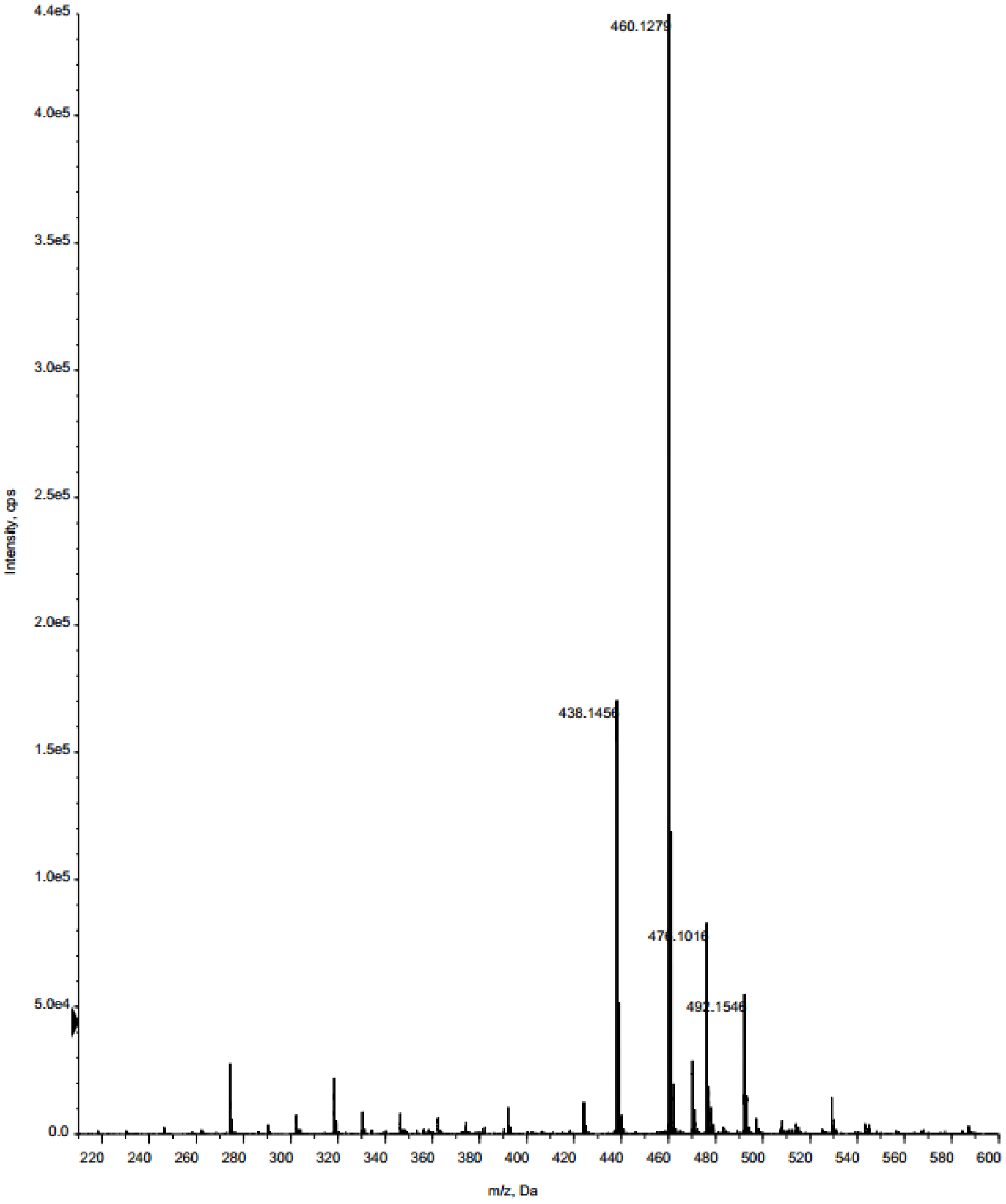

Compound **4p**

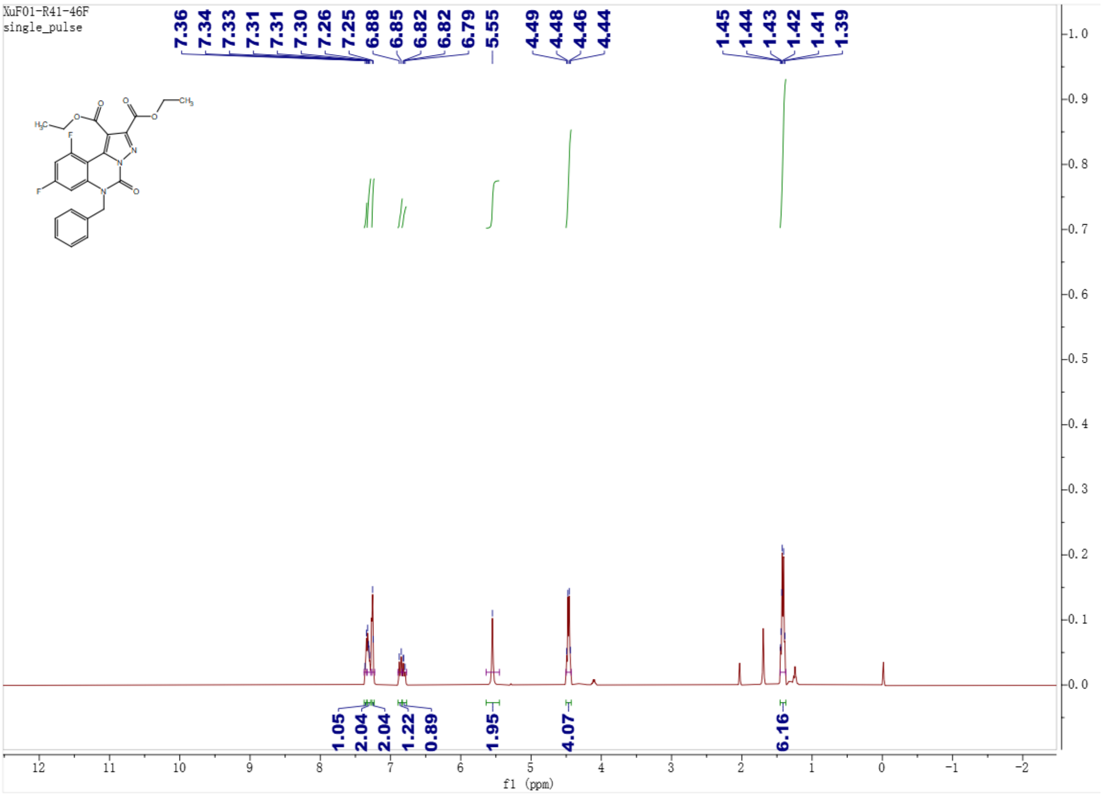
XuF01-R41-46F
single_pulse
7.36
7.34
7.33
7.31
7.31
7.30
7.26
7.25
6.88
6.85
6.82
6.82
6.79
5.55
4.49
4.48
4.46
4.44
1.45
1.44
1.43
1.42
1.41
1.39
1.05
2.04
2.04
1.22
0.89
1.95
4.07
6.16
f1 (ppm)

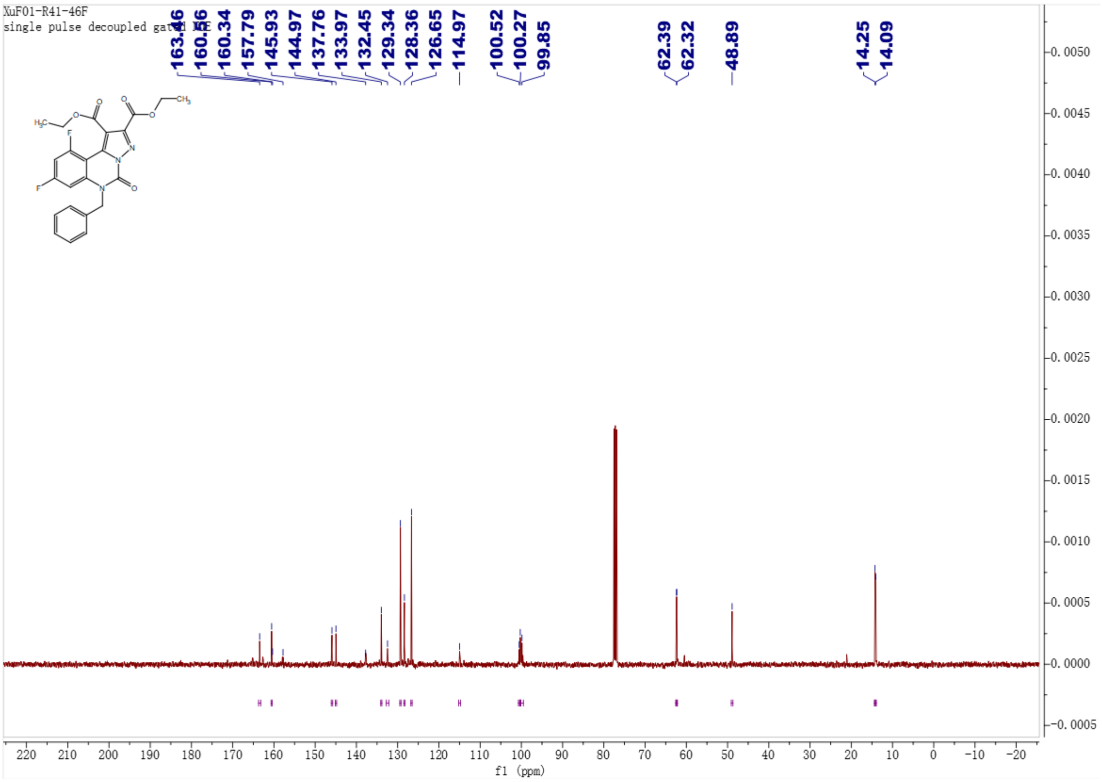
XuF01-R41-46F
163.46
160.34
157.79
145.93
144.97
137.76
133.97
132.45
129.34
128.36
126.65
114.97
100.52
100.27
99.85
62.39
62.32
48.89
14.25
14.09
f1 (ppm)

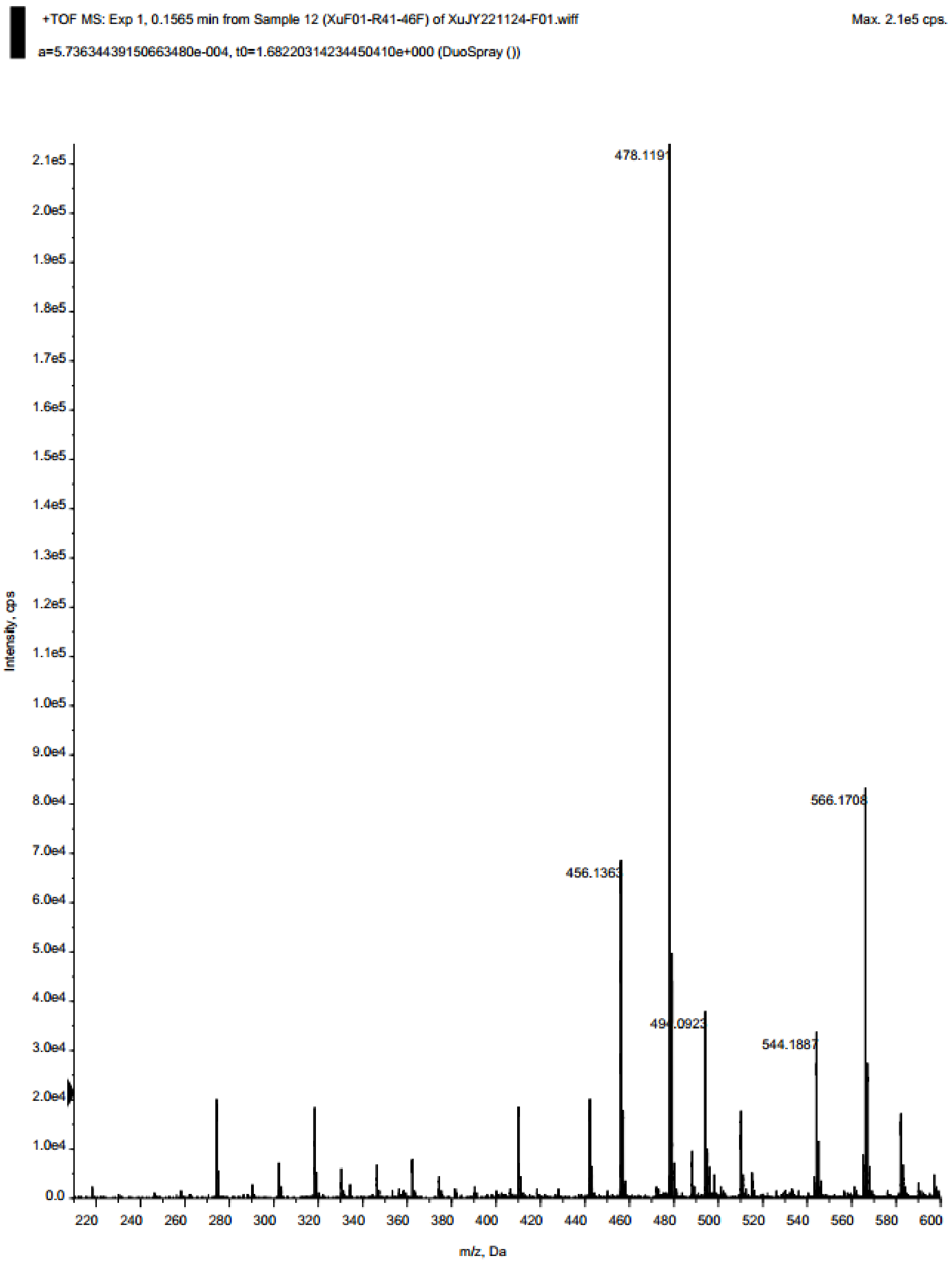

+TOF MS: Exp 1, 0.1565 min from Sample 12 (XuF01-R41-46F) of XuJY221124-F01.wiff
a=5.73634439150663480e-004, t0=1.68220314234450410e+000 (DuoSpray ())
Max. 2.1e5 cps.
478.1191
566.1708
456.1363
544.1887
Intensity, cps
m/z, Da

Compound **4q**

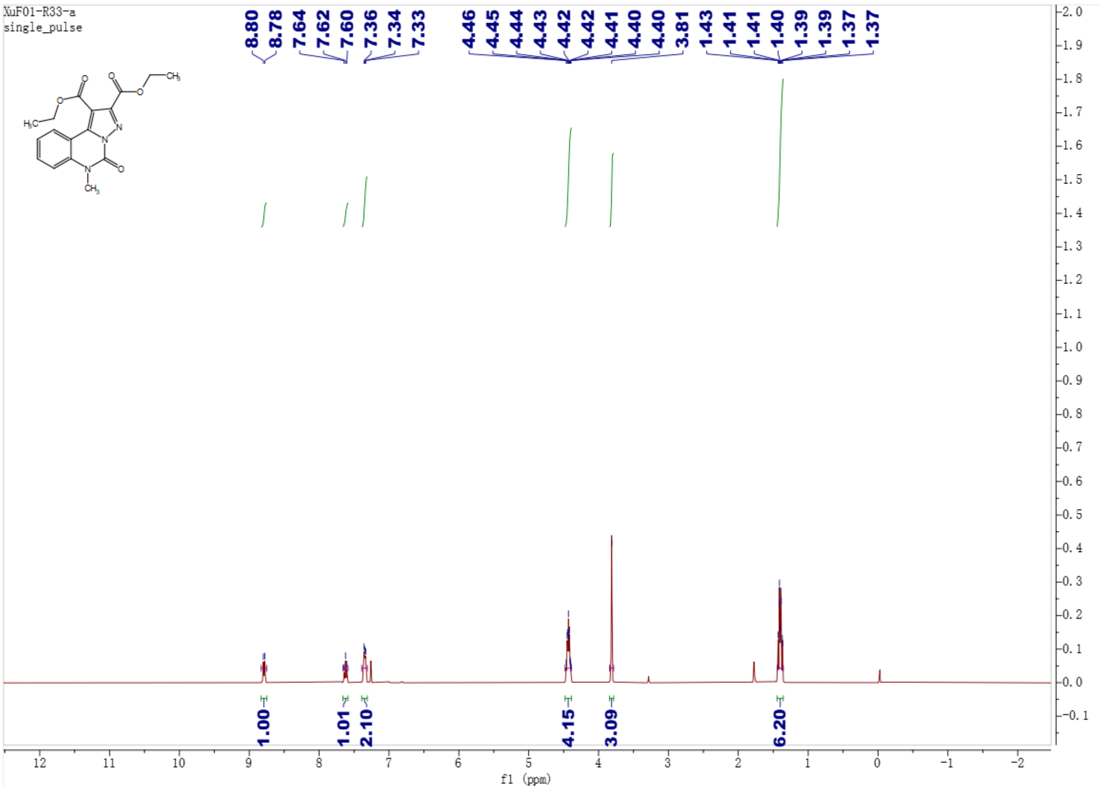
XuF01-R33-a
single_pulse
8.80
8.78
7.64
7.62
7.60
7.36
7.34
7.33
4.46
4.45
4.44
4.43
4.42
4.42
4.41
4.40
4.40
3.81
1.43
1.41
1.41
1.40
1.39
1.39
1.37
1.37
1.00
1.01
2.10
4.15
3.09
6.20
f1 (ppm)

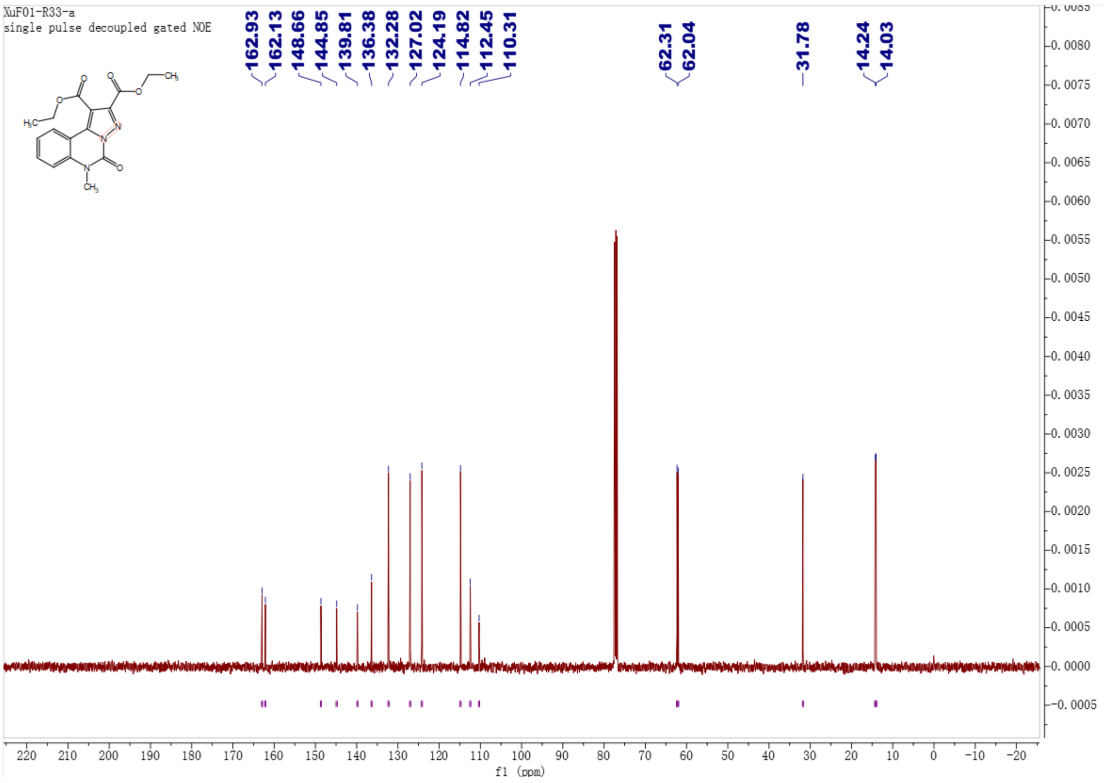
XuF01-R33-a
single pulse decoupled gated NOE
162.93
162.13
148.66
144.85
139.81
136.38
132.28
127.02
124.19
114.82
112.45
110.31
62.31
62.04
31.78
14.24
14.03
f1 (ppm)

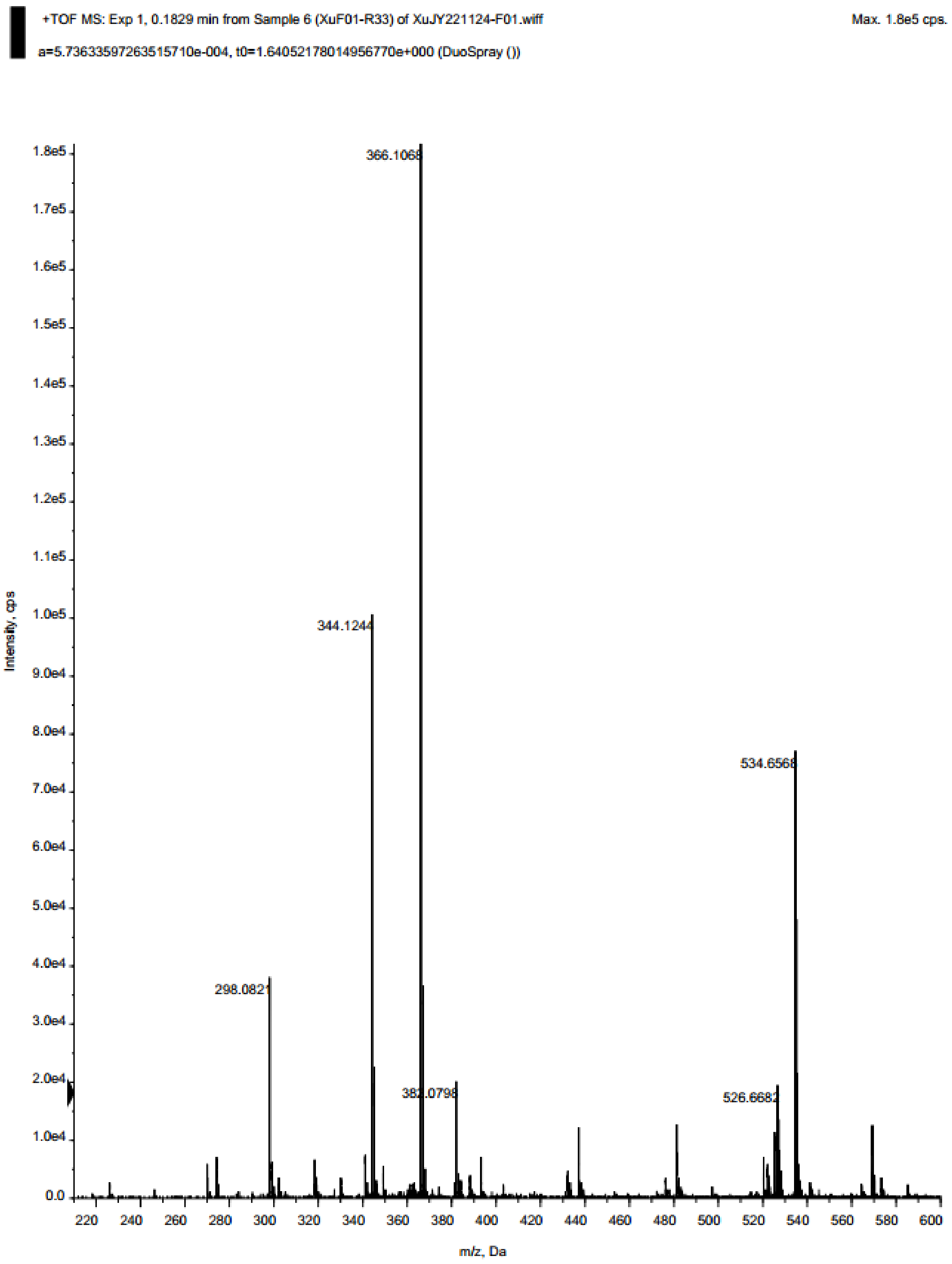

+TOF MS: Exp 1, 0.1829 min from Sample 6 (XuF01-R33) of XuJY221124-F01.wiff
a=5.73633597263515710e-004, t0=1.64052178014956770e+000 (DuoSpray ())
Max. 1.8e5 cps.
366.1068
344.1244
534.6568
298.0821
382.0798
526.6682
Intensity, cps
m/z, Da

Compound **4r**

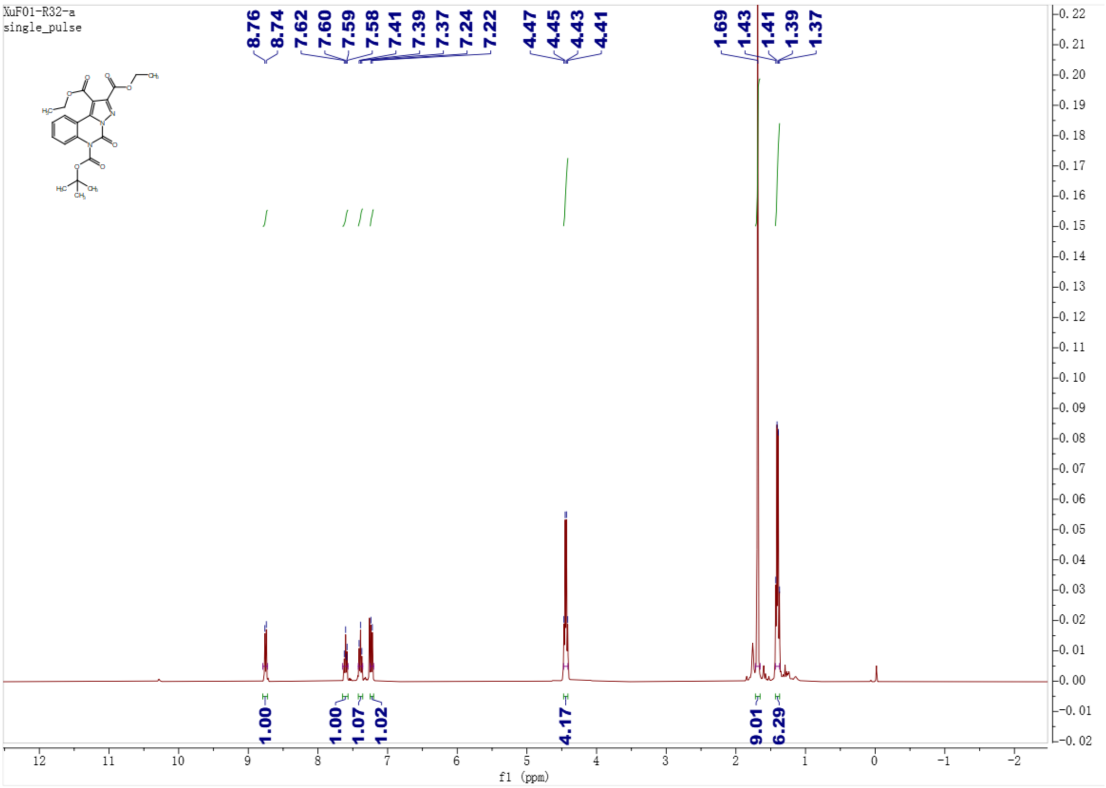

XuF01-R32-a
single_pulse
8.76
8.74
7.62
7.60
7.59
7.58
7.41
7.39
7.37
7.24
7.22
4.47
4.45
4.43
4.41
1.69
1.43
1.41
1.39
1.37
1.00
1.00
1.07
1.02
4.17
9.01
6.29
f1 (ppm)

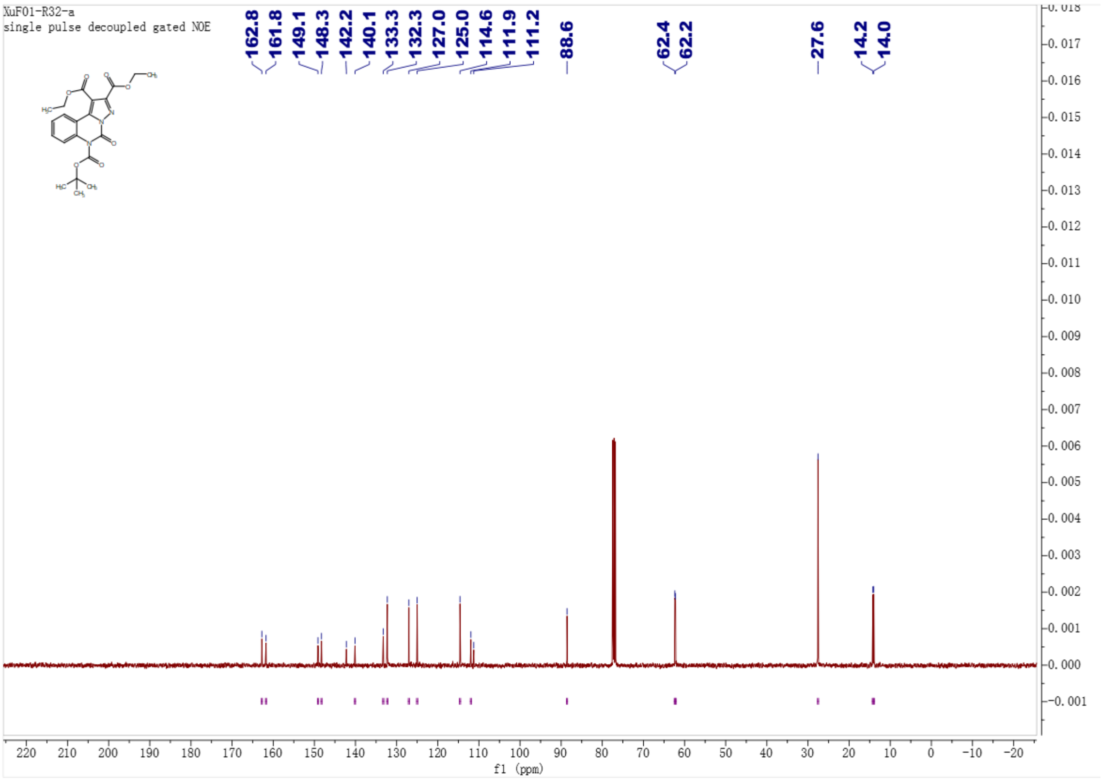

XuF01-R32-a
single pulse decoupled gated NOE
162.8
161.8
149.1
148.3
142.2
140.1
133.3
132.3
127.0
125.0
114.6
111.9
111.2
88.6
62.4
62.2
27.6
14.2
14.0
f1 (ppm)

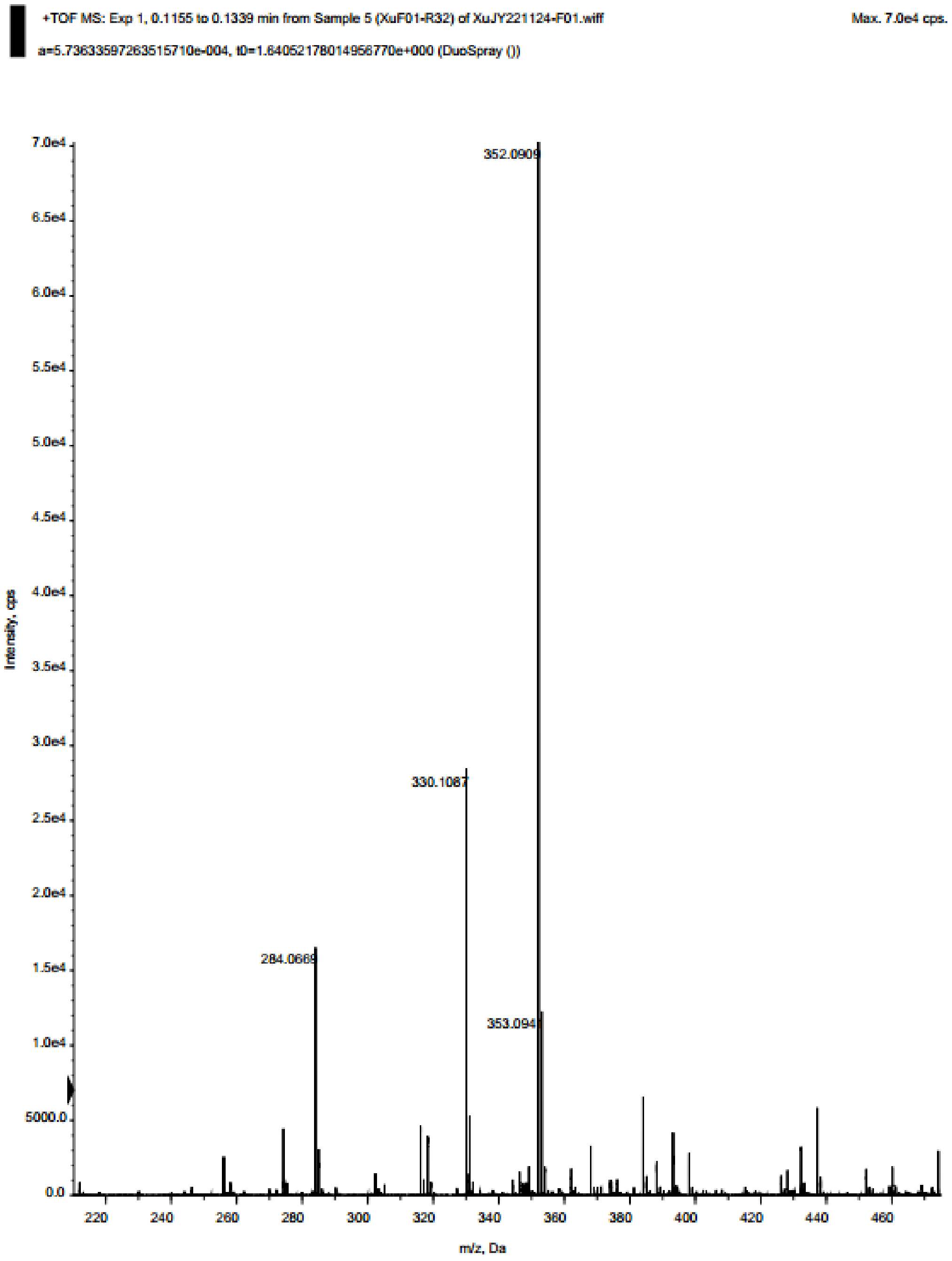
+TOF MS: Exp 1, 0.1155 to 0.1339 min from Sample 5 (XuF01-R32) of XuJY221124-F01.wiff
Max. 7.0e4 cps.
a=5.73633597263515710e-004, t0=1.64052178014956770e+000 (DuoSpray ())
352.0909
330.1087
284.0669
353.094
Intensity, cps
m/z, Da

Compound **4s**

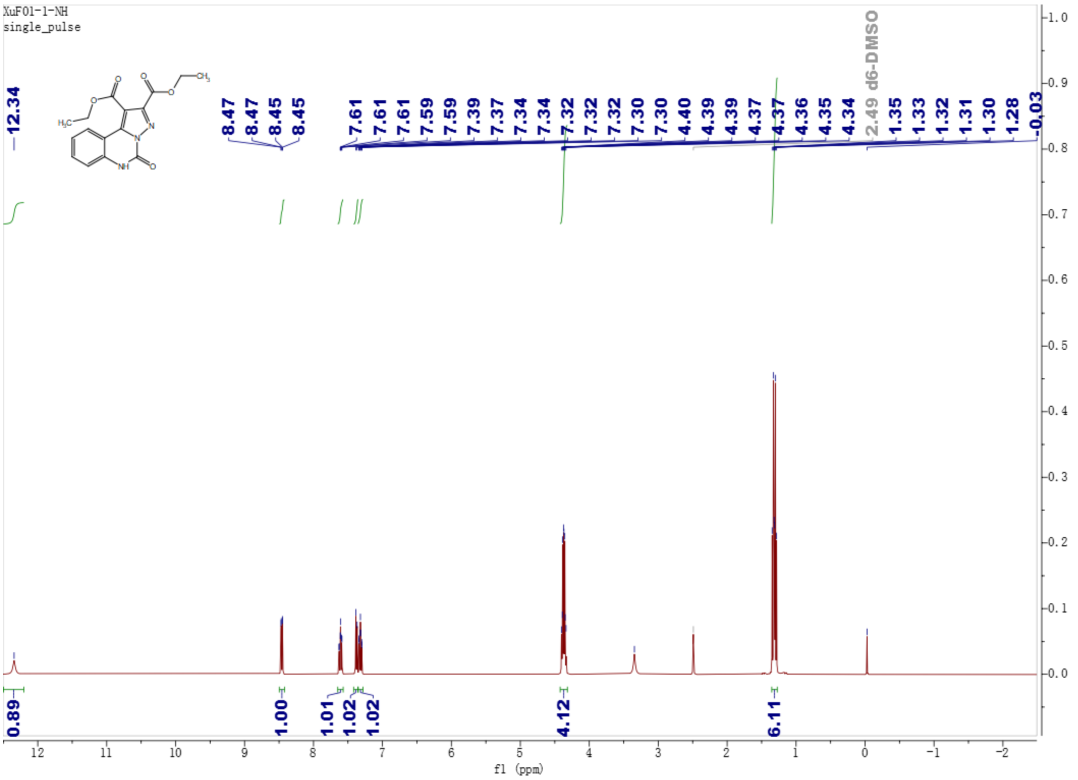
XuF01-1-NH
single_pulse
12.34
8.47 8.47 8.45 8.45
7.61 7.61 7.61 7.59 7.59 7.39 7.37 7.34 7.34 7.32 7.32 7.32 7.30 7.30
4.40 4.39 4.39 4.37 4.37 4.36 4.35 4.34
2.49 d6-DMSO
1.35 1.33 1.32 1.31 1.30 1.28
-0.03
0.89
1.00
1.01
1.02
1.02
4.12
6.11
f1 (ppm)

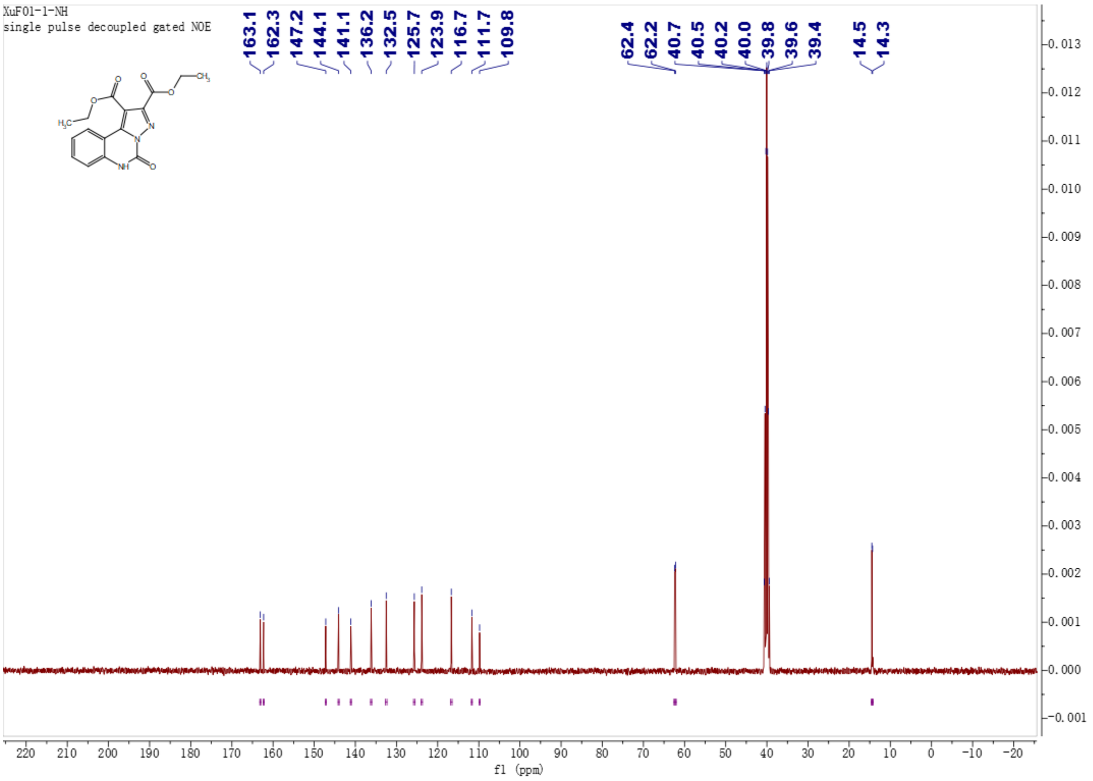
XuF01-1-NH
single pulse decoupled gated NOE
163.1 162.3 147.2 144.1 141.1 136.2 132.5 125.7 123.9 116.7 111.7 109.8
62.4 62.2 40.7 40.5 40.2 40.0 39.8 39.6 39.4
14.5 14.3
f1 (ppm)

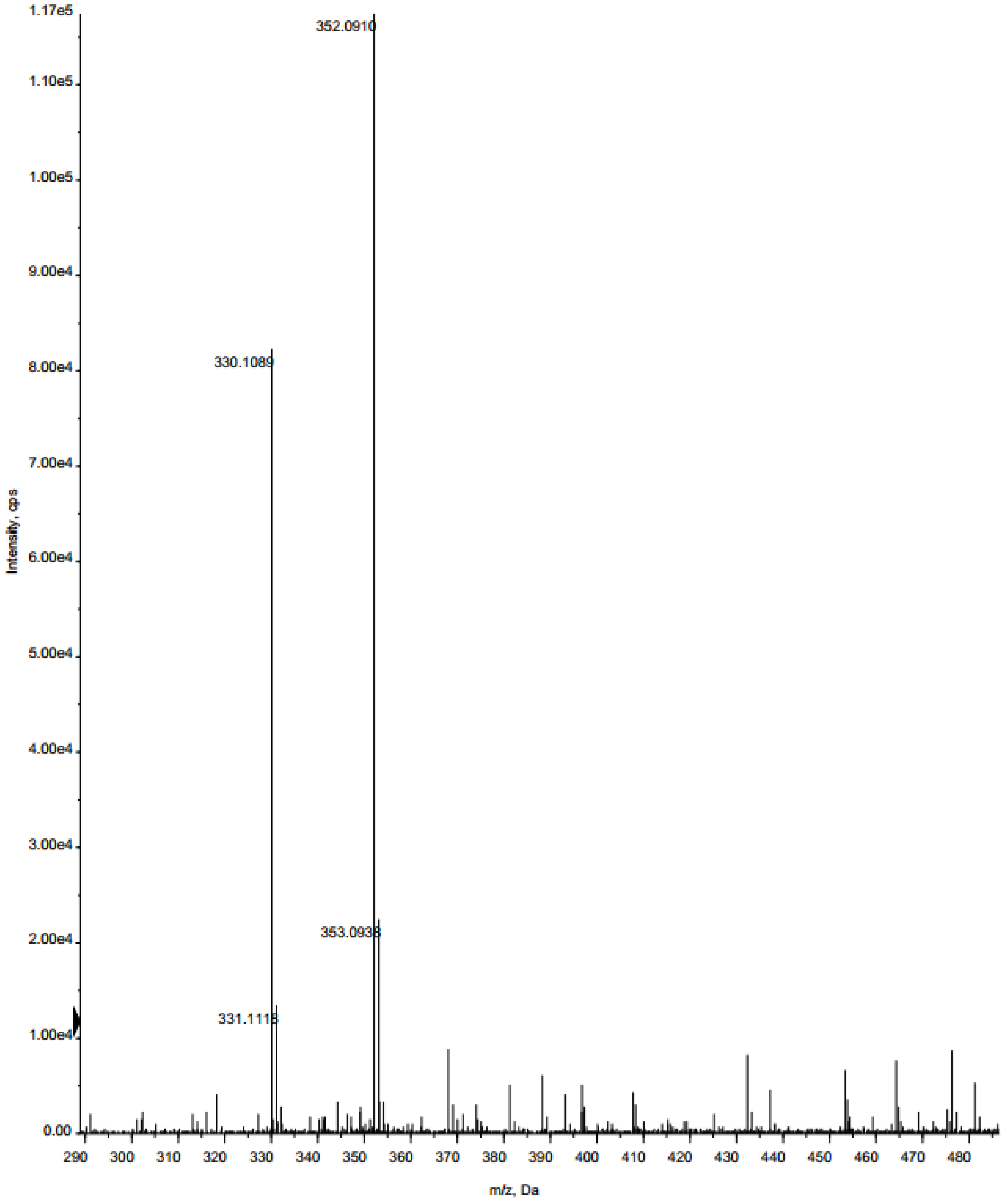
352.0910
330.1089
353.0938
331.1118
Intensity, cps
m/z, Da

Compound **4t**

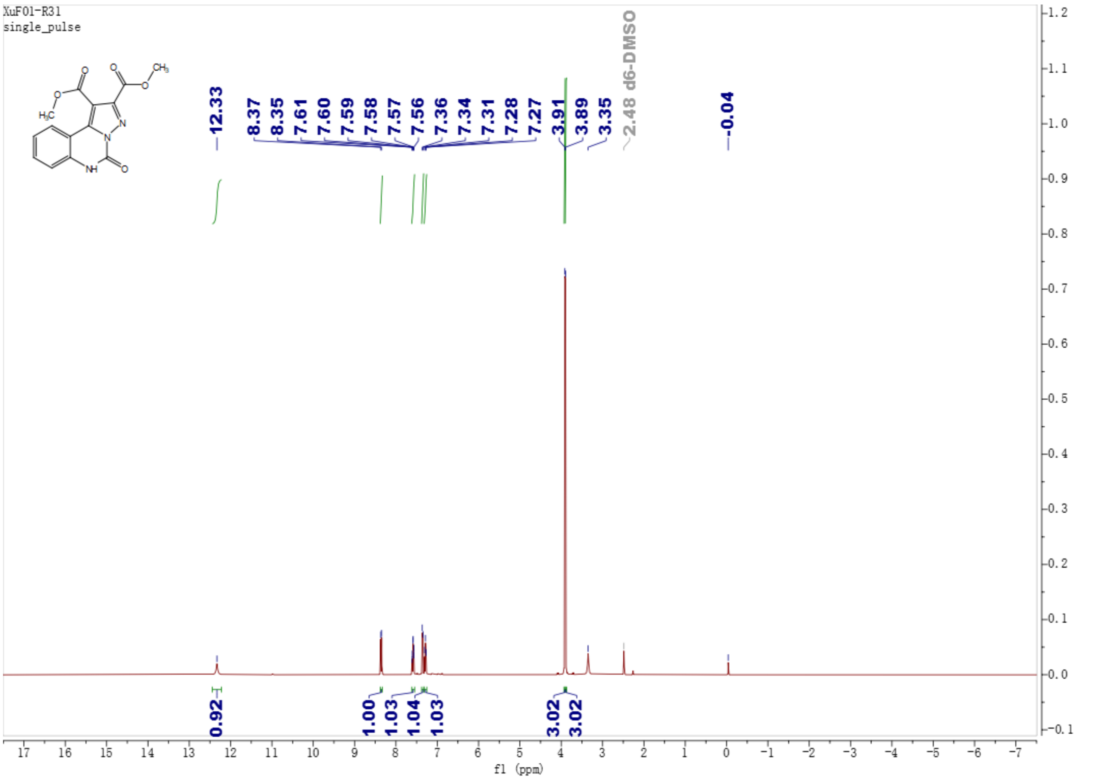
XuF01-R31
single_pulse
12.33
8.37
8.35
7.61
7.60
7.59
7.58
7.57
7.56
7.36
7.34
7.31
7.28
7.27
3.91
3.89
3.35
2.48 d6-DMSO
-0.04
0.92
1.00
1.03
1.04
1.03
3.02
3.02
f1 (ppm)

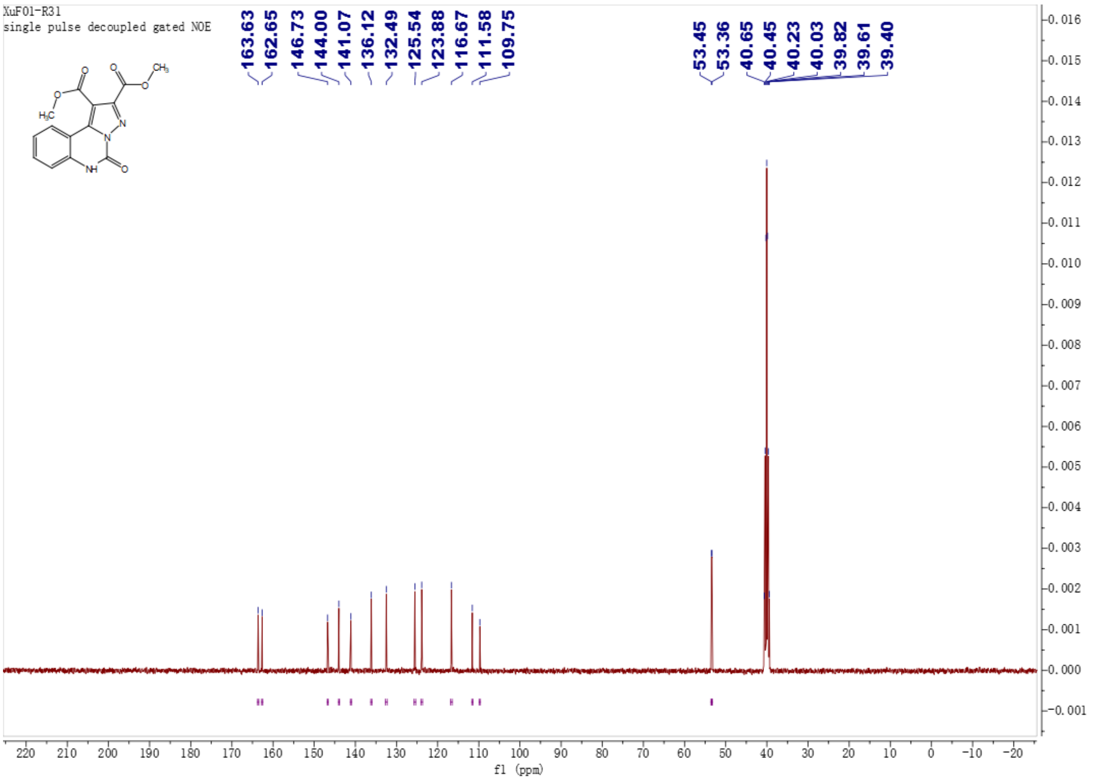
XuF01-R31
single pulse decoupled gated NOE
163.63
162.65
146.73
144.00
141.07
136.12
132.49
125.54
123.88
116.67
111.58
109.75
53.45
53.36
40.65
40.45
40.23
40.03
39.82
39.61
39.40
f1 (ppm)

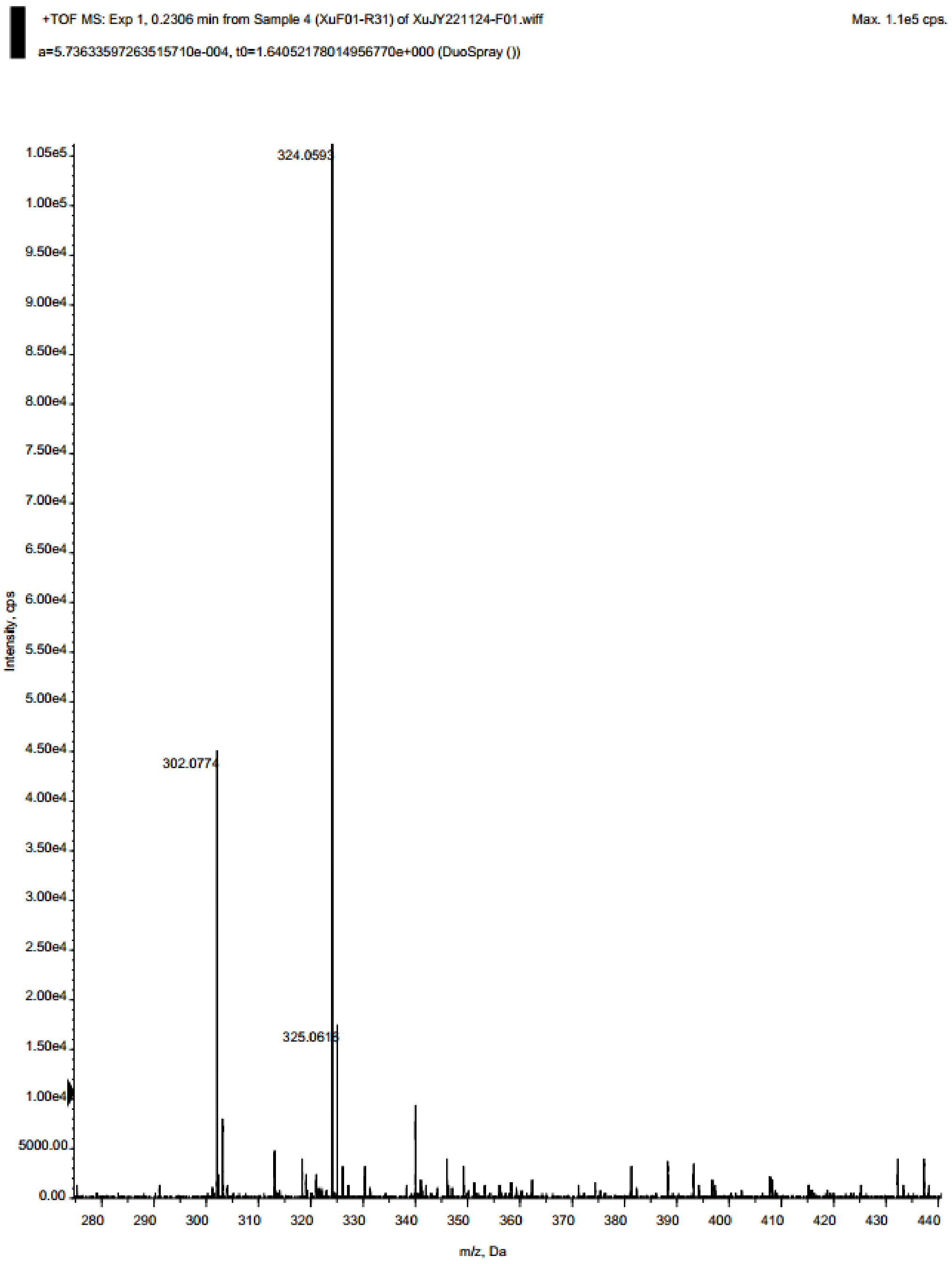
+TOF MS: Exp 1, 0.2306 min from Sample 4 (XuF01-R31) of XuJY221124-F01.wiff
Max. 1.1e5 cps.
a=5.73633597263515710e-004, t0=1.64052178014956770e+000 (DuoSpray ())
324.0593
302.0774
325.0618
Intensity, cps
m/z, Da

Compound **4u**

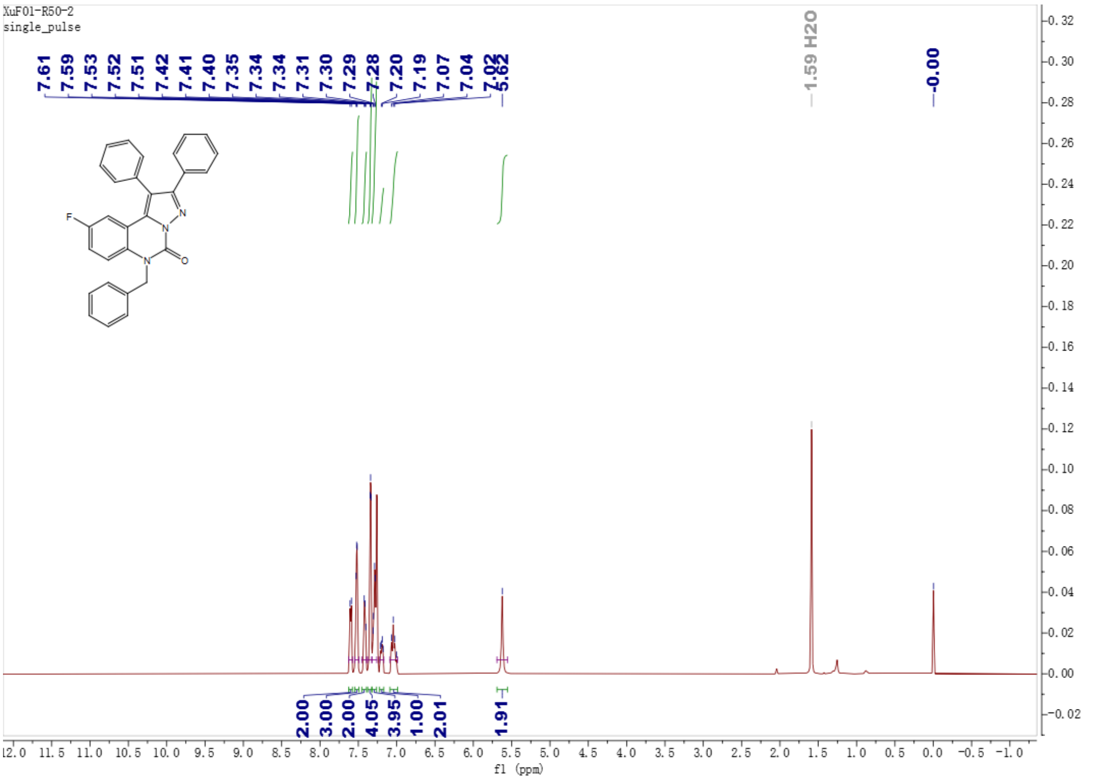

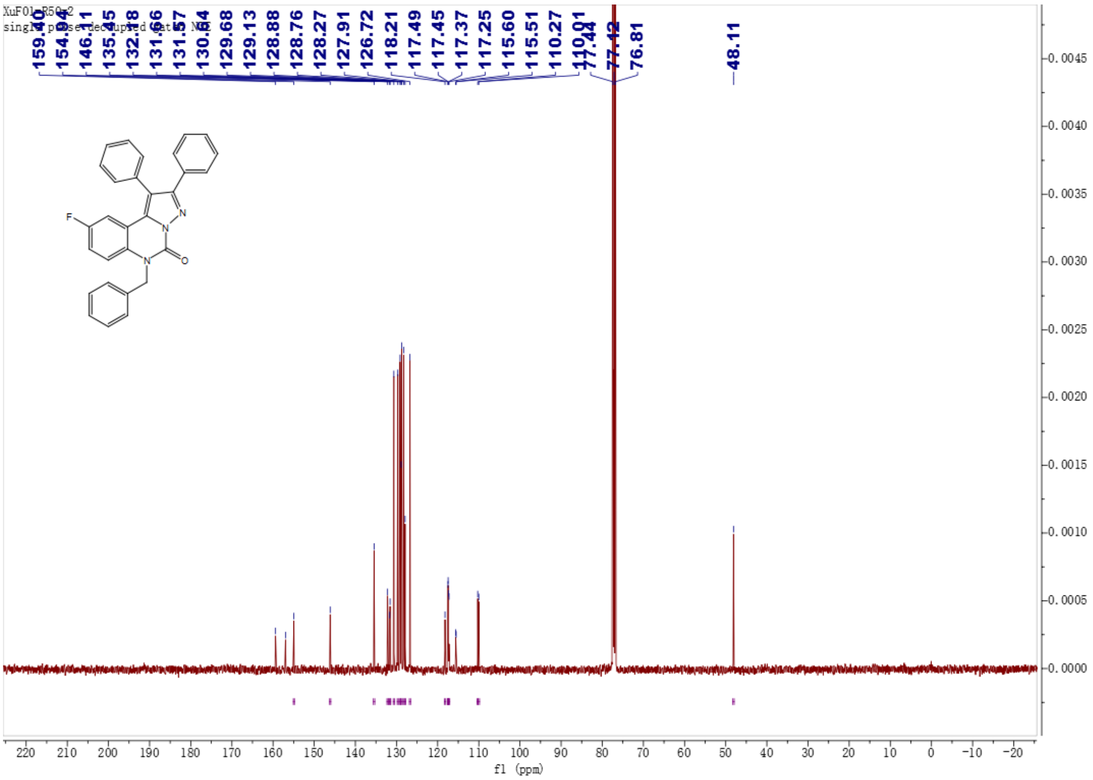

+TOF MS: Exp 1, 0.1746 min from Sample 17 (XuF01-R50) of XuJY221124-F01.wiff   Max. 1.3e5 cps.

a=5.73634439150663480e-004, t0=1.68220314234450410e+000 (DuoSpray ())

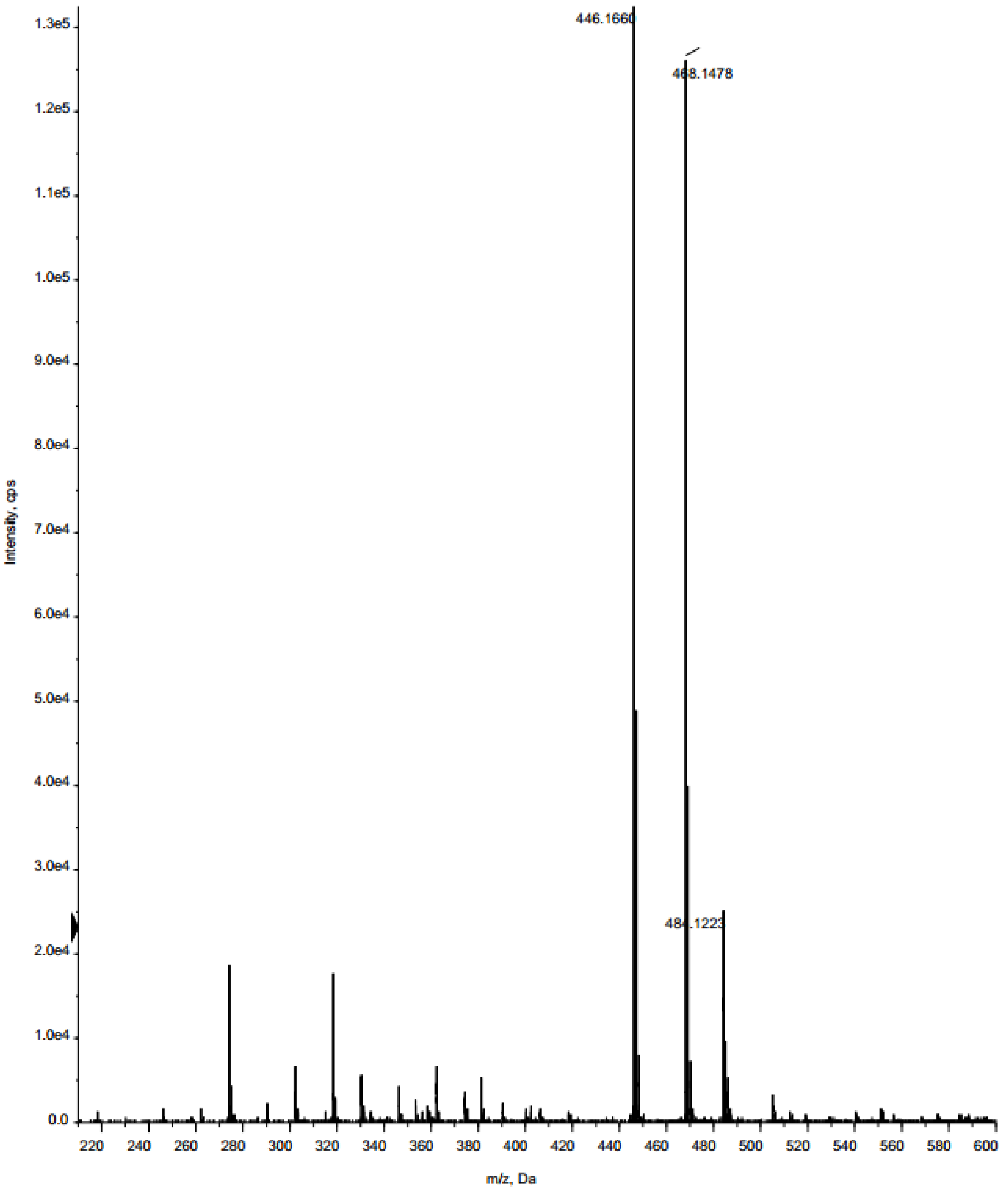

Compound **4v**

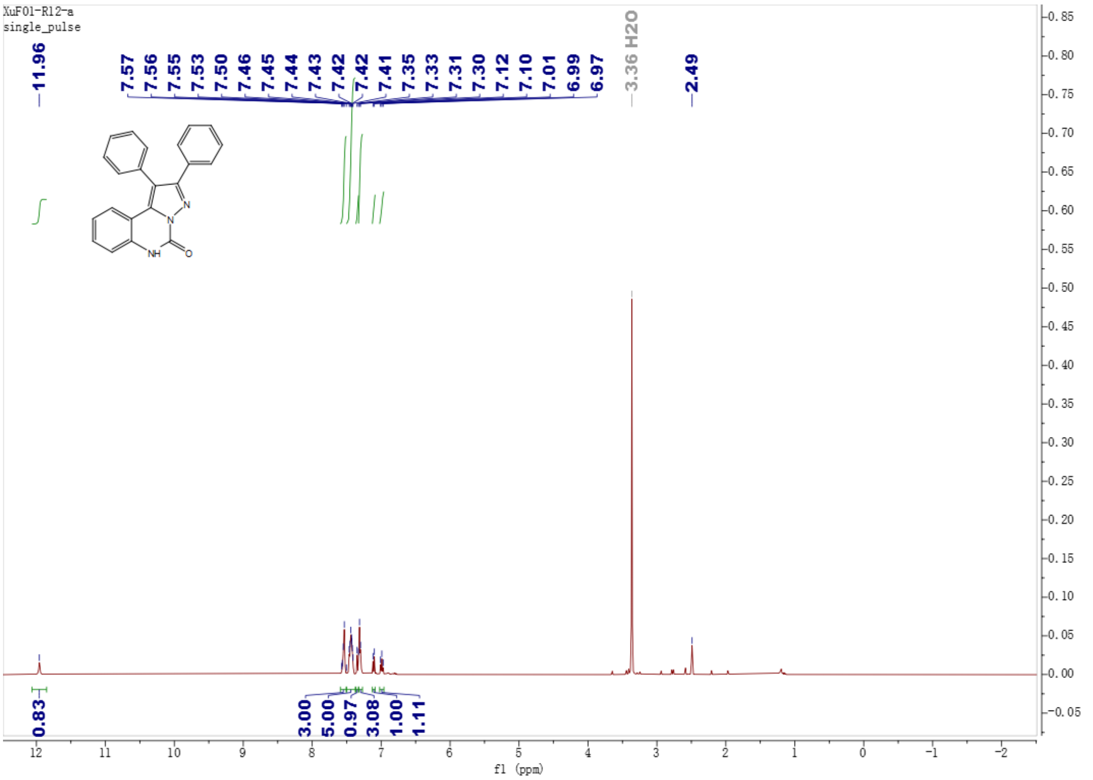

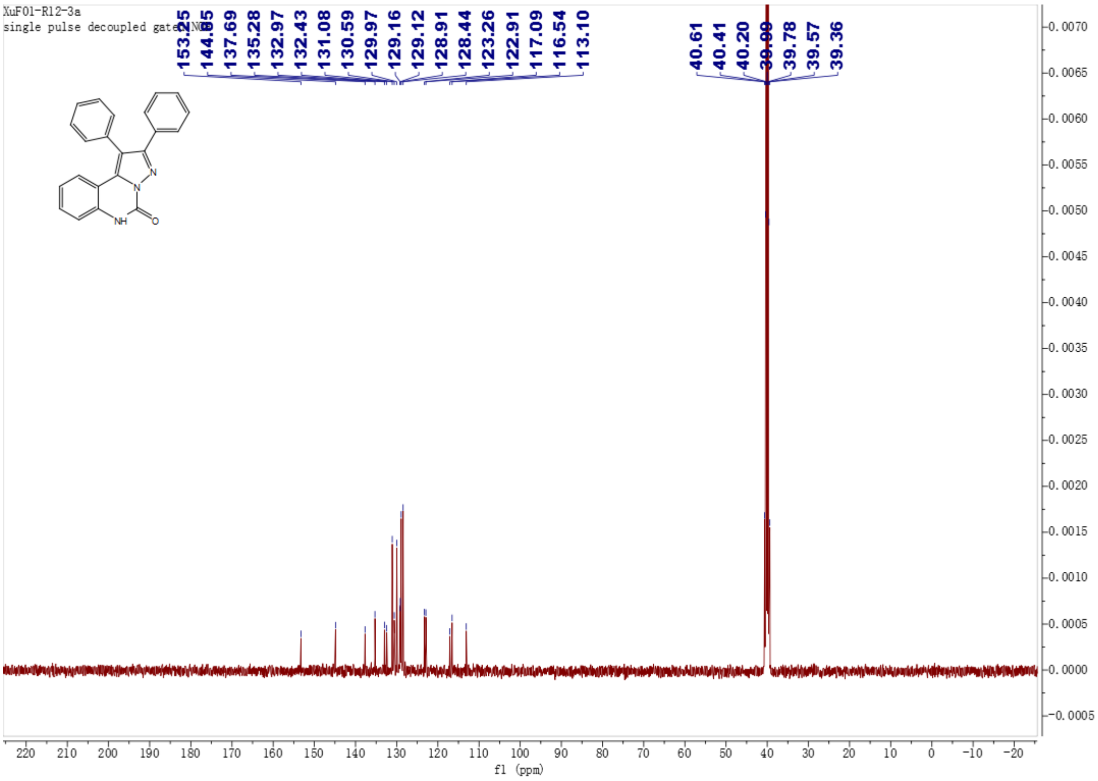

+TOF MS: Exp 1, 0.1569 min from Sample 25 (XuF01-R12-3a) of XuJY221124-F01.wiff

Max. 1.3e5 cps.

a=5.73633154282262030e-004, t0=1.67858104780612340e+000 (DuoSpray ())

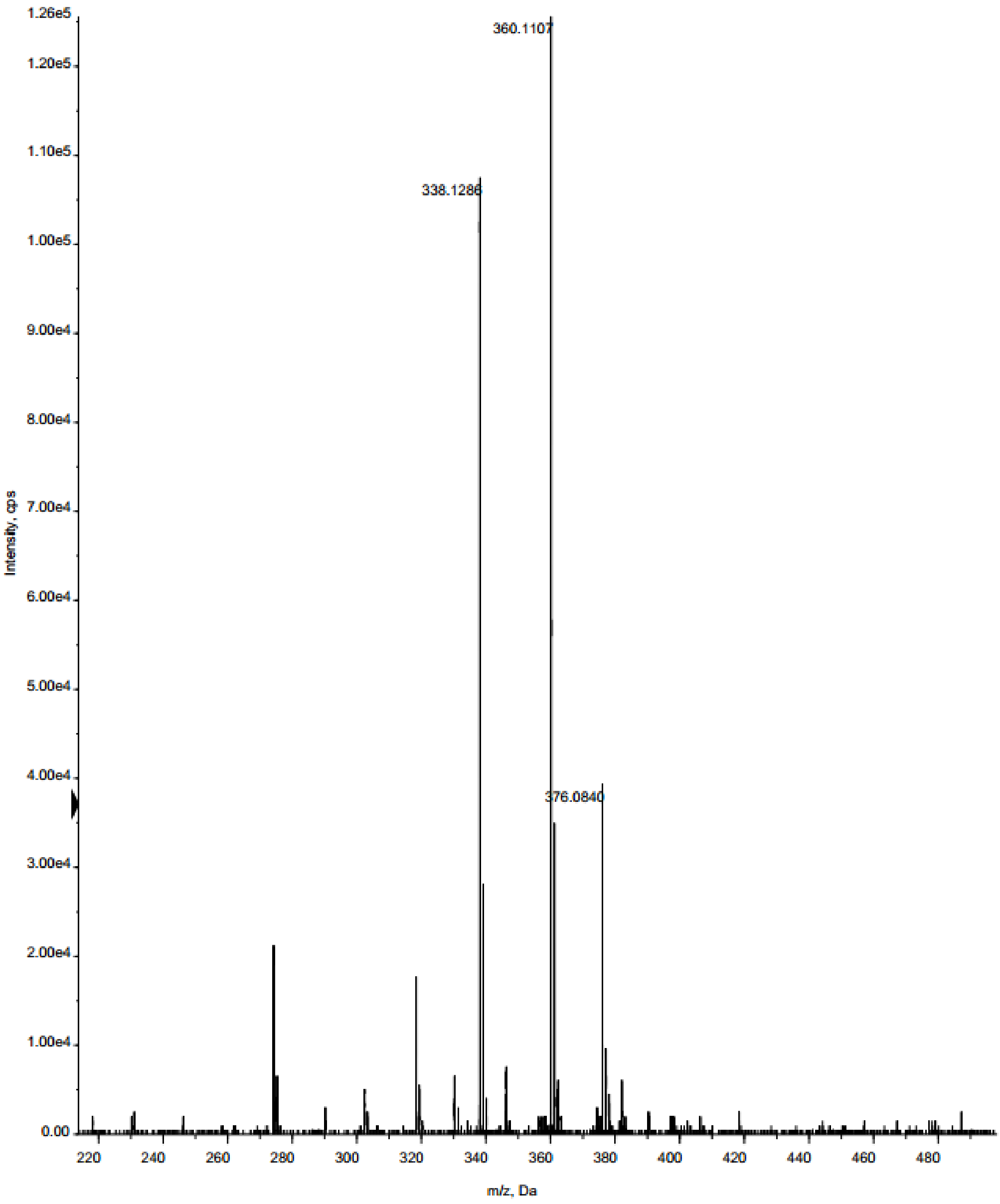

6. Figure S4. Copies of HPLC analysis for products **4a** – **4v**

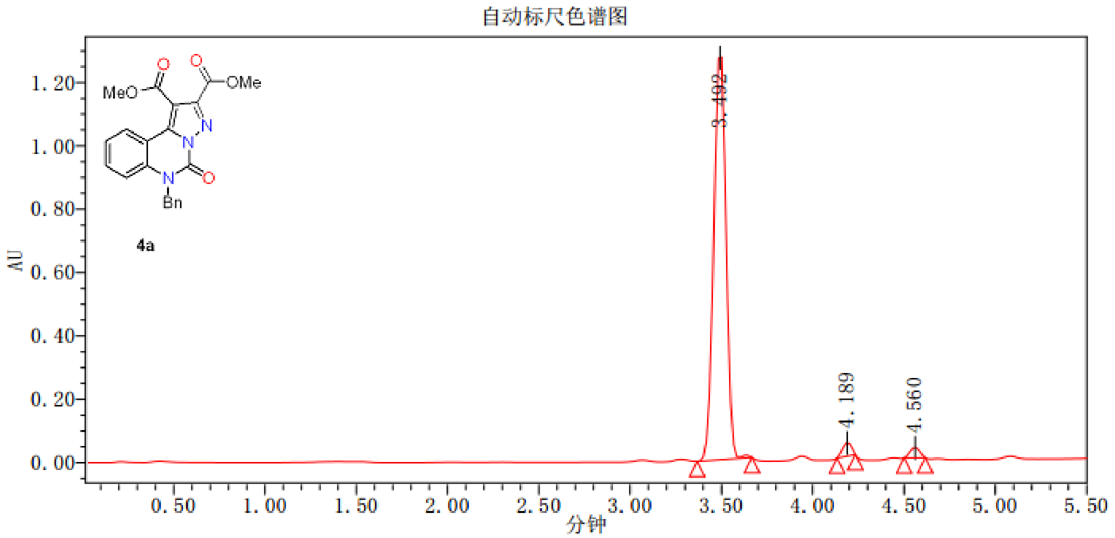

峰结果

	名称	保留时间 (分钟)	面积 (微伏*秒)	高度 (微伏)	% 面积
1		3. 492	5953935	1276421	95. 74
2		4. 189	143115	41273	2. 30
3		4. 560	122075	31542	1. 96

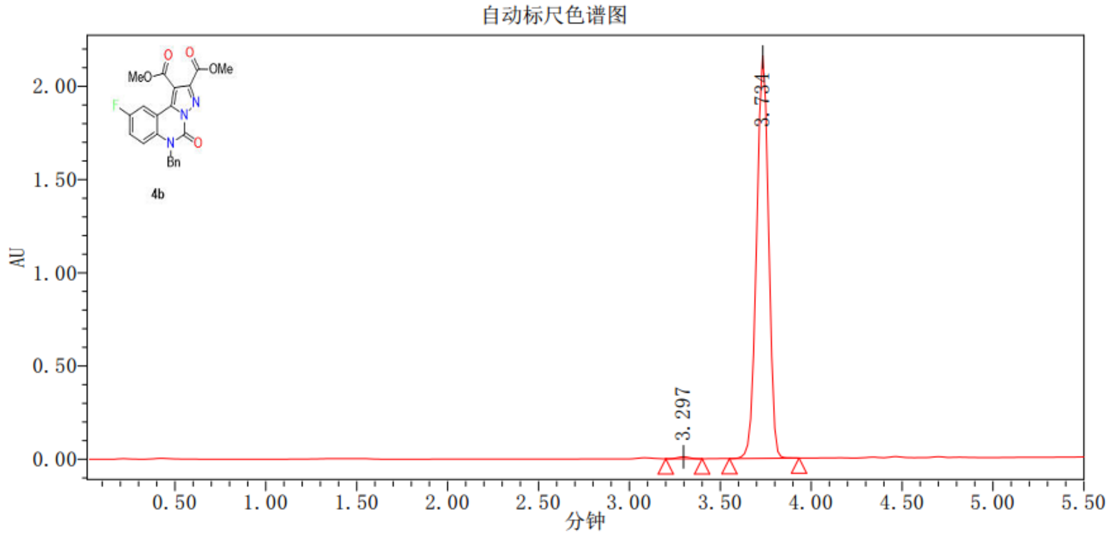

峰结果

	名称	保留时间 (分钟)	面积 (微伏*秒)	高度 (微伏)	% 面积
1		3. 297	47318	10100	0. 47
2		3. 734	9917407	2126829	99. 53

自动标尺色谱图

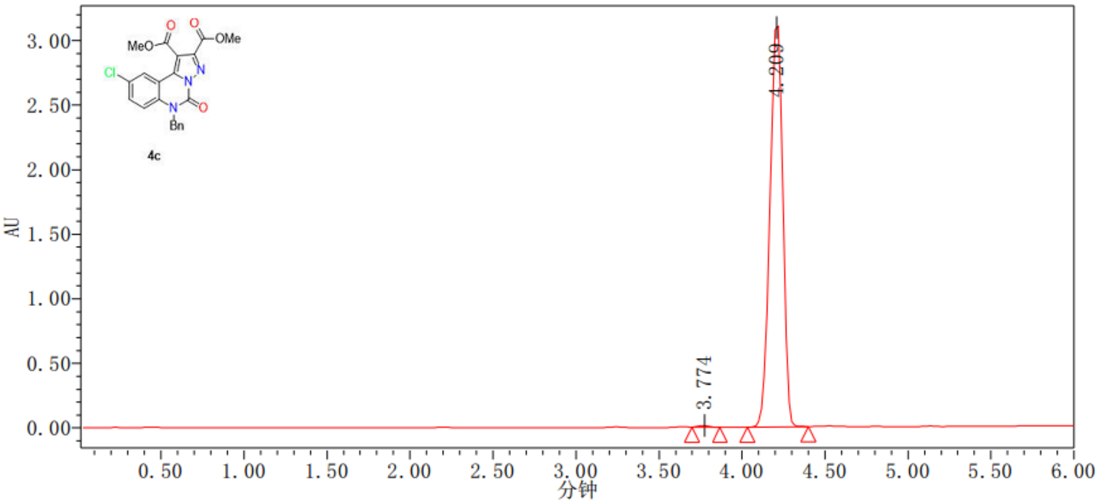

峰结果

	名称	保留时间(分钟)	面积(微伏*秒)	高度(微伏)	% 面积
1		3.774	63654	13804	0.38
2		4.209	16825486	3142763	99.62

自动标尺色谱图

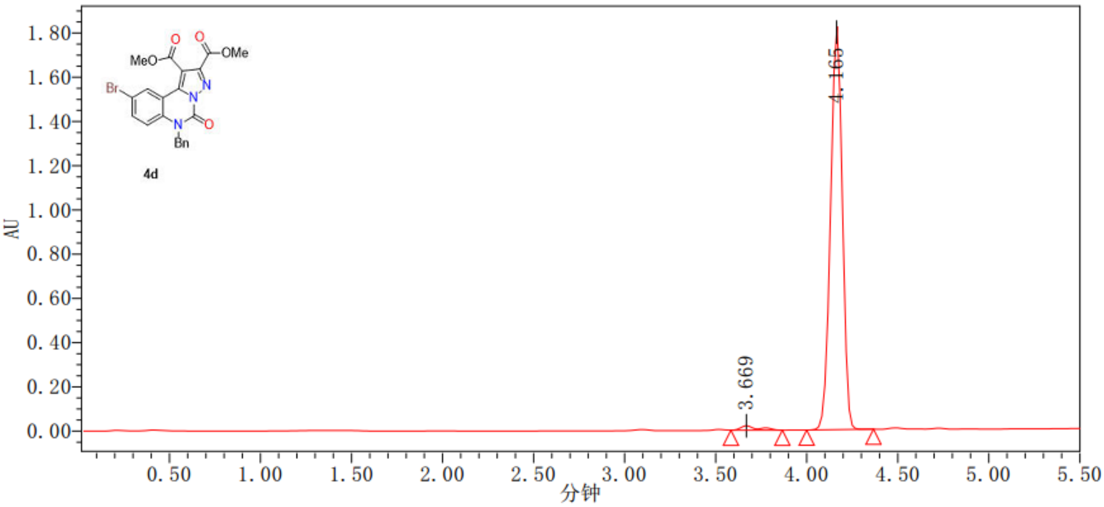

峰结果

	名称	保留时间(分钟)	面积(微伏*秒)	高度(微伏)	% 面积
1		3.669	135297	20094	1.63
2		4.165	8187183	1788783	98.37

自动标尺色谱图

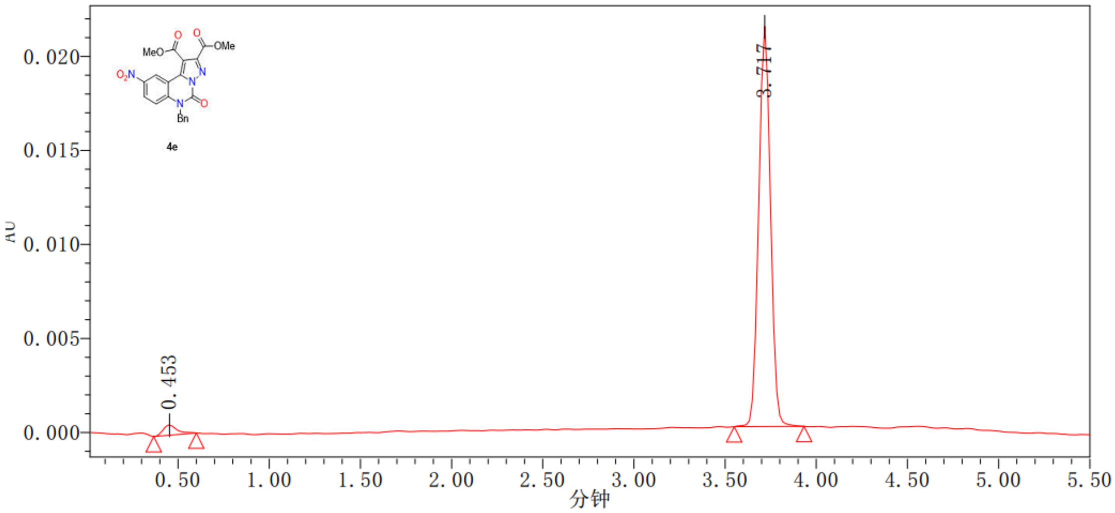

峰结果

	名称	保留时间（分钟）	面积（微伏*秒）	高度（微伏）	% 面积
1		0.453	2927	538	2.97
2		3.717	95629	20879	97.03

自动标尺色谱图

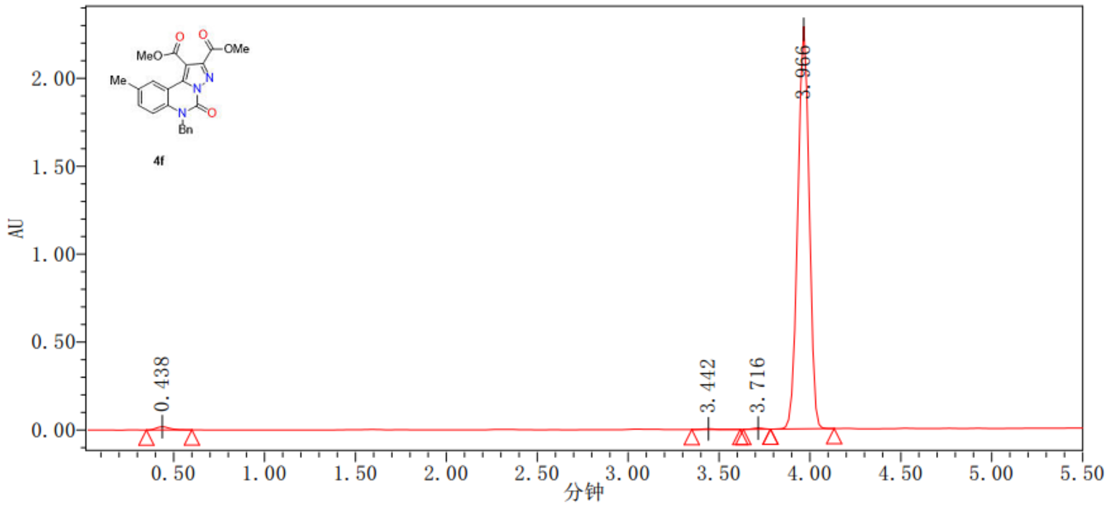

峰结果

	名称	保留时间（分钟）	面积（微伏*秒）	高度（微伏）	% 面积
1		0.438	96361	18734	0.95
2		3.442	20397	4259	0.20
3		3.716	28045	6854	0.28
4		3.966	9957005	2232826	98.57

自动标尺色谱图

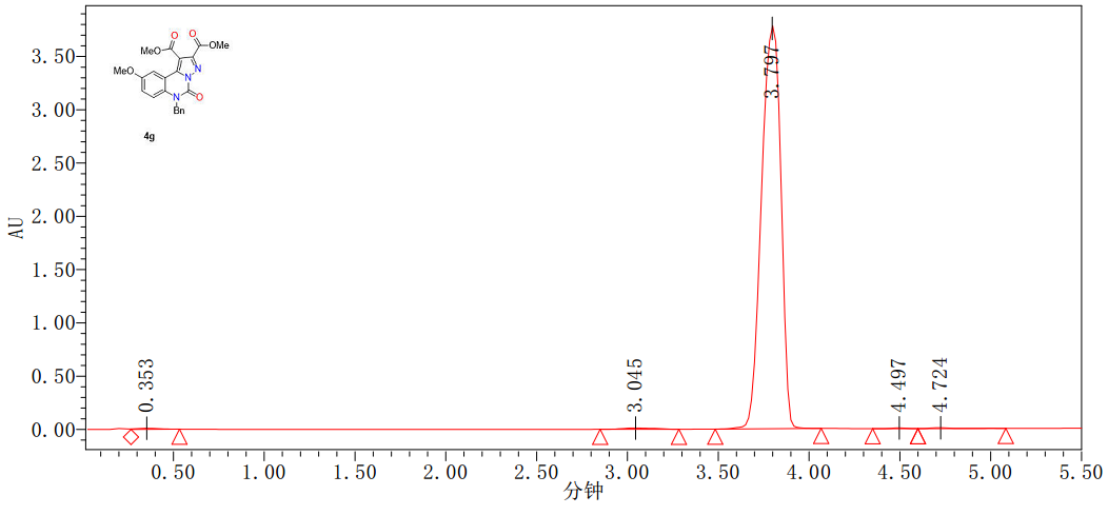

峰结果

	名称	保留时间（分钟）	面积（微伏*秒）	高度（微伏）	% 面积
1		0.353	82678	9769	0.29
2		3.045	147306	12247	0.52
3		3.797	28045879	3790771	98.82
4		4.497	35127	6908	0.12
5		4.724	70385	7598	0.25

自动标尺色谱图

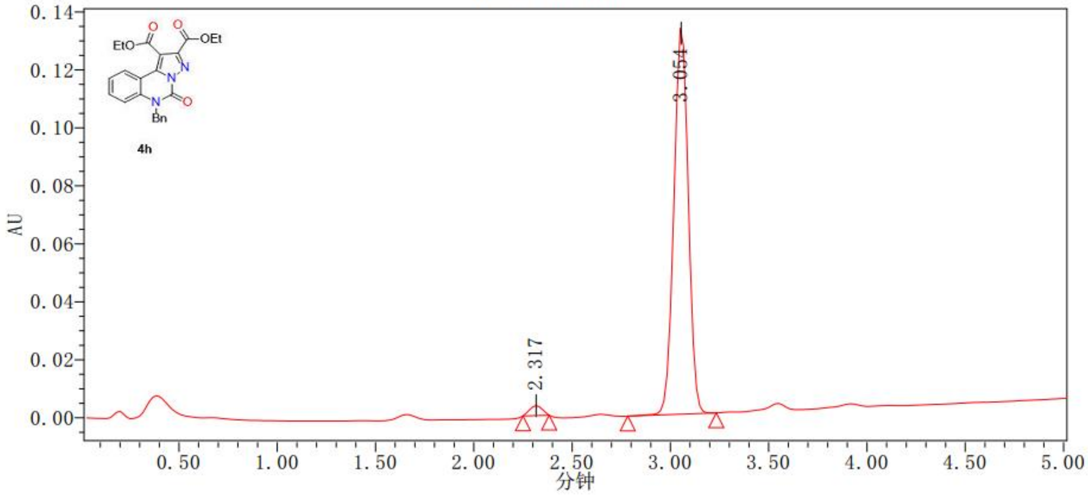

峰结果

	名称	保留时间（分钟）	面积（微伏*秒）	高度（微伏）	% 面积
1		2.317	15364	3376	2.20
2		3.054	682139	132374	97.80

自动标尺色谱图

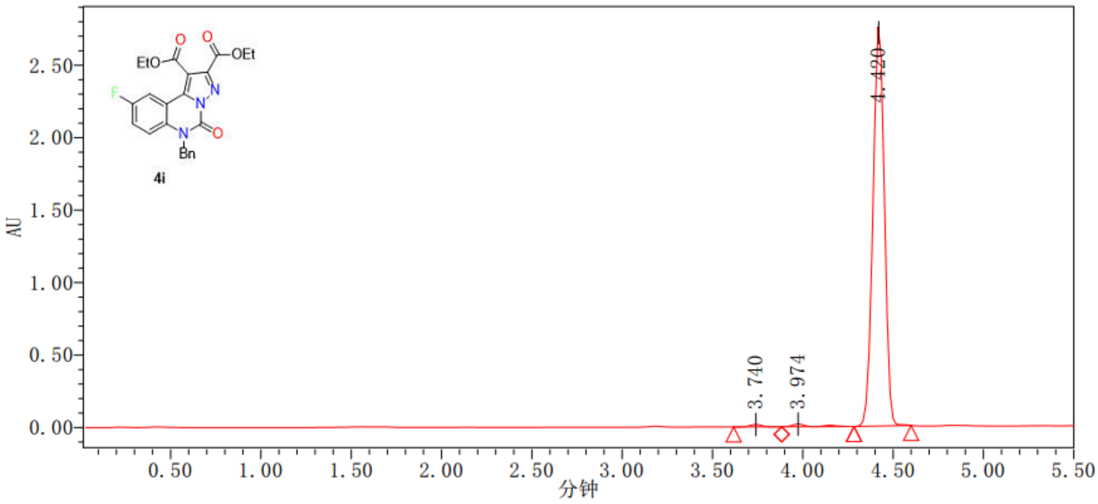

峰结果

	名称	保留时间（分钟）	面积（微伏*秒）	高度（微伏）	% 面积
1		3.740	76012	16524	0.62
2		3.974	124815	17919	1.01
3		4.420	12122559	2715912	98.37

自动标尺色谱图

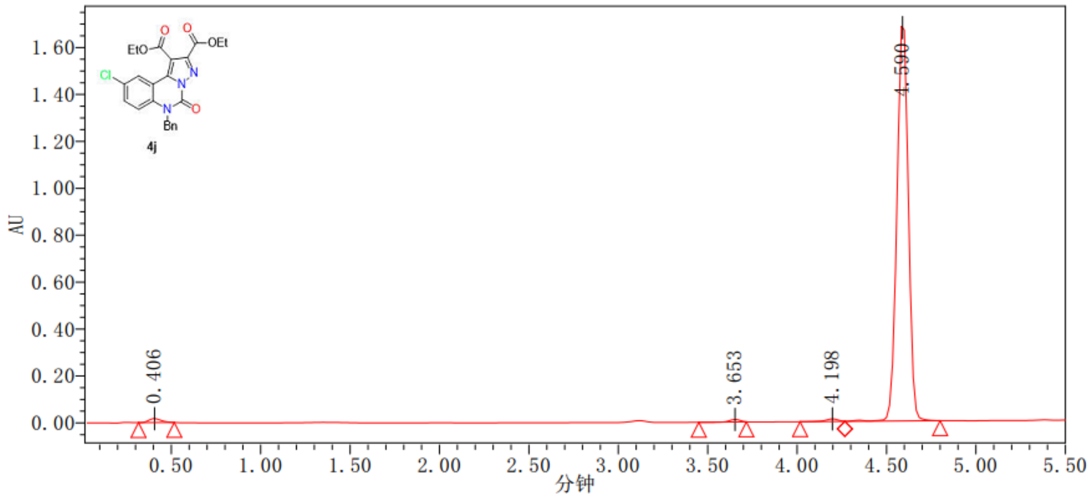

峰结果

	名称	保留时间（分钟）	面积（微伏*秒）	高度（微伏）	% 面积
1		0.406	83227	16943	1.07
2		3.653	45856	10848	0.59
3		4.198	55342	11256	0.71
4		4.590	7599570	1685138	97.63

自动标尺色谱图

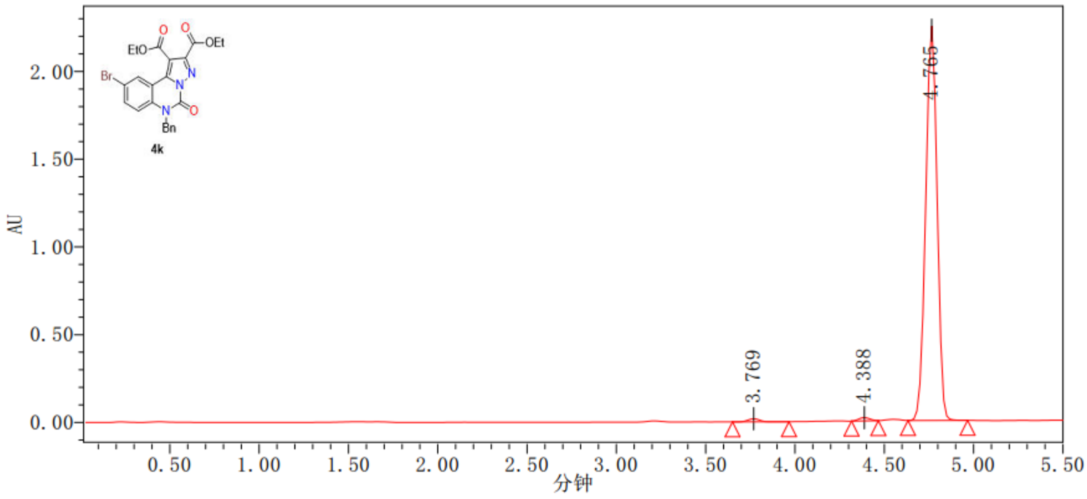

峰结果

	名称	保留时间（分钟）	面积（微伏*秒）	高度（微伏）	% 面积
1		3.769	78762	17503	0.78
2		4.388	79339	18610	0.78
3		4.765	9958936	2208542	98.44

自动标尺色谱图

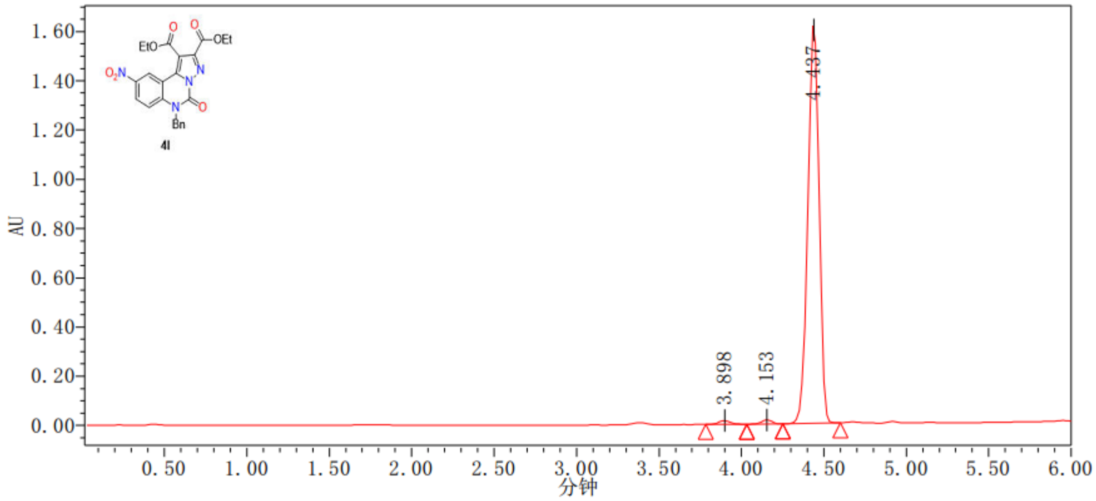

峰结果

	名称	保留时间（分钟）	面积（微伏*秒）	高度（微伏）	% 面积
1		3.898	74867	14458	0.95
2		4.153	73741	15949	0.94
3		4.437	7692606	1605166	98.10

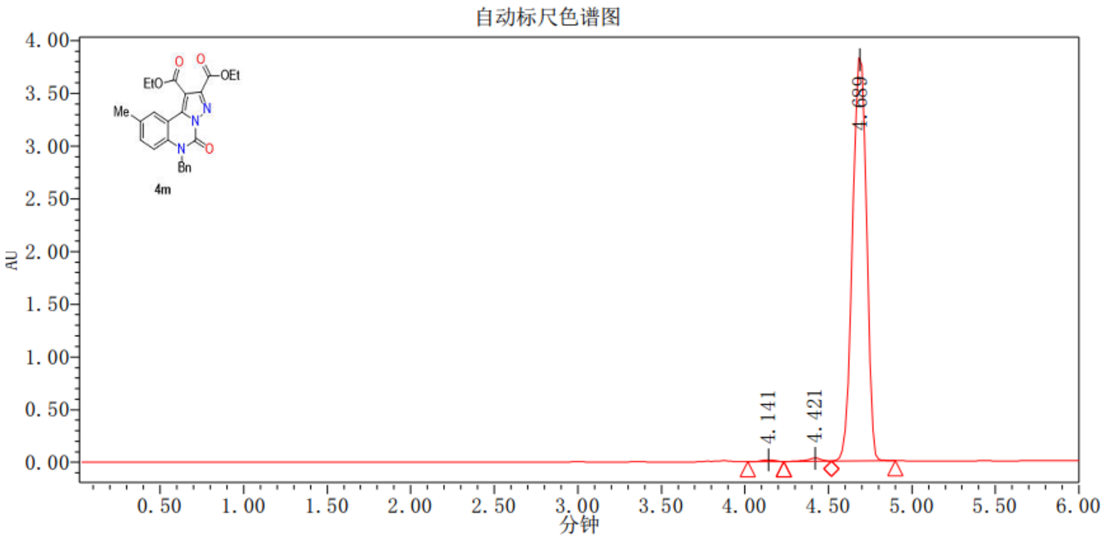

峰结果

	名称	保留时间（分钟）	面积（微伏*秒）	高度（微伏）	% 面积
1		4.141	93429	16554	0.41
2		4.421	174441	30042	0.77
3		4.689	22444223	3835916	98.82

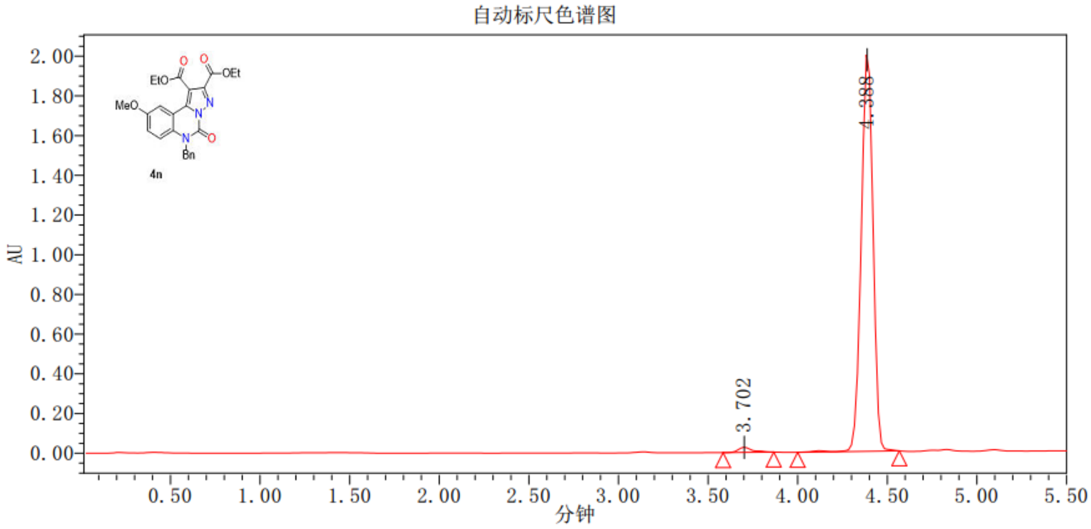

峰结果

	名称	保留时间（分钟）	面积（微伏*秒）	高度（微伏）	% 面积
1		3.702	138009	25178	1.48
2		4.388	9168974	1983171	98.52

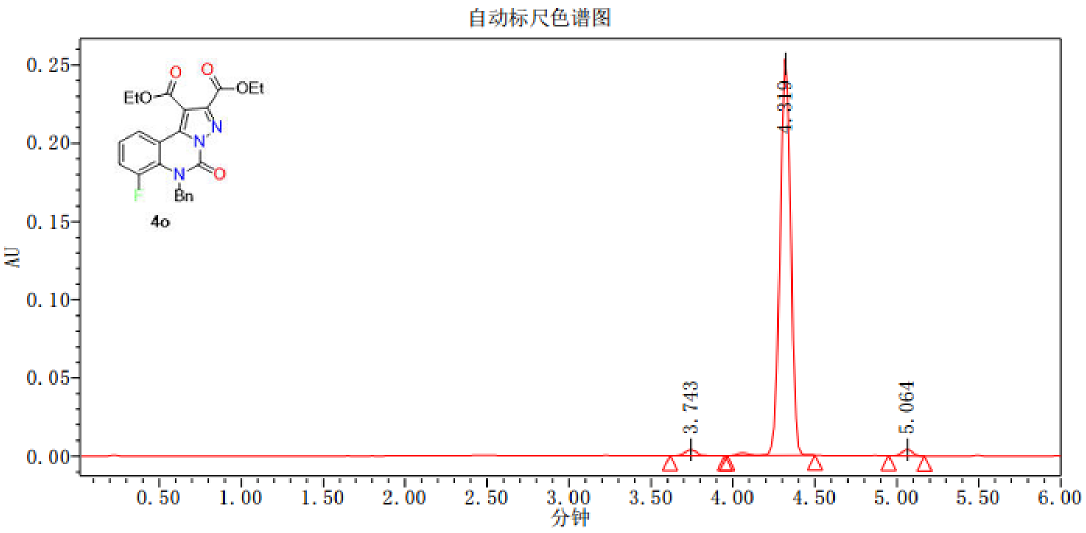

峰结果

	名称	保留时间（分钟）	面积（微伏*秒）	高度（微伏）	% 面积
1		3.743	17866	3581	1.50
2		4.319	1158093	249894	97.03
3		5.064	17576	3964	1.47

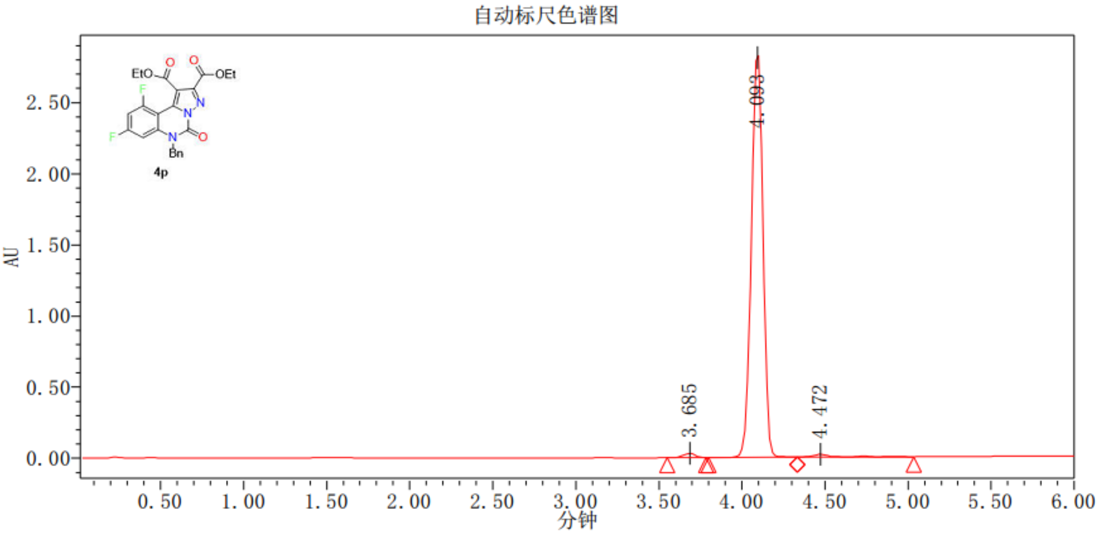

峰结果

	名称	保留时间（分钟）	面积（微伏*秒）	高度（微伏）	% 面积
1		3.685	155059	30397	1.10
2		4.093	13820357	2838191	97.63
3		4.472	179897	20979	1.27

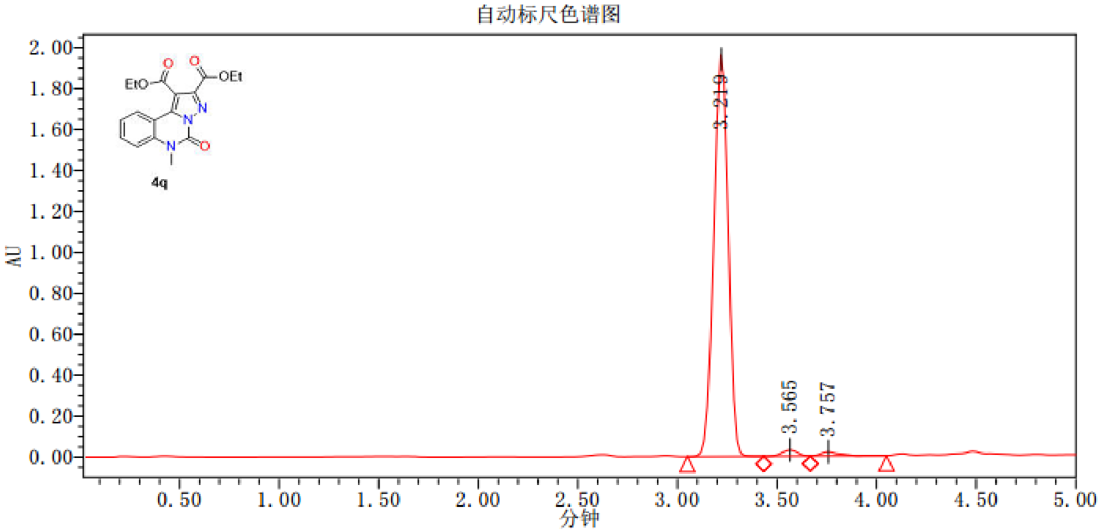

峰结果

	名称	保留时间（分钟）	面积（微伏*秒）	高度（微伏）	% 面积
1		3.219	9872383	1946189	97.03
2		3.565	167080	30674	1.64
3		3.757	135226	20745	1.33

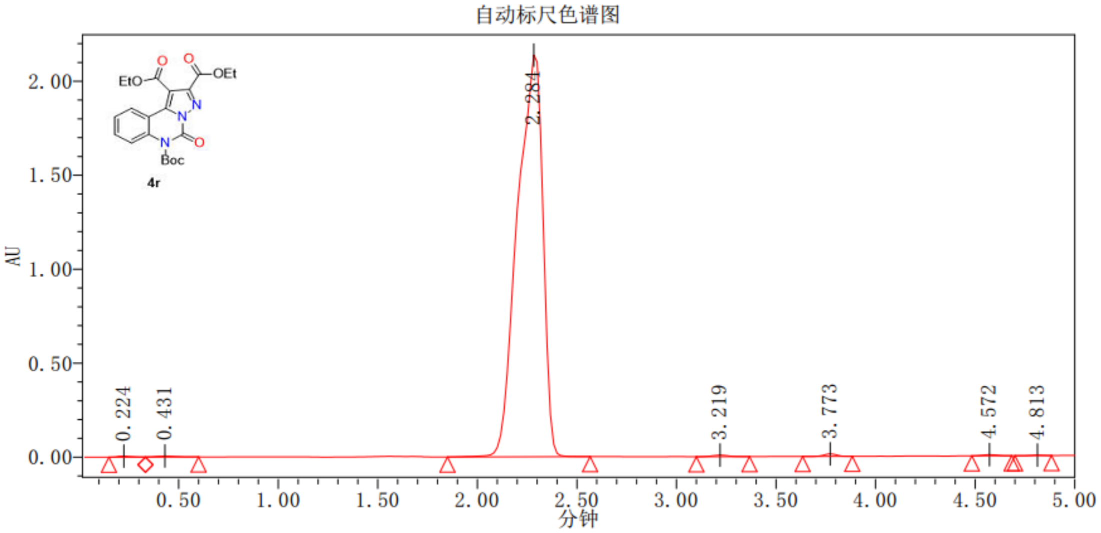

峰结果

	名称	保留时间（分钟）	面积（微伏*秒）	高度（微伏）	% 面积
1		0.224	32494	5929	0.17
2		0.431	41732	5555	0.22
3		2.284	18961877	2132894	98.86
4		3.219	45657	7958	0.24
5		3.773	59349	13185	0.31
6		4.572	28627	5779	0.15
7		4.813	11353	2646	0.06

自动标尺色谱图

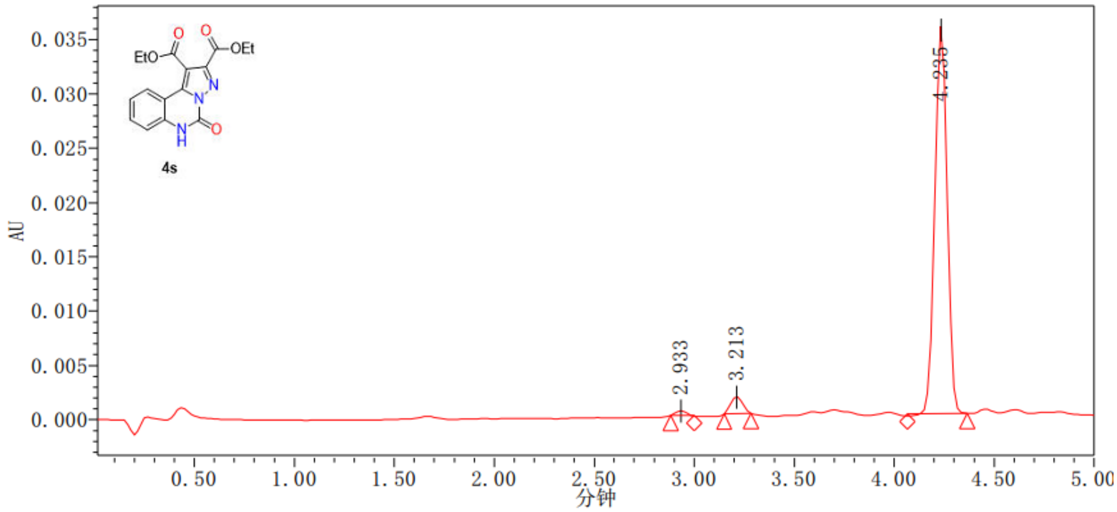

峰结果

	名称	保留时间（分钟）	面积（微伏*秒）	高度（微伏）	% 面积
1		2.933	1500	413	0.93
2		3.213	6526	1534	4.03
3		4.235	153787	34961	95.04

自动标尺色谱图

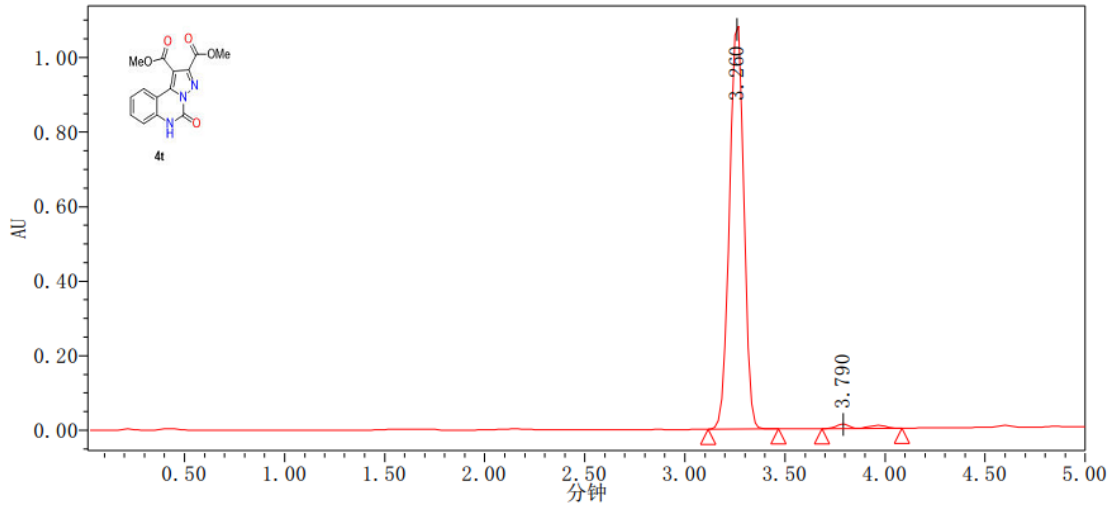

峰结果

	名称	保留时间（分钟）	面积（微伏*秒）	高度（微伏）	% 面积
1		3.260	5419606	1082464	98.13
2		3.790	103311	11592	1.87

自动标尺色谱图

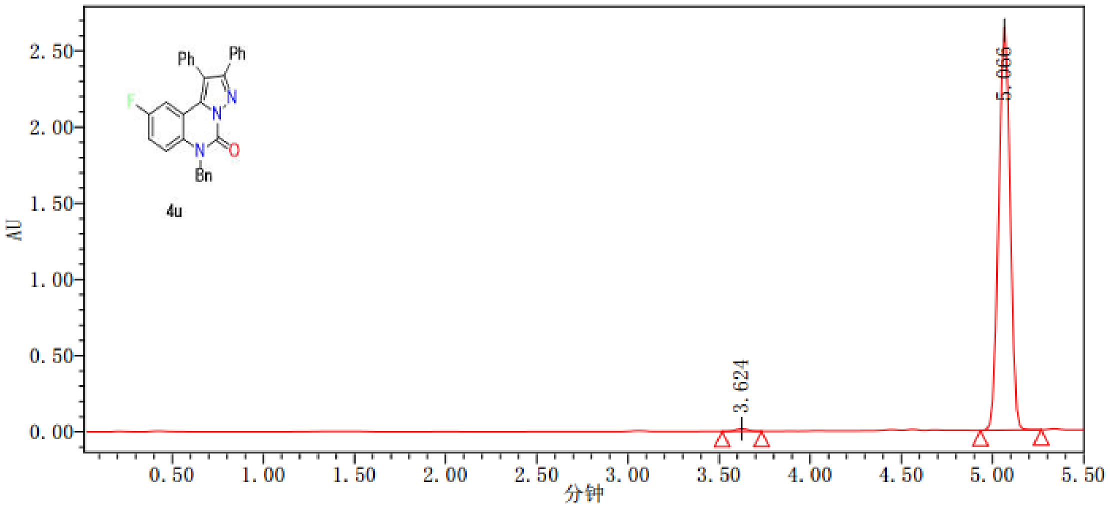

峰结果

	名称	保留时间(分钟)	面积(微伏*秒)	高度(微伏)	% 面积
1		3. 624	78130	17230	0. 67
2		5. 066	11537653	2607282	99. 33

自动标尺色谱图

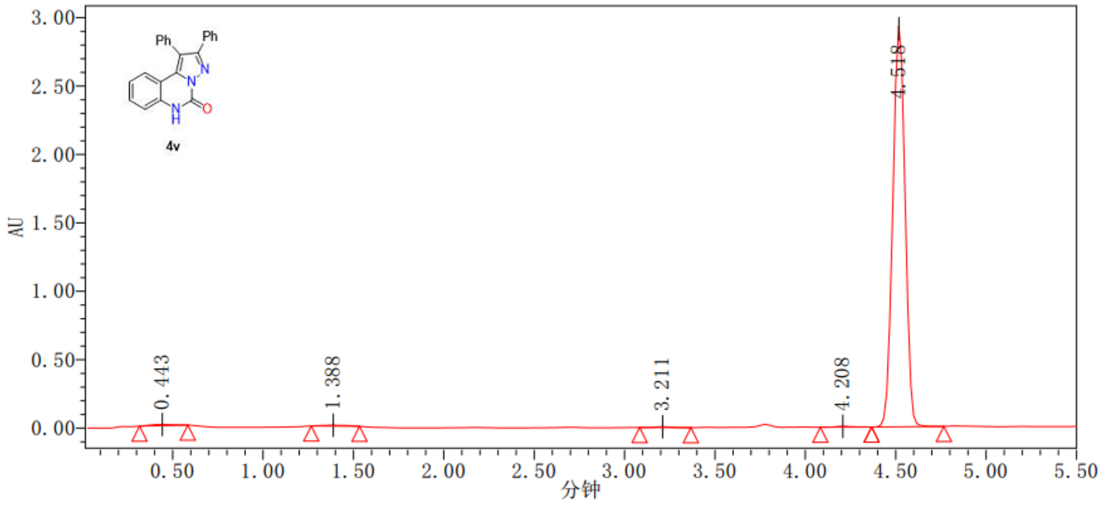

峰结果

	名称	保留时间(分钟)	面积(微伏*秒)	高度(微伏)	% 面积
1		0. 443	85568	9157	0. 61
2		1. 388	67415	6269	0. 48
3		3. 211	37500	5923	0. 27
4		4. 208	40741	6534	0. 29
5		4. 518	13865955	2909908	98. 36

7. Biology methods

6.1 Cell Growth Inhibition Assay

The effects of target compounds on cell proliferation were performed by Hainan Provincial Key Laboratory for Research and Development of Tropical Herbs (Hainan, China). The U-87 (glioblastoma) cell line was purchased from Procell (Wuhan, China). The A549 (non-small cell lung cancer), the MDA-MB-231 (breast cancer) and the HepG2 (hepatocellular carcinoma) cell lines were purchased from Haiyibo Biotech Co. (Haikou, China). The A549, the U-87, the MDA-MB-231 and the HepG2 cells were incubated with 10% FBS, 100 U mL^{-1} penicillin and 100 μg mL^{-1} streptomycin at 37 °C under a humidified atmosphere of 5% CO_2. The experimental procedure, using A549 cells as an example, involved culturing the cells until they reached the logarithmic growth phase. At this point, the cells were digested, counted, and diluted to a concentration of $2x10^4$ cells/mL. The peripheral wells of a 96-well plate were filled with phosphate buffer solution (PBS) to reduce experimental error caused by edge evaporation, while the remaining wells were inoculated with 100 μL of cell suspension and incubated for 24 hours. A 10 mM compound solution in DMSO was diluted to concentrations of 300/100/60/30/10/1/0.33 μM or 100/33.33/11.11/3.70/1.24/0.41 μM with growth medium and added to the wells containing cells. The plate was incubated for 72 hours before the supernatant was gently removed and replaced with 110 μL of complete medium containing 10% CCK-8. After incubating for an additional hour, the absorbance (OD) of each well at 450 nm was measured using an enzyme marker. Similar experimental methods were used for other tumour cells. The anti-proliferative activity of the compounds on the cells (% inhibition and IC_{50} values) were calculated using Prism 8.0 and EXCEL software.

6.2 Biochemical kinase activity assay

For the biochemical kinase activity assay, we used the CDK kinase activity assay developed by Caliper/LabChip EZ Reader (Perkin Elmer, Waltham, MA). The kinase activity rate and IC_{50} values of CDK2/7/9 were determined using the Mobility Shift Assay (MSA) platform at Biortus Biosciences. In this protocol, using the CDK9 kinase activity assay as an example, the concentration of phosphorylated peptide substrate produced as a fraction of total peptide activity is monitored following an incubation period of 60 minutes. Compounds 4t and 4n were assayed at concentrations ranging from 30 μM to 13.7 nM in a series of 3-fold serial dilutions and were incubated with

CDK9/Cyclin T1 complex (8 nM), ATP (0.03 mM), and "CDK9tide" peptide substrate (2 μM) in a buffer comprising 20 mM MES (pH 6.75), 10 mM MgCl2, 0.01% Tween 20, and 0.05 mg/mL BSA.

The procedure involved diluting the compounds (10 mM DMSO stocks) to make 1 mM DMSO stocks, followed by serial dilution to make 0.05 mM DMSO stocks. These were then diluted with ddH2O and transferred to a 384-well assay plate. CDK9/Cyclin T1 working solution was added to each well and pre-incubated for 60 minutes before adding the substrate mix. The plate was incubated at 27 ^{o}C for 60 minutes before quenching the reactions with EDTA and reading on a Caliper EZ Reader II. The IC_{50} values of CDK9 were calculated using Prism 8.0 and EXCEL software.

8. *In silico* study methods

7.1. *In silico* drug-likeness property and toxicity study

Pharmacokinetics and drug-likeness prediction ACD labs Chemsketch was used for generation of the chemical structures and SMILES notations of the final pyrazole derivatives. SMILES notations of the studied compounds were fed into the freely accessible web server Swiss ADME (http://www.swissadme.ch/) for prediction of the pharmacokinetic and drug-likeness aspects of the studied compounds [1]. This study depends on the chemical structure of the compounds which are involved in the calculation of certain parameters including human gastrointestinal absorption (HIA), blood-brain barrier (BBB) permeation, substrate, or non-substrate for the permeability glycoprotein (*P*-gp), $Logk_p$ and interaction of molecules with cytochromes P450 isomers (CYP).

For prediction of the possible toxicities like mutagenicity, tumourogenicity irritant and reproductive effect, SMILES notations of the studied compounds, generated by ACD labs Chemsketch version 11.01, were fed into another virtual filter Osiris Property Explorer[2].

7.2. The structure-based kinase profiling of the ProfKin web server works

Kinase profiling is an effective strategy for kinase inhibitor discovery, polypharmacological drug discovery, and drug repositioning. To fully exploit the potential of structure-based kinase profiling in these areas, Li's group developed a versatile web server called ProfKin. This server is based on an in-house comprehensive

structural database, KinLigDB, which contains manually curated kinase-ligand complex structures and associated information[3].

The structure-based kinase profiling approach behind the ProfKin web server works via integrating molecular docking and interaction fingerprinting methods. We uploaded the compound **4t** (the job ID 22122215005489) for predicting its kinase profile to all kinase structures, and to visually inspect the predicted binding pose as well as the superimposition of the predicted pose with the crystal ligand. The detailed results of ProfKin have been shown in the website (www.lilab-ecust.cn/profkin/profiling/results/22122215005489).

References

1. Daina, A.; Michielin, O.; Zoete, V. SwissADME: A free web tool to evaluate pharmacokinetics, drug-likeness and medicinal chemistry friendliness of small molecules. *Sci. Rep.* **2017**, *7*, 42717.
2. Sander, T. OSIRIS Property Explorer. Available online: https://www.organic-chemistry.org/prog/peo/ (accessed on 20 May 2023).
3. Shen, Z.; Yan, Y.-H.; Yang, S.; Zhu, S.; Yuan, Y.; Qiu, Z.; Jia, H.; Wang, R.; Li, G.-B.; Li, H. ProfKin: A comprehensive web server for structure-based kinase profiling. *Eur. J. Med. Chem.* **2021**, *225*, 113772.
